# Palaeogenomic insights into the origins of early settlers on the island of Cyprus

**DOI:** 10.1038/s41598-024-60161-z

**Published:** 2024-04-26

**Authors:** Alexandros Heraclides, Aris Aristodemou, Andrea N. Georgiou, Marios Antoniou, Elisabeth Ilgner, Leonidas-Romanos Davranoglou

**Affiliations:** 1 Department of Infectious Disease, Faculty of Medicine, Imperial College London, London, UK; 2 Department of Electrical and Computer Engineering, University of Thessaly, Volos, Greece; 3 School of Archaeology/Merton College, University of Oxford, Oxford, UK; 4 Oxford University Museum of Natural History, University of Oxford, Oxford, UK; 5 Present Address: School of Sciences, European University Cyprus, 6 Diogenis Str., 2404 Engomi, P.O. Box: 22006, 1516 Nicosia, Cyprus

**Keywords:** Structural variation, Structural variation

## Abstract

Archaeological evidence supports sporadic seafaring visits to the Eastern Mediterranean island of Cyprus by Epipaleolithic hunter-gatherers over 12,000 years ago, followed by permanent settlements during the early Neolithic. The geographical origins of these early seafarers have so far remained elusive. By systematically analysing all available genomes from the late Pleistocene to early Holocene Near East (c. 14,000–7000 cal BCE), we provide a comprehensive overview of the genetic landscape of the early Neolithic Fertile Crescent and Anatolia and infer the likely origins of three recently published genomes from Kissonerga-*Mylouthkia* (Cypriot Late Pre-Pottery Neolithic B, c. 7600–6800 cal BCE). These appear to derive roughly 80% of their ancestry from Aceramic Neolithic Central Anatolians residing in or near the Konya plain, and the remainder from a genetically basal Levantine population. Based on genome-wide weighted ancestry covariance analysis, we infer that this admixture event took place roughly between 14,000 and 10,000 BCE, coinciding with the transition from the Cypriot late Epipaleolithic to the Pre-Pottery Neolithic A (PPNA). Additionally, we identify strong genetic affinities between the examined Cypro-LPPNB individuals and later northwestern Anatolians and the earliest European Neolithic farmers. Our results inform archaeological evidence on prehistoric demographic processes in the Eastern Mediterranean, providing important insights into early seafaring, maritime connections, and insular settlement.

## Introduction

The Fertile Crescent, extending from the southern Levant to eastern Anatolia, and further east to Upper Mesopotamia and the Central Zagros, is the region where agriculture and the domestication of ungulates first emerged during the Pleistocene-Holocene transition in Western Eurasia (around 12,000 years ago)^1,2^. Prior to these major shifts in human subsistence strategies, archaeological evidence testifies to sporadic maritime trips made by hunter-gatherers from the core Fertile Crescent to the Eastern Mediterranean island of Cyprus during the Epipaleolithic (c. 10,500 years cal BCE)^3^. In the ensuing two millennia, groups of early Near Eastern farmers introduced agriculture to Cyprus, evidenced by tools, crops, livestock, and domesticated wild animals (e.g. cats and dogs) at sites on the island during the Initial/Early Aceramic Neolithic or Cypriot Pre-Pottery Neolithic^4^ (Cypro-PPNA and Cypro-PPNB, terms used throughout the present study)^4–8^.

Although developments during the Cypro-PPN period are well documented archaeologically^3,4,8,9^, the geographical origins of the earliest settlers in Cyprus have remained elusive^9–12^. The presence of suids at the Epipaleolithic site of Akrotiri-*Aetokremnos* over 12,500 years ago^3,13^ and the initial Neolithic zooarchaeological record, as well as assemblages of worked stone and buildings attested at initial Cypro-PPNA sites, have been interpreted as cultural expressions of Levantine groups^4,5,9,14^. Imported obsidian found at early Cypro-PPN sites (particularly during Cypro-PPNB), however, has been geochemically characterised as deriving from Central Anatolia^15^, indicating maritime contacts. Ancient maritime navigation data also put forward south-central Anatolia and the northwestern Levant as the two most plausible starting points of early seafaring journeys to and from Cyprus from the surrounding mainland^10–12,16^.

Archaeogenetics provide an additional line of evidence in long-standing debates of archaeological and historical processes. In a Near Eastern context, ancient DNA (aDNA) evidence revealed significant genetic heterogeneity in the early Neolithic Levant^17^, Anatolia^18,19^, Mesopotamia^20–22^, and further east in the Central Zagros^23^, and added significant support to the demic diffusion model for the spread of agriculture from Anatolia to mainland Europe^24–26^. In contrast, the maritime diffusion of Epipaleolithic and Neolithic populations in the Eastern Mediterranean has been inferred only indirectly^27^, due to the lack of ancient genomes from insular Eastern Mediterraneans, until recently. As a result, the possible genetic impact of Epipaleolithic and early Neolithic Mediterranean seafarers, as well as the maritime transmission of agriculture have remained largely unstudied archaeogenetically.

Here, we focus on three recently published human genomes^28^ from the Cypro-Late PPNB (Cypro-LPPNB) site of Kissonerga-*Mylouthkia* in western Cyprus, which we incorporate in a comprehensive spatiotemporal genomic re-evaluation of all Epipaleolithic and Neolithic Near Eastern populations available in the current aDNA record^21,22,29,30^, including genomes not available during the first publication of these Cypro-LPPNB samples^20^. We detect possible ancestral sources for the first seafaring settlers of Cyprus and provide an inferred timeframe for the most probable admixture event that gave rise to the tested Cypro-LPPNB, providing important insights into demographic dynamics and maritime connections in the Late Pleistocene–Early Holocene Eastern Mediterranean.

## Results

### Dating re-evaluation of the Cypro-LPPNB Mylouthkia samples and kinship analysis

The three genomes of interest (I4207/KMY1, I4209/KMYL2, I4210/KMYL3) were extracted^28^ from human remains found in well 133 at Kissonerga-*Mylouthkia*^31^. They were neither directly radiocarbon dated nor found in association with radiocarbon-dated fills (deposits) of well 133, dated to Cypro-LPPNB^31,32^. We use a Cyprus-wide period date, based on calibrated radiocarbon date ranges from multiple Cypro-LPPNB sites, to assign an approximate date to the undated samples from well 133: c. 7600–6800 cal BCE^4^. This is about half a millennium later than previously reported dates for these samples in the bioarchaeological literature^28,33^. Details on our dating rationale can be found in Methods and in Supplementary Information (Sect. 1, Supplementary Information Table 2).

In order to elucidate the genetic independence of the tested Mylouthkia samples, arising from their archaeological context, with different samples deriving from disarticulated human remains found in close proximity to each other in the excavation^34^ (see further in Supplementary Information, Sect. 1, subsection ‘stratigraphy of well 133’) but also to detect possible close kinship, we used the Relationship Estimation from Ancient DNA (READ)^35^ tool. Our analysis reveals that the three tested samples are not close (2nd degree or closer) relatives (Supplementary Table S8).

### Distal ancestry of Neolithic Cypriots in context of the Near Eastern genetic landscape during the late Pleistocene-early Holocene

In order to determine the exact ancestral sources of the analysed Cypro-LPPNB individuals, we mined all available genomes from the regions surrounding Cyprus during the late Pleistocene and early Holocene, spanning the Epipaleolithic (Anatolia and Levant) or Mesolithic (Zagros and Caucasus) eras and the early Aceramic (Anatolia) or Pre-Pottery (Levant, Upper Mesopotamia, Zagros) Neolithic, as well as the earliest Ceramic/Pottery Neolithic. These include important genomes not available when the Cypro-LPPNB Mylouthkia samples were first published, such as from Central Anatolian Aceramic Neolithic Musular^29^, Southeast Anatolian/Upper Mesopotamian Nevalı Çori^21^ and Çayönü Tepesi^22^, and the earliest available genome from Ceramic Neolithic northwestern Anatolia from the site of Aktopraklik^30^.

**Figure 1:**
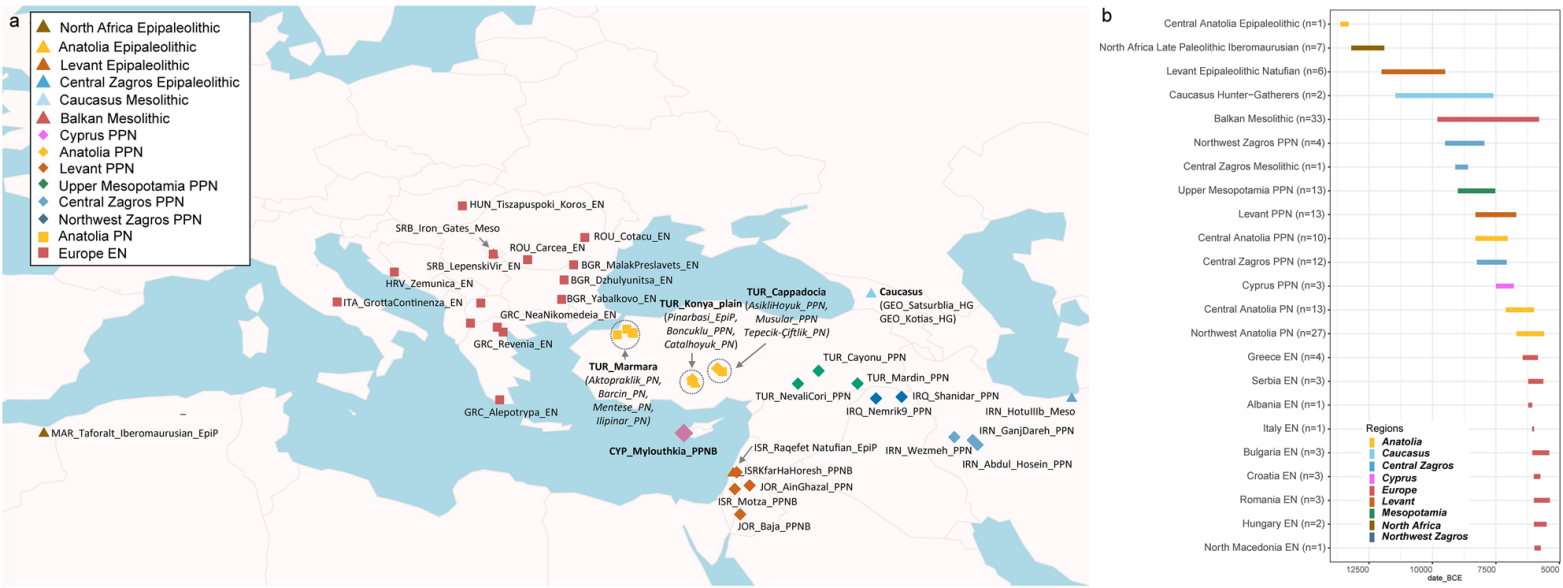
Geographical location and chronology of ancient samples used in the present study. (**a**) Geographical location of Epipaleolithic/Mesolithic and initial Neolithic archaeological sites in West Eurasia from which ancient samples were included in the present study. Sites and the corresponding samples are colour-coded and annotated by region and chronological period. (**b**) Chronology range (years BCE) and number of included individuals for all archaeological sites analysed, using the same colour-coding for regions as in panel a. Chronology abbreviations next to regions are as presented in Table 1. Country abbreviations can be found in Supplementary Information (Detailed Methods). The above information can be found in tabular form in Supplementary Tables 1 and 2.

Table 1 lists all analysed ancient population groups and Fig. 1 visualises their spatiotemporal context (see also Supplementary Table S1).

**Table 1:** Labels and basic characteristics for key populations included in the study.

***Epipaleolithic/Mesolithic***	
Anatolia_Central_Epip	Central Anatolian Epipaleolithic hunter-gatherer from Pınarbaşı Höyük, Konya plain, c. 14th millennium BCE^18^
Levant_Epip	Levantine Epipaleolithic Natufian hunter-gatherers from modern day Israel (Raqefet Cave), c. 12th–10th millennium BCE^17,28^
Zagros_Central_Meso	Central Zagros Mesolithic (possible) hunter-gatherer from modern day Iran (Hotu cave), c. 10th–9th millennium BCE^17,36^
CHG	Caucasus hunter-gatherers from modern day Georgia (Satsurblia cave, Kotias Klde rock shelter), c. 12th–8th millennium BCE^37^
NorthAfrica_LPal	North African Late Paleolithic Iberomaurusian hunter-gatherers from modern day Morocco (Taforalt cave), c. 14th–12th millennium BCE^38^
Europe_Balkan_Meso	Balkan Mesolithic hunter-gatherers from modern day Serbia (Vlasac), broader region of the Iron Gates, c. 10th–6th millennium BCE^39^
***Pre-Pottery/Aceramic Neolithic***	
Cyprus_PPN	Cypriot Late Pre-Pottery Neolithic B (Cypro-LPPNB) individuals from Kissonerga-*Mylouthkia*, Western Cyprus, c. 8th–7th millennium BCE^28^
Anatolia_Central_PPN	Central Anatolian Pre-Pottery (Aceramic) Neolithic farmers from the Konya plain (Boncuklu Höyük) and Cappadocia (Aşıklı Höyük, Musular), c. 9th–8th millennium BCE^18,19,29^
Levant_PPN	Levantine Pre-Pottery Neolithic B farmers from modern day Israel (Tel Motza, Kfar HaHoresh) and Jordan (‘Ain Ghazal, Ba'ja), c. 9th–7th millennium BCE^17,18,21,28^
Upper_Mesopotamia_PPN	Upper Mesopotamian Pre-Pottery Neolithic B farmers from Southeast Anatolia (Nevalı Çori, Çayönü Tepesi, Boncuklu Tarla - Mardin), c. 9th–8th millennium BCE^21,22,28^
Zagros_NorthWest_PPN	Northwest Zagros Pre-Pottery Neolithic A farmers from modern day Iraq (Nemrik 9, Shanidar Cave), c. 10th–8th millennium BCE^28^
Zagros_Central_PPN	Central Zagros Pre-Pottery Neolithic farmers from modern day Iran (Ganj Dareh, Tepe Abdul Hosein, Wezmeh Cave), c. 9th–8th millennium BCE^23,36^
***Pottery/Ceramic Neolithic***	
Anatolia_Central_PN	Central Anatolian Pottery (Ceramic) Neolithic farmers from the Konya plain (Çatalhöyük) and Cappadocia (Tepecik-Çiftlik), c. 8th–7th millennium BCE^19,25^
Anatolia_Northwest_PN	Northwest Anatolian Pottery (Ceramic) Neolithic farmers from the Marmara region (Barcın Höyük, Menteşe, Ilıpınar, Aktopraklık), c. 7th–6th millennium BCE^25,26,28,30,40^
Europe_EN	Very Early European Farmers (VEEF), comprising the earliest identified (before c. 5500 BCE) Neolithic individuals from Mediterranean Europe: modern day Greece (Revenia, Nea Nikomedeia, Alepotrypa cave) and Italy (Grotta Continenza); the Balkans: modern day Bulgaria (Yabalkovo, Dzhulyunitsa, Malak Preslavets), Romania (Coţatcu, Cârcea-Viaduct), Albania (Podgorie), North Macedonia (Cerje-Govrlevo), Serbia (Lepenski Vir), and Croatia (Zemunica cave); and Central Europe: modern day Hungary (Tiszapüspöki, Körös Basin), c. 7th–6th millennium BCE^26,30,39,41^

Chronological abbreviations used throughout the text and in Fig. 1: LPal (Late Paleolithic), Epip (Epipaleolithic), Meso (Mesolithic), PPN (Pre-Pottery/Aceramic Neolithic), PN (Pottery/Ceramic Neolithic), EN (Early Neolithic—relevant only to Europe), HG (hunter-gatherers), VEEF (very early European farmers).

**Figure 2:**
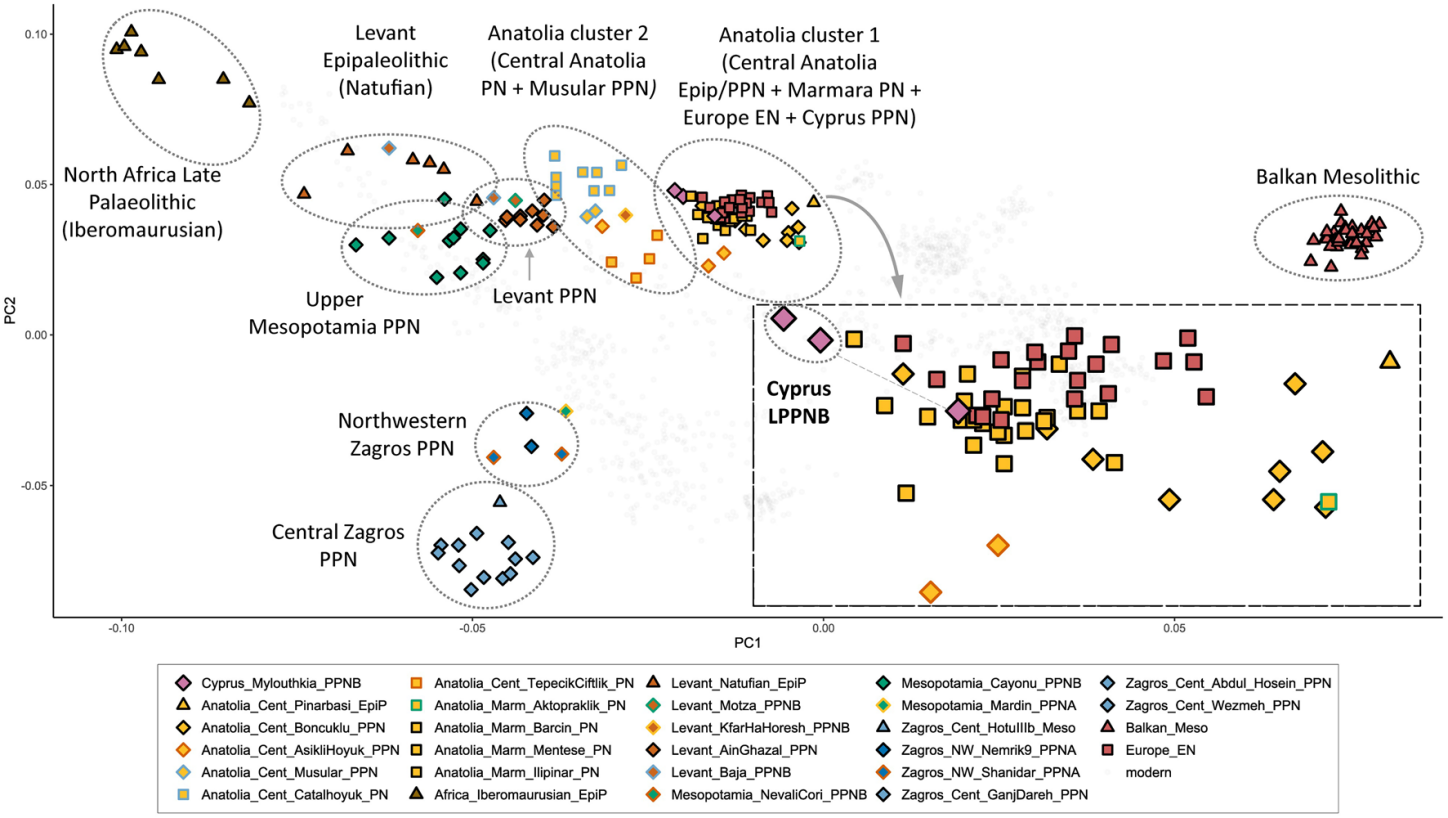
Principal Components Analysis (PCA) plot displaying all ancient samples analysed in the present study, projected on modern West Eurasian genetic variation. Ancient samples are presented as coloured data points, projected on modern West Eurasian populations (light grey points). The main panel displays the first two principal components with all Epipaleolithic/Mesolithic hunter-gatherer and initial Neolithic farming populations analysed in the present study. EpiP/Meso populations can be seen at the edges of the plot (Central Zagros / Caucasus on the bottom left, North African Late Palaeolithic on the top left, Balkan Mesolithic on the bottom right). Anatolian (yellow triangle) and Levantine (brown triangles) Epipaleolithic groups, appear more centrally in the plot and have high genetic proximity to Pre-Pottery Neolithic (PPN) populations from the same regions. Anatolian Neolithic populations form two distinct clusters, the first one appearing closer to the Epipaleolithic Pınarbaşı HG and comprises Central Anatolian PPN Boncuklu and Aşıklı Höyük, Marmara PN groups and Very Early European Farmers (VEEF), as well as Cypro-LPPNB. The second Anatolian cluster shows a shift towards Levantine EpiP/PPN populations and comprises Central Anatolian PPN Musular and PN population groups. Upper Mesopotamian PPN population groups show a clear shift towards Zagros Mesolithic/Neolithic populations. A zoomed-in version of the first Anatolian cluster seen as a separate panel within the main panel, displays Cypro-LPPNB (larger orchid diamonds) clustering closely with Anatolian Marmara PN groups (yellow squares), as well as the earliest Neolithic farmers of Europe (red squares). One Cypro-LPPNB sample (I4210) clusters entirely with PN Anatolian and EN European farmers, while the other two (I4207, I4209) very slightly shift towards Levantine groups, still however clearly belonging to the Anatolian cluster. All abbreviations in the plot as in Table 1.

To gain insights into the relationships between the analysed metapopulations, we conducted Principal Components Analysis (PCA) by projecting the above listed ancient samples onto modern Western Eurasian^42^ genetic variation (“Methods” section). Our PCA plot (Fig. 2) successfully captures the spatiotemporal ancient genetic variation of the Near East, southeastern Europe, and North Africa during the late Pleistocene and early Holocene, consistent with previous archaeogenetic analyses^17,18,24,39,40,43^. The three Cypro-LPPNB Mylouthkia individuals are clearly positioned in a core Anatolian cluster, with one clustering entirely with Pottery Neolithic Anatolian and Very Early European farmers (VEEF), while the other two shift slightly towards Levantine groups, still however clearly belonging to the specific Anatolian cluster (Fig. 2, zoomed-in panel). It should be noted here that precise PCA positioning should be interpreted with caution, as clustering also depends on SNP coverage^44^.

**Figure 3:**
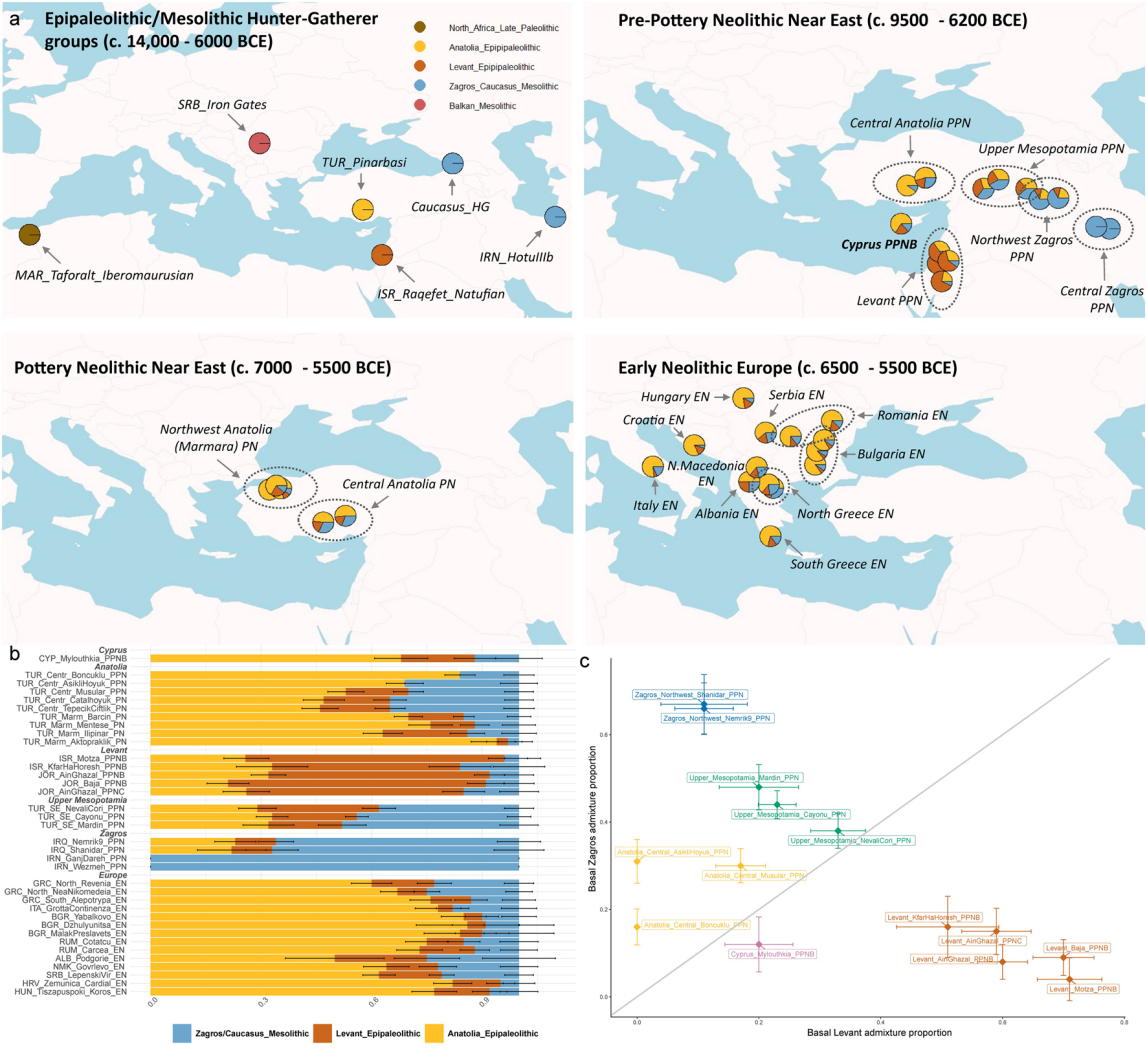
Distal genetic composition of ancient samples analysed in the present study in a spatiotemporal context. (**a**) Admixture weights derived using *qpAdm*, from a rotating model including five major, genetically distinct, ancestral Epipaleolithic/Mesolithic populations (Late Palaeolithic North African Iberomaurusian HGs, Epipaleolithic Central Anatolian HGs, Epipaleolithic Levantine Natufian HGs, Mesolithic Zagros/Caucasus HGs, Mesolithic Balkan HGs), separated by archaeologically defined periods and broad regions (Pre-Pottery Neolithic Near East, Pottery Neolithic Near East, Early Neolithic Europe), with chronological range of thus grouped samples in brackets (**b**) Bar plot displaying the same admixture weights grouped by region. Pre-Pottery Neolithic Near Eastern populations appear to derive all their ancestry from three of the five ancestral populations presented in panel a (Epipaleolithic Central Anatolian HGs, Epipaleolithic Levantine Natufian HGs, Mesolithic Zagros/Caucasus HGs). The Cypro-LPPNB group appears to derive 68% of its ancestry from Epipaleolithic Central Anatolians, 20% from Epipaleolithic Levantine Natufians, and 12% from Mesolithic/Neolithic Zagros. Anatolian PPN and PN population groups derive the majority of their ancestry from Epipaleolithic Central Anatolia (Pınarbaşı HG), with Central Zagros basal admixture apparent in PPN groups and additional Levantine basal admixture particularly apparent in PN groups. Levantine PPN population groups derive the majority of their ancestry from Epipaleolithic Natufians. Upper Mesopotamian and Northwest Zagros PPN population groups derive roughly equal ancestry from the three major components mentioned above, with excess basal Central Zagros admixture observed in the Northwest Zagros groups. Central Zagros PPN population groups share all of their ancestry with an earlier forager (Hotu IIIb) from the same region. The earliest European farmers have a similar profile to contemporaneous Northwest Anatolians. (**c**) Biplot of basal Levant to basal Zagros admixture, which helps differentiate PPN population groups, based on whether they have an excess Levantine relative to Zagros admixture or vice versa. Cypro-LPPNB belong to the former category. All abbreviations in the plot as in Table 1. All *qpAdm* models showing acceptable fit for all tested ancient populations, including exact admixture weights and detailed fit statistics, can be found in tabular form in Supplementary Table S3.

To determine whether genetic proximity between population groups captured in the PCA plot reflect shared ancestry and are not the result of projection artefacts, we formally tested the distal ancestry of all Pre-Pottery Neolithic and Pottery Neolithic populations listed in Table 1, which include recently published Near Eastern population groups not previously analysed in the context of revealing the ancestry of the Cypro-LPPNB samples of interest. By applying a rotating *qpAdm* model^45^, and by determining admixture components with relevant Epipaleolithic/Mesolithic population groups (Table 1) as potential sources (see “Methods”), we identify a three-way distal admixture model, comprising an Epipaleolithic Central Anatolian^18,19^ component, an Epipaleolithic Levantine^17^ component, and a Mesolithic/Neolithic Zagros^17,23^/Mesolithic Caucasus^37^ component, efficiently characterising the ancestral composition of all Near Eastern and earliest Neolithic European populations (Fig. 3, Supplementary Table S3). This model reveals a complex genetic landscape and dynamic spatiotemporal admixture processes in the Epipaleolithic and early Neolithic Near East, including previously undescribed local phenomena, such as differential Levantine and Zagros-related ancestry among Aceramic Central Anatolians. A detailed discussion on the genetic variation and admixture dynamics among all analysed Near Eastern populations is provided in Supplementary Information (Sect. 2).

In the context of this complex genetic landscape, the Cypro-LPPNB Mylouthkia individuals appear to derive approximately 68% of their distal ancestry from an Epipaleolithic Pınarbaşı-related source, 20% from Epipaleolithic Levant-related sources, and 12% from Mesolithic/Neolithic Zagros-related sources. A similar distal genetic profile was also suggested in the publication that first reported these samples, yet with slightly different source populations^20^. The newly identified deep ancestral profile of Cypro-LPPNB characterised by a predominant basal Anatolian component, with the additional presence of substantial Levantine-related ancestry and relatively low Zagros-related ancestry (Fig. 3, Supplementary Fig. S1), is unique among other potentially ancestral Pre-Pottery Neolithic farmers of the mainland, (Fig. 3; Supplementary Table S3), and might indicate demographic processes unique to Cyprus, or alternatively, unsampled diversity in the evident Anatolian-Levantine/Zagros cline. From populations included in the first Anatolian cluster (Fig. 2), only Epipaleolithic Pınarbaşı and Aceramic Neolithic Boncuklu and Aşıklı Höyük could be considered as potentially ancestral to Cypro-LPPNB Mylouthkia, as the included Ceramic Neolithic Marmara groups postdate Cypro-LPPNB by roughly half to one millennium (Fig. 1; Supplementary Table S1).

### Genetic affinity between Cypro-LPPNB Mylouthkia and surrounding populations

**Figure 4:**
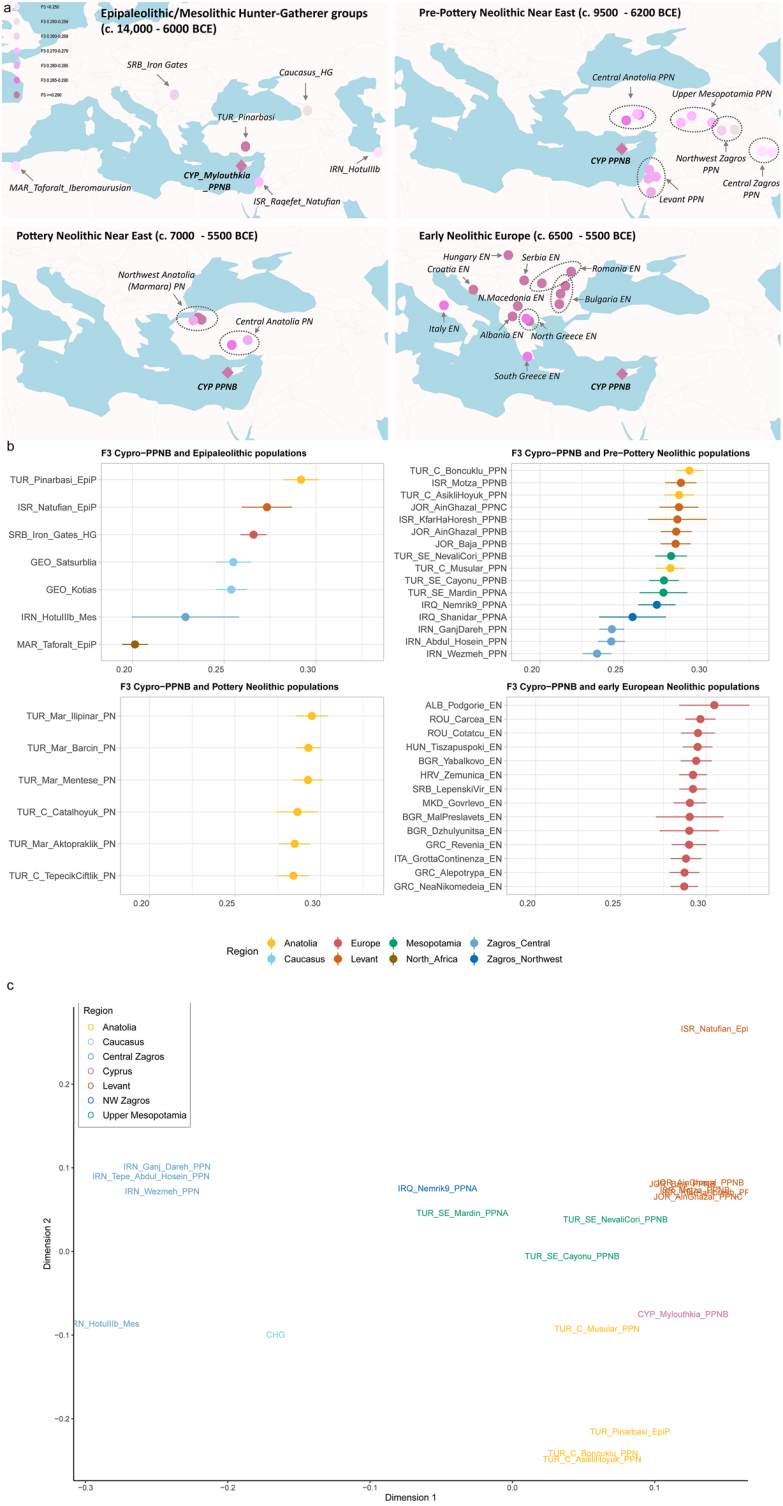
Outgroup *f3*-statistics displaying shared genetic drift between all ancient samples analysed in the present study and Cypro-LPPNB samples in a spatiotemporal context. (**a**) The four-panel map displays outgroup *f3*-statistics of the form *f3(Mbuti; Cypro-LPPNB, comparison ancient population)* estimating shared genetic drift, based on allele sharing. Each analysed population is displayed in its corresponding location on the map, with the colouring of the symbols representing the genetic proximity between the corresponding ancient population and Cypro-LPPNB farmers (orchid diamond). The darker the colour, the higher the pairwise *f3* statistic between Cypro-LPPNB and the corresponding ancient population group, signifying higher allele sharing. (**b**) Actual value of each pairwise *f3* statistic (± 3 standard errors) between Cypro-LPPNB and all tested ancient populations. The further to the right the points are in each plot, the higher the allele sharing with Cypro-LPPNB. Wider error bars indicate lower precision, as a result of smaller number of available SNPs in the given pairwise combination. Colour-coding represents geographical regions as denoted in Fig. 1. Cypro-LPPNB show high allele sharing with Central Anatolian Epipaleolithic Pınarbaşı HG and Central Anatolian PPN Boncuklu and Aşıklı Höyük, Northwest Anatolian PN Marmara groups, and the earliest Neolithic European farmers. *f3* results with low precision (e.g. HotuIIIb) should be interpreted with caution, due to low number of available SNPs. All abbreviations in the plot as in Table 1. The displayed information can be found in tabular form in Supplementary Table S4. (**c**) MDS plot based on pairwise ‘1 minus outgroup *f3*-statistics’ of the form *f3* (Mbuti; test, comparison). Distances between data points represent genetic differentiation based on allele sharing among Mesolithic/Epipaleolithic and Pre-Pottery Neolithic Near Eastern population groups analysed in the present study. Cypro-LPPNB Mylouthkia, cluster in the middle of a genetic cline between Epipaleolithic and early Neolithic Anatolians (Pınarbaşı HG, Boncuklu and Aşıklı Höyük) and contemporaneous Levantine groups. An apparent close genetic proximity between Mylouthkia and Anatolian Musular, is not supported by *qpAdm* analyses, due to the much higher admixture from Zagros-related sources among the latter. All abbreviations in the plot as in Table 1. A matrix of raw genetic distances of the form 1 minus *f3* can be found in Supplementary Table S5.

Figure 4a displays genetic affinities between Cypro-LPPNB Mylouthkia and other Epipaleolithic/Mesolithic and Neolithic Near Eastern and southeastern European populations, based on allele-sharing using outgroup *f3-*statistics of the form *f3(Mbuti; Cypro-LPPNB, comparison ancient population)*. Figure 4b presents the same information hierarchically from closest to most distant population, by different chronological ranges (also found collectively in Supplementary Table S4 and Supplementary Fig. S2). The Central Anatolian Pınarbaşı HG displays by far the highest allele sharing with Cypro-LPPNB compared with all other Epipaleolithic/Mesolithic populations. This adds further support to our PCA and distal admixture analyses (Figs. 2 and 3, respectively), suggesting that the tested Mylouthkia group derives its ancestry primarily from Anatolia. Aceramic Neolithic Anatolian Boncuklu Höyük, appearing in the same cluster with Mylouthkia in the PCA plot, also show high allele sharing with them.

An MDS plot of pairwise distances of the form 1-*f3*(Mbuti; pop1, pop2) between all Near Eastern and European population groups of interest (Supplementary Fig. S3) adds further support to the above findings and corroborates our PCA plot, displaying Cypro-LPPNB as clustering with Anatolian and the earliest European Neolithic farmers, with a slight shift towards Levantine groups, as expected based on our *qpAdm* models.

Our findings receive additional support through *f4-*statistics of the form *f4(Mbuti, Cypro-LPPNB; comparison ancient population A, comparison ancient population B)*, which help pinpoint potential ancestral sources for Cypro-LPPNB Mylouthkia based on relative allele sharing (Supplementary Table S6 and Supplementary Fig. S4). In more detail, allele sharing between Cypro-LPPNB and potentially ancestral Near Eastern sources increases gradually from the Central to northwestern Zagros, to Upper Mesopotamia, the Levant, and peaks in Anatolia (Supplementary Fig. S4d). Within Anatolia (Supplementary Fig. S4a), Epipaleolithic Pınarbaşı and Aceramic Neolithic Boncuklu and Aşıklı Höyük appear to share roughly equal numbers of alleles with Cypro-LPPNB, while allele sharing decreases significantly in the presence of higher Zagros admixture, as apparent in PPN Musular (Fig. 3, Supplementary Table S4). Among Zagros, Upper Mesopotamian, and Levantine populations (Supplementary Figs. S4b/c), allele sharing with Cypro-LPPNB appears to be driven by increased Anatolian-related admixture and decreased Zagros-related admixture (Fig. 3; Supplementary Table S3).

These findings suggest that the Anatolian input to Cypro-LPPNB Mylouthkia may stem from populations not yet influenced by the substantial Central Zagros-related admixture already apparent in Aceramic Central Anatolian Aşıklı Höyük and Musular, which also characterises later Ceramic Anatolian groups, such as Çatalhöyük and Tepecik-Çiftlik (Fig. 3, Supplementary Table S3). Aceramic Neolithic Boncuklu, with a moderate Zagros-related ancestry of ~ 15%, is an example of such a population group.

### Proximal ancestry of Cypro-LPPNB Mylouthkia

To formally test the genetic input of different Epipaleolithic and PPN groups into Cypro-LPPNB, we used a rotating models approach in *qpAdm*^45^ and derived admixture proportions for Mylouthkia as a target, ‘blindly’ testing all possible combinations of all Near Eastern Epipaleolithic and PPN populations as sources (Supplementary Table S7). From population groups potentially ancestral to Mylouthkia, which display significant allele-sharing with them (Fig. 4; Supplementary Table S4), *qpAdm* does not statistically accept (p-value < 0.05) any as a single source, contrasting previous findings indicating that Aşıklı Höyük could act as a single source for Mylouthkia^20^. A probable reason for this discrepancy, is that the set of Aşıklı samples used in our analysis (ASH136, ASH129, and the high coverage ASH128) is different from that previously used (ASH129, ASH131, ASH136, the latter two being siblings and all being low coverage genomes). Our results are further supported by the distal genetic composition of Cypro-LPPNB (68% Epipaleolithic Anatolia, 20% Epipaleolithic Levant, 12% Mesolithic/Neolithic Zagros) and Aşıklı Höyük (69% Epipaleolithic Anatolia, 31% Mesolithic/Neolithic Zagros), which differ substantially (Fig. 3, Supplementary Table S3).

**Figure 5:**
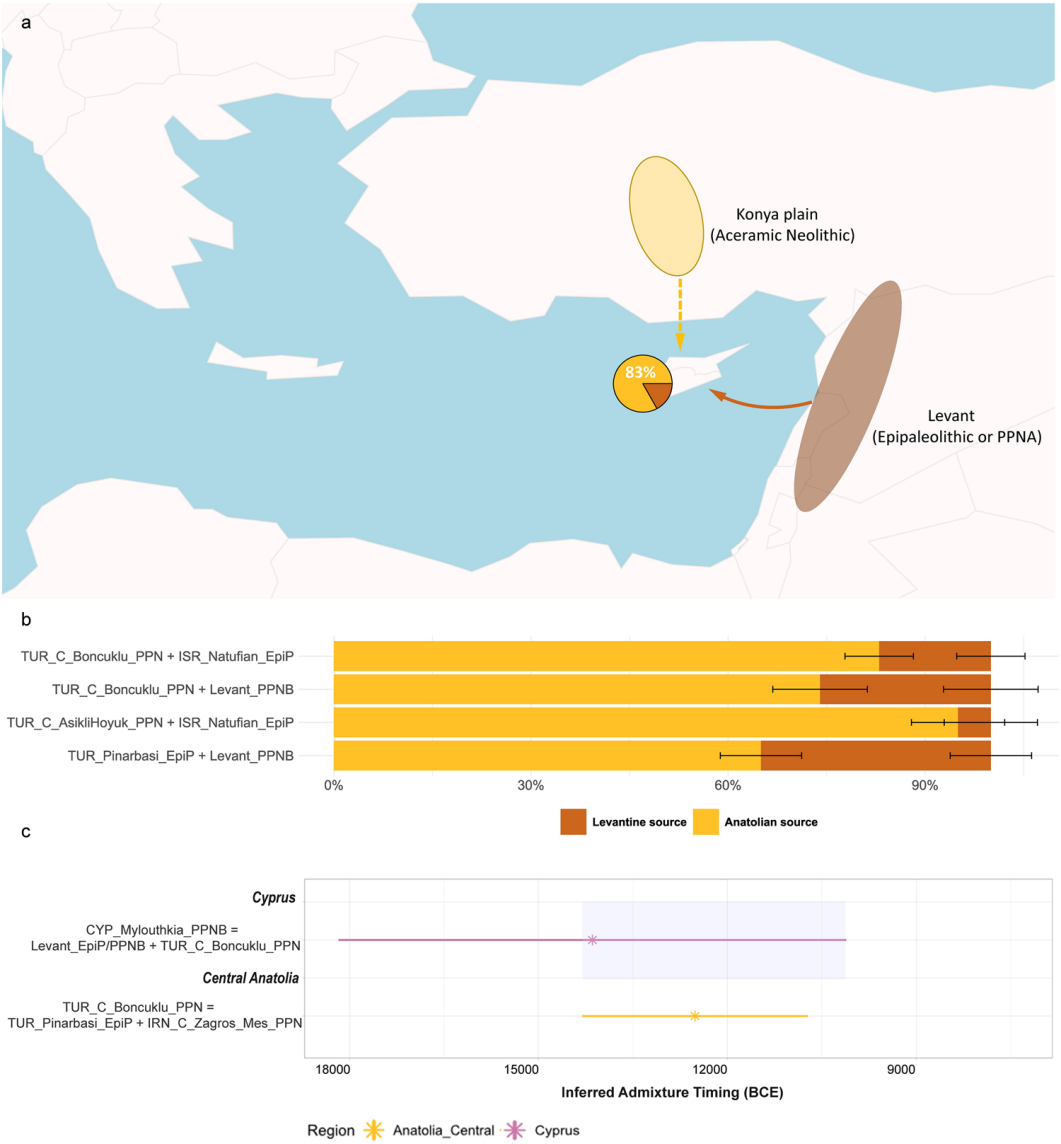
Proximal genetic composition of Cypro-LPPNB farmers in a spatiotemporal context, estimated from *qpAdm* analysis, with all major Near Eastern Epipaleolithic and PPN populations (potentially ancestral to Cypro-LPPNB) as sources. (**a**) The presented proximal admixture composition comprising 83% ancestry from Central Anatolian Boncuklu and 17% from Epipaleolithic Levantine Natufians, provides the best fit from all tested models. The denoted regions (Konya plain and Levant) are approximations and not meant to precisely represent the actual archaeological regions. Similarly, arrows aim to provide an overall understanding of the possible migration flow from the mainland to the island of Cyprus and are not meant to represent exact departure and arrival locations. All *qpAdm* models involving potentially ancestral sources for Cypro-LPPNB as target, including exact admixture weights and detailed fit statistics, can be found in tabular form in Supplementary Table S7. (**b**) The four best fitting two-way proximal admixture models for Cypro-LPPNB as target. The first and second models present an admixture profile primarily deriving from Aceramic Neolithic Central Anatolian Boncuklu and only differ in the Levantine source detected (Epipaleolithic Natufian *vs* PPNB), while the third and fourth pick up Aceramic Neolithic Aşıklı Höyük and Epipaleolithic Pınarbaşı, respectively, as possible Anatolian sources, with additional input from Levantine groups. Further admixture timing analysis based on genome-wide weighted ancestry covariance favours the first model over the other three (Supplementary Table S11). (**c**) Inferred admixture timing using *DATES*, revealing a wide time range for the event giving rise to Cypro-LPPNB Mylouthkia (14,000 ± 4,000 years BCE), due to the low coverage of the relevant genomes. The same analysis reveals an admixture timing of 12,500 ± 1,700 years BCE for Aceramic Neolithic Anatolian Boncuklu. Combining these two findings and provided that all lines of evidence generated in the present study point to a Boncuklu-related group as the major ancestral source for Cypro-LPPNB Mylouthkia, it could be inferred that the admixture event for the latter took place roughly between 14,000 and 10,000 BCE (grey shaded time range), during the late Epipaleolithic or the transition to the Pre-Pottery Neolithic A (PPNA).

Our rotating outgroups approach detect four plausible two-way admixture models for Cypro-LPPNB Mylouthkia: (1) a major component (83%) from Aceramic Neolithic Anatolian Boncuklu and a minor component (17%) from Epipaleolithic Levantine Natufians (p = 0.463, SE = 0.052); (2) a major component (74%) from Aceramic Neolithic Anatolian Boncuklu and a minor component (26%) from PPNB Levantine sources (p = 0.413, SE = 0.072); (3) a major component (95%) from Aceramic Neolithic Anatolian Aşıklı Höyük and a minor component (5%) from Epipaleolithic Levantine Natufians (p = 0.420, SE = 0.071); and (4) a major component (65%) from Epipaleolithic Anatolian Pınarbaşı and a minor component (35%) from PPNB Levantine sources (p = 0.065, SE = 0.062) (Fig. 5b, Supplementary Table S7). A small number of additional three-way models receive some statistical support; however their fit is not better than the aforementioned two-way models, therefore the latter are kept, on grounds of parsimony.

Although it is difficult to single out any of the aforementioned two-way models as the most plausible, particularly in the presence of low coverage genomes making the results less reliable, we argue that in statistical terms, the first model comprising Central Anatolian Boncuklu and Epipaleolithic Levantine Natufians as proximal sources, does have a slight edge over the other three, given the relatively higher standard errors in the second and third, and the marginal p-value in the fourth model. The first model is also consistent with our distal admixture estimation for Cypro-LPPNB Mylouthkia, which revealed ancestry primarily from Anatolia and the Levant (Fig. 3, Supplementary Table S3), and an additional minor component (12%) from a Zagros-related source, which is in fact already present in Boncuklu farmers (successfully modelled as 84% Epipaleolithic Anatolia plus 16% Mesolithic/Neolithic Zagros). In contrast, nearby Aşıklı Höyük farmers harbour much higher Zagros admixture (Supplementary Table S3), inconsistent to that present among Mylouthkia.

Regarding the minor Levantine component, our findings cannot definitively exclude a Neolithic rather than Epipaleolithic input. However, later PPNB populations provide slightly inferior fits than models with Natufians as a source for Cypro-LPPNB (Supplementary Table S7). Among the tested PPNB Levantine proxies, Ba'ja provides the best fit, with this group comprising two genomes, one of which clusters with Natufians in the PCA plot (Fig. 2). Furthermore, in our rotating distal ancestry models, Late Palaeolithic North African Iberomaurusians come up as an alternative borderline acceptable source for Mylouthkia in the place of a Levantine source (Supplementary Table S7), supporting again a more basal Levantine component among Cypro-LPPNB, indicated by the high degree of deep ancestry shared between Natufians and Iberomaurusians^38^.

Given that no plausible single source model involving any PPN/Aceramic Near Eastern population could be identified for Cypro-LPPNB, we additionally tested later (postdating Cypro-LPPNB) Anatolian groups found to cluster with Mylouthkia (c. 7600–6800 BCE) (Fig. 2, Supplementary Fig. S3), as potential sources, namely Ceramic Neolithic northwestern Anatolian Aktopraklik (c. 6700–6500 BCE) and Barcin (c. 6500–5900 BCE), and Central Anatolian Çatalhöyük (c. 7100–6000 BCE). These analyses show that Ceramic Neolithic Barcin and Çatalhöyük could act as single sources for Mylouthkia, while in the case of the former a two-way model comprising 95% Barcin and 5% Natufian is also accepted statistically. Similar models were also reported by the original publication on the Mylouthkia samples^20^. Despite the statistical support for these models, we caution against considering them as representing the actual ancestry of the tested Cypro-LPPNB individuals. The main reasons are: (i) the sampled Cypro-LPPNB predate the aforementioned Ceramic Anatolian groups by at least half a millennium^46^; (ii) as regards Ceramic Neolithic Central Anatolians, the distal genetic profile of Çatalhöyük (47% Epipaleolithic Anatolia, 18% Epipaleolithic Levant, 35% Mesolithic/Neolithic Zagros) is substantially different from that of Cypro-LPPNB (Supplementary Table S3); (iii) population groups from the same regions (northwestern and central Anatolia) predating Barcin (e.g. Aktopraklik) and Çatalhöyük (e.g. Boncuklu) are not identified by our analyses as plausible single sources (Supplementary Table S7); and (iv) as regards Ceramic Neolithic northwestern Anatolians, none of the different archaeological reviews and simulations for ancient maritime connections between Cyprus and the surrounding mainland during the late Pleistocene–early Holocene^9–12,16^ detect the Marmara region as a plausible origin, apparently due to long distance, and unfavourable wind patterns and currents.

### Additional evidence based on admixture graphs

In order to further investigate ancient admixture dynamics giving rise to Cypro-LPPNB Mylouthkia and provide an additional line of evidence on the most probable direct ancestral sources, we applied *findGraphs*^47^ and *qpGraph*^48,49^, and through a rigorous investigation involving automatically fitting and testing over a thousand possible graphs, we detected a set of automatically derived admixture graphs (Supplementary Fig. S6, Supplementary Table S10), involving four admixture events between the included populations (see “Methods” section), with a good fit and a graph topology consistent with the overall admixture dynamics revealed by previous archaeogenetic evidence, as well as with our *qpAdm* results. Although we present additional good fitting graphs involving more admixture events (five) (Supplementary Fig. S7, Supplementary Table S10), we focus on the most parsimonious models (e.g. graphs with four admixture events in our case), as more complex models have the tendency to overfit and include implausible admixture events^47^, which was also the case in our analysis (Supplementary Fig. S7).

The derived admixture graphs support the involvement of a population group closely related to Central Anatolian Boncuklu as directly ancestral to Cypro-LPPNB Mylouthkia, also picking up a minor, more distal Levantine input related to Epipaleolithic Natufians, rather than Levantine PPNB. Since Central Anatolian Aşıklı Höyük also gave a statistically plausible admixture model, we repeated the above analysis including this as the Central Anatolian source in the place of Boncuklu. Unlike the case of Boncuklu, Aşıklı Höyük was not automatically picked up as a potential ancestral source of Mylouthkia (Supplementary Fig. S8, Supplementary Table S10).

### Comparison with the ancestry of contemporaneous populations from the mainland

To test the robustness of the above findings and to determine whether the two-way admixture inferred in our analyses is unique to Cypro-LPPNB Mylouthkia or also characterises other roughly contemporaneous Neolithic groups with similar genetic composition, we ran rotating models in *qpAdm* with PN Marmara groups (Barcin, Menteşe, Ilıpınar) and PN Central Anatolian Çatalhöyük as targets. These analyses reveal different ideal models for these groups, compared to Mylouthkia, with the former successfully fitting as a two-way model comprising a major Epipaleolithic Anatolian component and a minor component from Upper Mesopotamia (particularly the site of Boncuklu Tarla—Mardin) and the latter marginally modelled as a two-way mix between Aşıklı Höyük and PPNB Levant (Supplementary Table S9).

Interestingly, the best fitting models for PN Anatolia Marmara and Central Anatolia Çatalhöyük are rejected for Cypro-LPPNB as target and vice versa (Supplementary Table S9), providing further evidence for a distinct admixture event giving rise to Mylouthkia not characterising other contemporaneous mainland groups. This finding also provides evidence against the notion that Mylouthkia might be directly descending from an Aceramic Neolithic Anatolian group, at least not from those available in the current archaeogenetic literature.

Along the same lines, we used *findGraphs* to determine whether the derived admixture graphs involving Cypro-LPPNB Mylouthkia reveal unique and specific admixture dynamics, by repeating the above-described automated approach including Central Anatolian (Çatalhöyük) and Marmara Anatolian (Barcin) groups in the place of Mylouthkia in the graph topology (with the number of admixture events set to 4). This resulted in a deterioration of the model fit (larger LL and WR scores) and admixture events inconsistent with current archaeogenetic evidence (Supplementary Fig. S9, Supplementary Table S10). This further supports the notion that the specific admixture dynamics giving rise to Cypro-LPPNB Mylouthkia revealed in our best fitting automated admixture graphs (Supplementary Fig. S6), are unique and do provide a plausible scenario for their ancestry.

### Inferred admixture timing

To shed more light on the admixture event that gave rise to Cypro-LPPNB, we applied the Distribution of Ancestry Tracts of Evolutionary Signals (*DATES*) tool^50^ and inferred the timing of the four plausible two-way admixture models noted above. Due to the low coverage of the Mylouthkia genomes, the method returned a broad range of dates, which however still help to elucidate their ancestry to some extent (Supplementary Table S11). In particular, two plausible admixture models, involving a major component from Central Anatolian Aceramic Neolithic Boncuklu and a minor component from Epipaleolithic or PPNB Levant, respectively, provide plausible admixture timings in the range of c. 14,000 ± 4000 years BCE. This range is provided irrespective of whether Epipaleolithic Natufian, or PPNB groups, or their combination, are used as the Levantine ancestral source of choice, highlighting the robustness of *DATES* in estimating the timing of potentially unknown past admixture events, based on specific proxy ancestral populations.

Another model comprising a major Epipaleolithic Anatolian component and a minor PPNB Levantine component is estimated to have occurred c. 20,000 ± 5000 years BCE, which seems unlikely, given that this range predates the existence of Neolithic Levantine groups. An additional good fitting admixture model for Mylouthkia comprising Aceramic Neolithic Aşıklı Höyük and Epipaleolithic Natufians, did not provide valid admixture timings (e.g. negative number of generations) (Supplementary Table S11).

To derive a more comprehensive timeframe of admixture events relevant to the ancestry of Cypro-LPPNB in the surrounding mainland, we repeated the above *DATES* analysis with relevant Near Eastern early Neolithic populations as targets. In agreement with previous findings^50^, our analysis reveals that the first influx of Neolithic groups from the Zagros to Central Anatolia started occurring c. 12,500 ± 1700 years BCE, initially appearing in the Konya plain at sites such as Boncuklu, followed by the Cappadocian sites of Aşıklı Höyük and Musular, a couple of thousand years later. Our analysis estimates that this admixed Anatolian-Zagros profile had reached the Levant around 10,000 ± 700 years BCE, admixing with Natufian-like populations and giving rise to currently sampled Levantine PPNB groups.

Given that the influx of the Zagros-related admixture in Anatolia or the Levant is estimated not to have happened earlier than c. 14,000 BCE, this could be used as the earliest margin for the timing of the admixture event giving rise to Cypro-LPPNB Mylouthkia, who harbour this genetic component (Supplementary Fig. S10, Supplementary Table S11).

To test whether the inferred admixture timing for Mylouthkia is unique to this population group, we tested the same two-way admixture for Neolithic Marmara groups as target, given that these have a similar genetic profile to Mylouthkia, as well as high genetic affinity with them. This analysis gave a much later admixture date of c. 9600 ± 780 years BCE, indicating that the two population groups (Cypro-LPPNB and Marmara PN) acquired Levantine and Zagros ancestry from distinct admixture events, with the admixture being earlier in Neolithic Cyprus compared to northwestern Anatolia.

Overall, the plausible admixture timeframe for Cypro-LPPNB (c. 14,000 to 10,000 BCE) as estimated in our analyses, falls well into the Near Eastern Epipaleolithic (c. 18,000–10,000 BCE) and the transition to the Pre-Pottery Neolithic A (PPNA) in the Levant (c. 10,000–8500 BCE)^51^, just before the start of the Cypro-PPNA (c. 9000–8500/8400 BCE) (Supplementary Information, Table 1). Considering these findings, the admixture model involving later PPNB Levantines (c. 8500–6500 BCE)^51^ as the possible minor ancestral source for Cypro-LPPNB, receives less support in favour of the model involving Levantine Epipaleolithic (or currently unsampled PPNA) groups.

### Genetic affinity between Cypro-LPPNB and the earliest European Neolithic farmers

The outgroup *f3* analyses presented above (Fig. 4; Supplementary Table S4) revealed, for the first time, high genetic affinity between Cypro-LPPNB and very early European farmers (VEEF) from the Balkans (Albania, Bulgaria, Croatia, North Macedonia, Romania, Serbia), Central Europe (Hungary) and the eastern/central Mediterranean (Greece and Italy).

To determine whether Cypro-LPPNB or Epipaleolithic/Neolithic Anatolian populations are more closely related genetically with VEEF and/or whether a previously unidentified shared genetic drift occurred through allele sharing between these populations, we applied *f4-*statistics of the form *f4(Mbuti, VEEF; Cypro-PPNB, Anatolian population)* (Supplementary Fig. S11; Supplementary Table S12). We find that Cypro-LPPNB display higher allele-sharing with VEEF than the majority of Anatolian populations, with the exception of Pınarbaşı HG and Ceramic Neolithic Marmara groups (Barcin, Menetse, Ilipinar), which all share roughly equal numbers of alleles with VEEF as Mylouthkia.

Overall, our *f4* analysis supports a high genetic affinity between Cypro-LPPNB and the earliest Neolithic farmers of Europe, which is also highlighted by the similar distal ancestry composition of these groups (Fig. 3, Supplementary Table S3).

## Discussion

Our comprehensive analysis of all available ancient genomes from the late Pleistocene–early Holocene Fertile Crescent and Anatolia pinpoints the possible ancestral origins of a recently published group of Cypriot Pre-Pottery Neolithic B (Cypro-LPPNB) samples from Kissonerga-*Mylouthkia*^28,32^ (c. 7600–6800 cal BCE) in western Cyprus. Despite the low coverage of these samples, our analyses confirm previous suggestions^20^ of dual Anatolian-Levantine origins for these early insular settlers and do not support plausible direct ancestry from any single Epipaleolithic or PPN Near Eastern source, available on the current aDNA record. Our analysis pinpoints the proximal ancestry of these early Neolithic Cypriot farmers as likely deriving from a two-way admixture between an Aceramic Neolithic Central Anatolian source (possibly residing in or near the Konya plain) and an earlier (Epipaleolithic or initial Neolithic) Levantine source (Fig. 5). The admixture event between these two sources is estimated, based on our analyses, to have taken place between 14,000 and 10,000 BCE, encompassing the late Pleistocene to early Holocene transition and coinciding with the later Epipaleolithic and initial Aceramic (Pre-Pottery) Neolithic in the surrounding mainland. The primarily Aceramic Neolithic Anatolian ancestry for the tested Cypro-LPPNB is also supported by the uniparental marker evidence, as reviewed in our study (Supplementary Figs. S12, S13; Supplementary Information, Sect. 3).

The early presence of Levantine groups on Cyprus, is supported by previous archaeological evidence (further details in Supplementary Information, Sect. 5) pointing to the Levant as the most likely homeland of Epipaleolithic hunter-gatherers who visited Cyprus, since at least the mid-11th millennium BCE, as documented at the site of Akrotiri-*Aetokremnos*^3^. These early ventures were followed by settlements on Cyprus during the early Holocene (first half of the 9th millennium BCE), with the emergence of organised PPNA villages of hunter-cultivators (e.g. Ayios Tychonas-*Klimonas*^14^), presenting cultural parallels with PPNA Levant (chipped stone assemblage and relevant tools, as well as domestic architecture)^6,8,9,14^. A lithic assemblage from the Levant was also reported at Kissonerga-*Mylouthkia*^47^. Additionally, zooarchaeological evidence supports a Levantine origin of managed suids (already present on Cyprus from the Epipaleolithic), dogs, and cats, as well as the commensal house mouse, at early Cypro-PPN sites^4,5,13,48^. At Kissonerga-*Mylouthkia*, the presence of the house mouse is considered one of its earliest appearances in its commensal nature outside its initial core zone (the Levant)^49^.

Along the same lines, a Central Anatolian early Neolithic presence on Cyprus is also archaeologically supported, by the occurrence of small amounts of imported obsidian of confirmed Central Anatolian origin, reported at the earliest Cypro-PPNA settlements^51^, increasing considerably over the following centuries^50,52^, also attested at Cypro-PPNB Kissonerga-*Mylouthkia*^47^. These archaeological findings have been interpreted as maritime contacts between Cyprus and Anatolia during the early Neolithic, as part of a presumed exchange network^9,50,52^.

The available archaeological evidence and the current genetic findings, therefore, support a scenario where a group of Central Anatolian farmers possibly exploited pre-existing maritime connections, involving obsidian transport^52^ (Supplementary Information, Sect. 6) and settled the island of Cyprus, prior to the excess Zagros-related ancestry spread to most populations from their region of origin, as apparent in specific Aceramic Neolithic groups reanalysed in the present study (e.g. Aşıklı Höyük and Musular). Assuming that the admixture event giving rise to Mylouthkia took place on Cyprus, a plausible hypothesis would be that incoming Neolithic Anatolians intermixed with evidently earlier and smaller Levantine groups already present on the island since the Epipaleolithic and/or PPNA (Supplementary Information, Table 4, scenarios 1 and 2). Whether this was an island-wide phenomenon or specific to the sampled Cypro-LPPNB individuals from Mylouthkia in western Cyprus, remains to be determined.

Although we cannot exclude the possibility of an unsampled mainland Near Eastern population with a similar genetic profile to the analysed Cypro-LPPNB migrating directly to Cyprus (Supplementary Information Table 4, scenarios 3 and 4), there is currently limited evidence to support this hypothesis (Supplementary Table S7, Supplementary Table S10). Other potentially plausible single ancestral sources for Cypro-LPPNB could possibly originate from Aceramic Neolithic south-central Anatolia (e.g. Neolithic phase of Pınarbaşı), or from the northern Levant (e.g. Mureybet and Abu Hureyra), but such scenarios (Supplementary Information, Table 4) cannot be currently evaluated due to lack of genetic data.

As a secondary finding of our study, we provide new evidence for high allele sharing between Cypro-LPPNB Mylouthkia and a selected group of the earliest European farmers, who postdate these Neolithic Cypriots. It is very likely that groups deriving from the examined Cypro-LPPNB individuals had the technological knowhow and skills to cross relatively large stretches of open sea^9–12^, maintaining strong maritime connections, which is evident in repeated and systematic seafaring expeditions to and from the mainland, required for maintaining a sustainable stock of animals and valuable material (e.g. obsidian) on the island throughout the Neolithic^9,11,12,52^. Although the genetic similarity between the analysed Cypro-LPPNB and the earliest European Neolithic farmers could be interpreted in context of a presumable maritime spread of agriculture into Europe^53–56^, more parsimonious models involving a direct land migration from Anatolia to southeastern Europe have been suggested^26,43^. With the current lack of further supporting evidence, the high genetic affinity between Mylouthkia and very early European farmers, could be attributed to a shared basal Anatolian population that contributed the majority of ancestry to both Cypro-LPPNB and northwestern Anatolian Ceramic Neolithic groups, who subsequently spread to southeastern Europe^24–26,40,43,57^.

Although informative in providing valuable insights and improving the state of research on the island’s initial settlement and Neolithisation, conclusions on the overall ancestry and genetic affinities of all Neolithic Cypriot farming groups cannot be drawn from the present study. The current findings do, however, provide an important archaeogenetic line of evidence on the most plausible origins of the earliest seafaring settlers on the island of Cyprus, informing long-standing archaeological debates on complex seafaring migrations in the Eastern Mediterranean during the late Pleistocene–early Holocene transition and the early maritime spread of agriculture.

## Methods

### Dating of the Cypro-LPPNB Mylouthkia samples

Our review of archaeological publications resulting from excavations at Kissonerga-*Mylouthkia* established the archaeological context^32^ of the human bone samples that are the focus of this study (I4207/KMY1, I4209/KMYL2, I4210/KMYL3)^28^. All samples were found in a well contained in the site, well 133^28,34^. This context was dated to Cypro-LPPNB with the help of two carbonised seed samples that were extracted from a single deposit (fill 133.264) contained in well 133, for radiocarbon dating. The calibrated date range of these samples is 7500–6700 cal BCE (confidence intervals of calibrated ^14^C dates from well 133 at 95% confidence level, based on OxCal 4.4^58^, using atmospheric curve IntCal20)^59^. No other deposit from within well 133 was sampled for radiocarbon dating.

Two ‘concentrations’ of disarticulated human remains were found in well 133. One occurred in its two uppermost deposits, fill 133.260 (fragmented skull) and fill 133.264 (tooth). The other ‘concentration’ of human bone fragments occurred in fill 133.282 and at least three further deposits below this fill (skulls and human bones)^34^. The samples relevant to our study (providing usable aDNA)^28^ are two skull fragments (samples I4207/KMY1, I4209/KMYL2) from fill 133.260 (upper ’concentration’) and a petrous bone (sample I4210/KMYL3) from fill 133.282 (lower ’concentration’).

As only one deposit from within well 133 was sampled for radiocarbon dating, the well's depositional history has to be reconstructed through stratigraphy and relative dating methods. This has implications for the date of the human bone samples from well 133. Samples I4207/KMY1 and I4209/KMYL2 from fill 133.260 are likely to have been deposited more recently than the deposit that was sampled for radiocarbon dating (calibrated date range of c. 7500–6700 cal BCE, at 95% confidence level). Sample I4210/KMYL3 was probably deposited earlier than the deposit sampled for radiocarbon dating. A Cypro-LPPNB date is likely for all samples as individual fills of well 133 seem to have been deposited in relatively quick temporal succession. It cannot, however, be ascertained how long each depositional phase lasted and how much time passed in-between them.

We use a Cyprus-wide period date of 7600–6800 cal BCE (derived from a range of ^14^C dates from different Cypro-LPPNB sites)^4^ for dating the human bone samples from Kissonerga-*Mylouthkia,* the focus of this study. This is preferable to using the date range of the seed samples extracted from well 133, as their exact stratigraphic relationship to the human bone samples from well 133 remains uncertain. Details of our dating rationale can be found in Supplementary Information (Sect. 1).

### Ancient sample selection and compilation of the archaeogenetic dataset

We used previously published publicly available data from the literature. The three Cypro-LPPNB genomes, along with a selection of other ancient samples of interest (see further below for specific inclusion criteria for sample selection), were retrieved from the working dataset of the original publication^28^ via the Harvard Dataverse repository (https://dataverse.harvard.edu/), in eigenstrat format.

Additional Neolithic samples of interest not included in the above dataset, were retrieved from the comprehensive Allen Ancient DNA Resource (AADR), v 54.1 (10.7910/DVN/FFIDCW)^60^, in eigenstrat format. Additional genomes not included in the latest version of AADR were retrieved, as trimmed BAM files of aligned reads, from the European Nucleotide Archive (ENA) (https://www.ebi.ac.uk/ena/browser/home)^61^. For these samples only (available as BAM files), we derived genotype data by first using Samtools (v1.16) *mpileup* (http://www.htslib.org/doc/samtools-mpileup.html)^62^ to create pileup files of BAMs of ds libraries and extracted SNP data from the generated pileup files using *pileupCaller* from the SequenceTools software package (v1.5.2) (https://github.com/stschiff/sequenceTools/tree/master/src/SequenceTools), in eigenstrat format. The resulting datasets were merged using the tool *mergeit* from the EIGENSOFT (v.6.01) package (https://github.com/argriffing/eigensoft/tree/master/CONVERTF)^42,63^.

The analysed samples were chosen based on their relevance to the examined Cypro-LPPNB population, geographically (Levant, Mesopotamia, Zagros, Anatolia, southeastern Europe) and chronologically (the Epipaleolithic/Mesolithic, Pre-Pottery Neolithic, early Pottery Neolithic, and the initial European Neolithic).

Following the initial selection, the following exclusion criteria were applied: (i) samples labelled as ‘contaminated’ in the source curated datasets; (ii) samples with very low coverage (< 0.01X); (iii) samples labelled as ‘outliers’ in the source curated datasets or apparent from our initial principal components analysis (PCA) (further details below); (iv) Early European Neolithic samples dated after ~ 5500 BCE or with excess European hunter-gatherer (HG) admixture (including thus the earliest farmers of Europe, labelled Very Early European Farmers—VEEF).

Our main samples of interest span a period of ∼7,000 years (c. 14,000–7000 cal BCE) and can be subdivided into the following metapopulations: (i) Epipaleolithic/Mesolithic populations from the Levant^17^, Anatolia^18^, the Zagros^17^, the Caucasus^37^, Southeast Europe^39^, and North Africa^38^; (ii) Pre-Pottery Neolithic populations from the Levant^17,18,21^, Anatolia^18,19,29^, Upper Mesopotamia^21,22,28^, Northwest Zagros^28^, Central Zagros^17,23^, and Cyprus^28^; (iii) Pottery Neolithic populations from Anatolia^19,25,28,30,36,40^; and (iv) the earliest Neolithic individuals from Europe^26,28,30,39,41,64^. All specific populations included in our working dataset can be found in Table 1, as well as in Detailed Methods (Supplementary Information, Sect. 8) and Supplementary Table S1.

The three genomes from Cypro-LPPNB Mylouthkia, the main focus of the present study, are of low coverage (0.031–0.046X)^28^, yet sufficiently usable for genomic analysis^45^.

### Population structure and overall genetic variation

We merged our aDNA dataset with 61 publicly available modern West Eurasian populations^17^ from the AADR database (~ 500,000 SNPs)^60^, using tool *mergeit* from the EIGENSOFT (v.6.01) package (https://github.com/argriffing/eigensoft/tree/master/CONVERTF)^42,63^. We initially computed a principal components analysis (PCA) for determining population structure on the 61 Human Origins modern West Eurasians and projected the ancient individuals included in our working dataset, using the *smartpca* tool from the EIGENSOFT (v.6.01) package (https://github.com/chrchang/eigensoft/blob/master/POPGEN/smartpca.info)^42,63^. The first two principal components (PCs) were plotted creating a 2-dimensional plot.

This procedure was initially followed for identifying outliers and any other ‘anomalies’ in the expected genetic variation and a second time for deriving the final PCA plot, after making any necessary exclusions (see criteria above). The final PCA plot includes 166 ancient individuals, achieving an ideal population structure among the different groups and revealing an unbiased genetic variation for the working dataset (Fig. 2).

### Genetic affinity via shared genetic drift

We used the package ADMIXTOOLS v.2.0.0^47^ in R (version 4.1.1)^65^, operated using RStudio (v. 2022.07.1 + 554)^66^, to estimate outgroup *f3-*statistics of the form *f3(outgroup; population A, population B)*, where in our case, outgroup = Mbuti, population A = Cypro-LPPNB, and population B = all ancient populations in our working dataset, in turn.

Outgroup *f3-*statistics calculate allele frequency correlations between pairs of populations (Cypro-LPPNB vs other ancient population, in this case) to measure shared genetic drift between them from an outgroup population, determining thus pairwise genetic affinity via allele sharing^48^. The higher the value of the *f3-statistic* the higher is the genetic affinity between Cypro-LPPNB and the tested ancient population, in turn.

Outgroup *f3-*statistics of the form *f3*(Mbuti; pop1, pop2) were also estimated for relevant Epipaleolithic and all Neolithic populations in our comparative dataset in order to provide an overall picture of genetic affinities between tested populations, Based on these, we constructed a dissimilarity matrix comprising pairwise distances of the form 1 minus *f3*(Mbuti; pop1, pop2)^67^ (Supplementary Table S6) and performed Multidimensional Scaling (MDS) analysis using the *cmdscale* function in R 4.1.1 (goodness of fit: R^2^ = 0.74, F = 2074, p < 0.001). We then constructed an MDS plot displaying genetic affinities between the tested populations in a two-dimensional panel (Fig. 4c and more extensive version in Supplementary Fig. S3).

In order to further investigate genetic distances between populations, in comparative terms, we estimated *f4-*statistics of the form *f4(outgroup, population A; population B, population C)*. This analysis was used for answering two different questions: (a) Which Near Eastern population groups predating Cypro-LPPNB share more alleles with them, in comparative terms? (i.e. identification of potential ancestral sources); (b) Do Cypro-LPPNB or Neolithic Anatolians share more alleles with the earliest European Neolithic farmers?

For the first purpose, outgroup = Mbuti, population A = Cypro-LPPNB, population B = first comparison ancient Near Eastern population, and population C = second comparison ancient Near Eastern population. In this case, we followed a systematic approach by first comparing meta-populations (Anatolia, Levant, Zagros/Mesopotamia) as merged groups to identify the region from which genomes show the highest genetic proximity to Cypro-LPPNB. We then repeated the analysis comparing population groups within these regions, to identify specific proximal ancestral sources.

For the second purpose, outgroup = Mbuti, population A = VEEF population groups, population B = Cypro-LPPNB, and population C = Anatolian PPN and PN populations, in turn. As in the case of *f3*-statistics, *f4*-statistics are based on correlations of allele frequency differences, thus estimating shared genetic drift, but involving four different populations instead of three. For example, negative *f4* values indicate increased gene flow between VEEF and Cypro-LPPNB (relative to Neolithic Anatolians) and positive *f4* values indicate increased gene flow between VEEF and Neolithic Anatolians (relative to Cypro-LPPNB).

The strength of *f-*statistics is that they are robust to divergence time, small population sizes, missing data, and lower data quality, affecting any of the tested populations^45,47^. This is a relevant and particularly important for the present study strength of the tool, given the low coverage of the Cypro-LPPNB samples. For all relevant *f*-statistics, standard errors were estimated by 5 cM block jack-knifing. Further details on *f-*statistics in Detailed Methods, Supplementary Information (Sect. 8).

### Estimating admixture proportions

We used *qpAdm*^45^ from ADMIXTOOLS v.2.0.0^47^ in R (version 4.1.1)^65^, in order to estimate admixture proportions for Cypro-LPPNB Mylouthkia from a list of Near Eastern source populations predating our target population. In order to run such a model, *qpAdm* additionally requires a list of reference populations, where at least some of these are more closely related to given source populations than to others^45^. We followed a recently implemented methodology, termed ‘rotating models’^45^, which requires a single set of populations (i.e. not separated into source and reference), from which sources are sequentially selected, treating the remaining as references. With this approach, each defined population is treated both as a source and as a reference (in subsequent models), with the tool ‘blindly’ (i.e. not relying on prior hypotheses of the researcher) determining the composition of admixture models, utilizing all possible combinations of defined sources. This approach has been found to generate consistent results on a common set of principles, rendering findings more robust and directly comparable, in comparison with the default ‘base’ model approach^45^.

Despite our target population comprising a small number (n = 3) of low-coverage samples, specific strengths of *qpAdm*, allow the estimation of relatively accurate and unbiased admixture proportions^42^. In particular, recent simulation data indicate that *qpAdm* provides robust ancestry estimates in cases where the target, source, or reference populations have small sample sizes, as well as in cases where one or more populations in the model has a high rate of missing data^42^. In our analysis, we used *qpAdm* for two purposes: (i) to determine the distal (deep) ancestry of all Neolithic populations included in our dataset; and (ii) to determine the proximal ancestry of the Cypro-LPPNB Mylouthkia group.

For the first purpose, we used the following list of populations: TUR_Pinarbasi_EpiP, ISR_Natufian_EpiP, IRN_C_Zagros_Mes_N, EHG, SRB_Iron_Gates_HG, CHG, MAR_Taforalt_EpiP, WHG, RUS_AfontovaGora3, RUS_MA1_HG, Mbuti.DG, with the latter four fixed in the reference list (i.e. used only as references and not as sources). The choice of references is based on previous analyses on similar research hypotheses in the literature, to aid consistency^20,28^. The list of targets comprised all Near Eastern and European Neolithic populations of interest (Table 1). It should be noted that population group ‘IRN_C_Zagros_Mes_N’ meant to represent Mesolithic Central Zagros, is composed of a relatively low coverage Mesolithic sample from Hotu IIIb (Central Zagros) and an early Neolithic sample from Tepe Abdul Hosein (Central Zagros)^17^, positioned very closely to the aforementioned Mesolithic individual in our PCA plot. Combination of these two samples in a single source improves precision of admixture estimates in our distal model, without compromising validity. Further details on this, in Detailed Methods (Supplementary Information, Sect. 8).

In order to determine relative excess basal Levantine (ISR_Natufian_EpiP) versus basal Central Zagros (IRN_C_Zagros_Mes_N) ancestry, among Pre-Pottery Neolithic populations contemporaneous to Cypro-LPPNB Mylouthkia, the natural logarithm of the ratio of the distal admixture proportion of the corresponding components (proportion ISR_Natufian_EpiP / proportion IRN_C_Zagros_Mes_N) was plotted using R (version 4.1.1)^65^.

For the second purpose, determining the proximal admixture in Cypro-LPPNB Mylouthkia using *qpAdm*, our choice of source populations was based on the relevant literature^17,20,28^, but also on findings from our distal model, which revealed the deep ancestral composition of both the target Cypro-LPPNB and all potential source populations (Supplementary Table S3). Our sources included the following potentially ancestral population groups predating the target Cypro-LPPNB samples (7600–6800 cal BCE): (i) an Epipaleolithic Anatolian source (TUR_Pinarbasi_EpiP); (ii) PPN Anatolian sources (TUR_C_Boncuklu_PPN, TUR_C_AsikliHoyuk_PPN, TUR_C_Musular_PPN); (iii) Epipaleolithic/PPN Levantine sources (ISR_Natufian_EpiP, ISR_Motza_PPNB, ISR_KfarHaHoresh_PPNB, JOR_AinGhazal_PPNB, JOR_Baja_PPNB); and (iv) PPN Mesopotamian/Zagros sources (TUR_SE_NevaliCori_PPNB, TUR_SE_Cayonu_PPNB, TUR_SE_NevaliCori_PPNB, TUR_SE_Mardin_PPNA, IRQ_Nemrik9_PPNA, IRQ_Shanidar_PPNA).

Due to the high number of closely related source populations, subsequent models were run, each including a combination of populations from each set (e.g. TUR_Pinarbasi_EpiP, TUR_C_Boncuklu_PPN, ISR_Motza_PPNB, IRQ_Nemrik9_PPNA), until all plausible combinations of sources were examined. A set list of reference populations for all models was chosen, so that these were differentially related to the sources, as required^45^. Further details on *qpAdm* and the choice of source and reference populations in Detailed Methods, Supplementary Information (Sect. 8).

In order to aid consistency with previous analyses on these samples^20^, we additionally ran secondary proximal admixture models following the exact above methodology, this time including Pottery Neolithic population groups not included in our initial source list, that is northwestern Anatolian Aktopraklik (c. 6700–6500 BCE), Barcin (c. 6500–5900 BCE), and Central Anatolian Çatalhöyük (c. 7100–6000 BCE). These population groups were excluded from our main analyses, as they postdate the analysed Cypro-LPPNB samples (c. 7600–6800 BCE). It should be stressed that this analysis was conducted for exploratory purposes and the chronological gap between the two population groups does not allow for any conclusions regarding ancestry of Mylouthkia from these PN groups.

From all models estimated following the above approach, we deemed models as implausible based on recent guidelines^45^ when: (i) the estimated admixture proportions for the target population fell outside the biologically relevant range (0–1), (ii) the model was rejected statistically with a p-value < 0.05, or (iii) the precision was low, as indicated by large estimate standard errors. All relevant proximal ancestry models including potentially ancestral to Cypro-LPPNB Near Eastern population groups, can be found in Supplementary Table S7 indicating clearly which models have a good fit, as well as which are the best fitting models and which are clearly statistically rejected.

### Fitting admixture graphs

We used *findGraphs* and *qpGraph* from ADMIXTOOLS v.2.0.0^47^ in R (version 4.1.1)^65^, in order to fit admixture graphs involving Cypro-LPPNB. These tools utilise estimated *f*-statistics and relevant genetic parameters (drift and admixture weights) to derive graphs representing admixture dynamics and genetic drift for a given list of populations, based on a manually set graph topology (*qpGraph*)^48,49^, or a series of randomly set graph topologies and repeated itemisations for improving the admixture graph, in an automated approach (*findGraphs*)^47^. Model fit is evaluated based on the minimal difference between fitted and estimated *f3*-statistics as a log-likelihood (LL) score, as well as fitted and estimated *f4*-statistics as the worst residual z-score (WR score). A good fitting admixture graph should have an LL score and a WR score close to zero. Further details on the specific tools can be found in Detailed Methods (Supplementary Information, Sect. 8).

For our main analysis, we followed the automated approach for construction of admixture graphs involving Cypro-LPPNB Mylouthkia, as outlined in a recently published protocol for fitting ancient admixture graphs using *findGraphs*^47^. This is described in detail in Supplementary Information (Detailed Methods).

In summary, we initially derived *f2*-statistics blocks from genotype files on a selection of populations of interest, namely all potential sources for Cypro-LPPNB (Epipaleolithic Natufian, Epipaleolithic Pinarbaşi, PPN Boncuklu Höyük, and Levantine PPNB), as identified in our *qpAdm* analysis (see previous sub-section), as well as other relevant distal sources (e.g. Central Zagros Mesolithic/Neolithic and CHG), treating Mbuti.DG as the outgroup population. Following the protocol by Maier et al.^47^, we carried out an initial scan to identify the ideal number of admixture events (graph class complexity) between the given set of populations (3 to 8 admixture events tested, with 100 algorithm iterations per graph complexity class), checking model fit based on the LL score and the WR score. Following this, we ran *findGraphs* again on the chosen complexity classes (in this case 3–5 admixture events), also setting topological constraints (i.e. ‘demanding’ admixture events for Cypro-LPPNB Mylouthkia, PPN Boncuklu, and Levantine PPNB). All ideally fitting graphs were then inspected manually to evaluate their archaeological/archaeogenetic plausibility and graphs involving implausible admixture dynamics (e.g. Neolithic populations appearing as admixture sources of Epipaleolithic/Mesolithic populations rather than vice versa) were rejected.

The final admixture graphs were then compared to identify common features, particularly as regards the admixture event giving rise to Cypro-LPPNB. According to Maier et al.^47^, among a set of ideally fitting admixture graphs (based on LL score and WR score), the graphs with the minimal number of admixture events should be preferred.

In addition to the above automated approach, *qpGraph* was applied by manually setting the graph topology based on the estimated admixture composition of Cypro-LPPNB and other population groups of interest (see below), as identified using *qpAdm* (previous sub-section). Further details in Supplementary Information (Detailed Methods).

Furthermore, in order to confirm that indeed Boncuklu is the best fitting Anatolian source for Cypro-LPPNB Mylouthkia (as revealed in *qpAdm* analysis), we repeated the exact same automated procedure using *findGraphs*, including Aşıklı Höyük in the place of Boncuklu, comparing the model fit and topology plausibility of the best fitting graphs, to those of the initially derived admixture graphs.

Finally, in order to confirm the specificity of the graph topology and in particular the inferred admixture events that gave rise to Cypro-LPPNB Mylouthkia, as revealed in the best fitting admixture graphs derived from our automated *findGraphs* analysis, we applied the exact same approach including, in turn, Çatalhöyük and Barcin Höyük in the set of populations in the place Mylouthkia.

### Inferring the timing of admixture events

To provide further insight into the timing of the admixture event that gave rise to Cypro-LPPNB, we applied the Distribution of Ancestry Tracts of Evolutionary Signals (*DATES*) tool^50^ (see Supplementary Information, Detailed Methods for further details). *DATES* estimates the time of a given admixture event based on the weighted ancestry covariance across the genome, where two reference populations represent the potential ancestral sources of a target population^50^. *DATES* does not require phased data and can be effectively used with pseudo-haploid (instead of diploid) genotype calls, remaining robust despite small sample sizes and high numbers of missing SNPs, making it suitable for ancient DNA analysis^50^. *DATES* returns inferred admixture timing in generations prior to the date the target samples have lived, converted to years by assuming a mean generation to last 28 years^68^ and further into absolute dates (e.g. years BCE) by adding the sampling age of the target ancient genomes involved.

We applied *DATES* to infer the timing of the admixture event for the four best fitting two-way admixture models, as identified in *qpAdm* (see sub-section above). A normalized root-mean-square deviation (NRMSD) of < 0.7 in combination with a Z-score of > 2, given the time of admixture (λ) is < 200 generations, provide an indication of good model fit^50^. Since the developers of the method propose that these criteria should not be applied strictly, they were only used as a rough indication of successful estimation of admixture timing and not as a definite cut-off for accepting or rejecting admixture timing inference.

In order to determine whether the inferred admixture timing for Cypro-LPPNB Mylouthkia also characterises other roughly contemporaneous groups from the surrounding mainland, or whether it is unique and specific to the former population group, we used *DATES* to infer the admixture timing for relevant mainland Near Eastern population groups. As these groups are potentially involved in admixture dynamics giving rise to Cypro-LPPNB Mylouthkia, these analyses serve to create a more comprehensive picture of admixture timings in the region surrounding Neolithic Cyprus, providing a plausible timeframe, within which the Mylouthkia group emerged. Further details in Supplementary Information (Detailed Methods).

### Kinship analysis

To infer family relationships for the three Cypro-LPPNB Kissonerga-*Mylouthkia* samples of interest (I4207, I4209, I4210), we applied the tool READ^35^ using default parameters. READ estimates the coefficient of relatedness between two genomes (pseudo-haploid data) based on the proportion of non-matching alleles between them (P0), normalized with the pairwise allele differences among unrelated individuals within the population (α), calculated as the median from the working dataset, thus correcting for within population diversity, SNP ascertainment, and marker density.

Based on the normalised proportion of shared alleles, READ infers kinship for each pair of tested samples as: ‘identical’ (i.e. two samples from same individual or identical twins); ‘second-degree relatives’ (i.e. nephew/niece-uncle/aunt, grandparent-grandchild or half-siblings); ‘first-degree relatives’ (parent–offspring or siblings); or ‘unrelated’. The accuracy of the technique has been previously evaluated and found to provide consistent kinship inferences at low genome coverages (0.1–0.5X)^35,69^. READ also provides the uncertainties for a given kinship estimation, namely the distance to the classification cutoffs, expressed as multiples of the standard error of the mean (Z).

### Visualisations

For all visualisations we used R (version 4.1.1)^65^, operated using RStudio (v. 2022.07.1 + 554)^66^. All plots were created using package *ggplot2*. For creating vertical stacked barplots (e.g. for presenting admixture proportions), we additionally used package *forcats.* For creating line plots (e.g. chronology plot) and for PCA plots, we additionally used package *tidyverse*. For all plots involving maps (e.g. pie charts on map, symbols on map), we additionally used package *rworldmap*. For combining plots, we used packages *patchwork* and *cowplot*, and where necessary Adobe Illustrator v. 27.4.

### Supplementary Information

Supplementary Information.

Supplementary Tables.

## Data Availability

All genomes analysed in the present study are freely available in the following publicly available repositories: (1) Harvard Dataverse repository (https://dataverse.harvard.edu/); (2) Allen Ancient DNA Resource (10.7910/DVN/FFIDCW); (3) European Nucleotide Archive (https://www.ebi.ac.uk/ena/browser/home). All ancient individual samples used in the analyses of the present study can be found in Supplementary Table S1, while population characteristics can be found in Supplementary Table S2. All steps and software used for the compilation of the working dataset and for conducting all formal analyses, can be found in Detailed Methods (Supplementary Information, Sect. 8). All data used for creating the main and supplementary figures, can be found in Supplementary Tables S3–S14.
