# Supplementary material for: Palaeogenomic insights into the origins of early settlers on the island of Cyprus
Source: Sci Rep. 2024 Apr 26;14:9632. doi: 10.1038/s41598-024-60161-z (PMC11053055; doi:10.1038/s41598-024-60161-z)
Supplement: Supplementary file 1 — Supplementary Information. [file 41598_2024_60161_MOESM1_ESM.docx]

**Supplementary Information**

**Table of contents**

1. Archaeological context and dating of human samples from Kissonerga-*Mylouthkia* used in the present study.……………………………………………………………………..……………..…3

2. Overview of the archaeogenetic context during the late Pleistocene–early Holocene in regions surrounding Cyprus…..…….………………………………………………..……14

3. Literature overview on uniparental marker ancestry among Cypro-LPPNB Mylouthkia and surrounding population groups……………………………………………..……..20

4. Overview of the evidence on the plausibility of different admixture scenarios for Cypro-LPPNB Mylouthkia, as revealed in the present study.……………………………..……21

5. Overview of archaeological evidence on the origins of the earliest seafarers reaching Cyprus……….………………………..………………….…....………………....................…….24

6. Overview of archaeological evidence on the maritime connections between Anatolia and Cyprus during PPNB.…………………………..……………………………………………..27

7. List of Supplementary Tables and Figures…………………………..………………………..…….28

8. Detailed Methods……………………………………………………………………………………………….31

9. References………………………………………………..……………………………………..………………..44

10. Supplementary Figures…………………………………………………………….…….………………..52

**Palaeogenomic insights into the origins of early settlers on the island of Cyprus**

Authors:

Alexandros Heraclides^1^*, Aris Aristodemou^2^, Andrea N. Georgiou^1,3^, Marios Antoniou^4^, Elisabeth Ilgner^5^, Leonidas-Romanos Davranoglou^6^

Affiliations:

^1^ School of Sciences, European University Cyprus, Nicosia, Cyprus.

current address: European University Cyprus, 6 Diogenis Str., 2404 Engomi, P.O. Box: 22006, 1516 Nicosia, Cyprus

^2^ Department of Infectious Disease, Faculty of Medicine, Imperial College London, London, UK

^3^ Department of Hygiene and Epidemiology, University of Ioannina, Ioannina, Greece

^4^ Department of Electrical and Computer Engineering, University of Thessaly, Volos, Greece

^5^ School of Archaeology/Merton College, University of Oxford, Oxford, UK

^6^ Oxford University Museum of Natural History, University of Oxford, Oxford, UK

* corresponding author

[a.heraclides@euc.ac.cy](mailto:a.heraclides@euc.ac.cy)

**1. Archaeological context and dating of human samples from Kissonerga-*Mylouthkia* used in the present study**

Overview of Cypriot chronology during the Epipaleolithic and Pre-Pottery Neolithic

The earliest evidence of material culture on the island of Cyprus at the terminal Pleistocene comes from the collapsed rockshelter site Akrotiri-*Aetokremnos* in southern Cyprus (11,000–9000 cal BCE, corresponding to the Epipaleolithic era)^1^. The cultural horizon of the Akrotiri phase was followed by organised small-scale Pre-Pottery Neolithic A (PPNA/Initial Aceramic Neolithic) villages of farmer-foragers^2-4^, which then developed into organised Neolithic settlements^5^ during the Pre-Pottery Neolithic B (PPNΒ) such as Kissonerga-*Mylouthkia*^6^, the site providing genetic samples for the current study.

Knapp^7^ presents the currently authoritative scheme for the chronology of early prehistoric Cyprus. It combines previous studies’ periodisations with Manning’s^8^ chronological scheme based on Bayesian modelling of calibrated radiocarbon dates from archaeological contexts on Cyprus. While Knapp’s scheme sub-divides Cypro-PPNB (Early Aceramic Neolithic) into Cypriot Early, Middle, and Late PPNB (Supplementary Information Table 1), Manning’s scheme only defines the beginning and end of Cypro-PPNB (c. 8500/8400–6900/6800 cal BCE). Cypro-LPPNB (EAN 3) –– the sub-period of Cypro-PPNB that is the focus of the present study –– seems partly defined by archaeological studies prior to Knapp^7^, providing the date range 7600–7000 cal BCE, partly by Manning’s modelling scheme, providing the period’s alternative end date, 6800 cal BCE (Supplementary Information Table 1)^8^. Knapp’s and Manning’s chronological schemes both use date ranges calibrated with the IntCal09 atmospheric curve^9^ .

| Periods | Chronology | Important sites |
| --- | --- | --- |
| Late Epipalaeolithic | c. 11,000–9000 cal BCE | Akrotiri-*Aetokremnos*^1^  Vretsia-*Roudias*^10^  Akamas-*Aspros*^11^ (probable)^12^ |
| Cypro-PPNA (Initial Aceramic Neolithic) | c. 9000–8500/8400 cal BCE | Ayios Tychonas-*Klimonas*^2^  Ayia Varvara-*Asprokremnos*^3^ |
| Cypro-PPNB (Early Aceramic Neolithic - EAN) | c. 8500/8400–6800 cal BCE | Kissonerga-*Mylouthkia*^6^ Parekklisha-*Shillourokambos*^5^  Kalavasos-*Tenta*^13^  Akanthou-*Arkosyko*^14^  Kritou Marottou-*Ais Giorkis*^15^ |
| Cypro-EPPNB (EAN 1) | c. 8500/8400–7900 cal BCE |  |
| Cypro-MPPNB (EAN 2) | c. 7900–7600 cal BCE |  |
| Cypro-LPPNB (EAN 3) | c. 7600–7000/6800 cal BCE |  |

Supplementary Information Table 1. Overview of Late Pleistocene – Early Holocene Cypriot chronology and archaeological phases from the Epipaleolithic to the early Neolithic (from Knapp^16^, Table 2), including important sites mentioned in this Supplementary Information.

Kissonerga-*Mylouthkia* and the excavated wells

Kissonerga-*Mylouthkia* is a coastal site in western Cyprus, about 5 km north of the modern city of Paphos. Excavations at Kissonerga-*Mylouthkia* (1976–1996) were part of a multi-site project, focussing on pre-Bronze Age settlement patterns in western Cyprus (Lemba Archaeological Project)^6^. Evidence for the site’s occupation dates to Cypro-Pre-Pottery Neolithic B (Cypro-PPNB) and the Early/Middle Chalcolithic. In addition, surface finds attest to human activity at Kissonerga-*Mylouthkia* between the Bronze Age and the Medieval period^17^.

The third research phase at the site (1994–1996) recovered two ‘aceramic’ features that were dated to Pre-Pottery Neolithic B with the help of radiocarbon (^14^C) dating ^17^. These features were two wells: well 116 (Cypro-EPPNB) and well 133 (Cypro-LPPNB). A Neolithic settlement in the vicinity of the wells was hypothesised but not recovered^18, 19^. Cypro-PPNB Kissonerga-*Mylouthkia* was probably visited for specific activities, such as water acquisition from the wells (claimed to be the oldest Neolithic wells^20^), stone working (substantial remains of chipped stone and fragmented stone vessels were found inside the wells), and the acquisition of marine resources for consumption (suggested by molluscs in the wells and human remains displaying external auditory extoses)^21^. The archaeobotanical record, comprising crops and weeds suitable for cultivation, as well as the commensal house mouse (*Mus musculus domesticus*)^22^, supports the hypothesis of a permanent occupation site nearby^19^. The presence of the house mouse at the site is one of the earliest appearances of its commensal nature outside its initial core zone (the Levant). The chipped stone assemblage of the wells was suggested to show affinities with Levantine PPNA/PPNB stone assemblages, while for the recovered obsidian a Central Anatolian source was identified^23^.

The wells (116 and 133) are of about 8–9 m depth and located a few minutes walking distance from the present coastline. Their walls display a series of hollows that were interpreted as handholds/footholds, facilitating maintenance of the wells during their use life^18^. Two fills (deposits) were sampled for ^14^C dating from well 116, one fill from well 133 (Supplementary Information Table 2). Objects contained in the same fill are normally chronologically associated with each other, although inbuilt age and potential differences in their relative age at the time of deposition should be taken into consideration. ^14^C dated samples from Kissonerga-*Mylouthkia* were intentionally selected to represent short-lived samples, in this case carbonised seeds^19^. Therefore, differences between ^14^C dates of samples from the same fills in wells 116 and 133 should rather be explained by measurement uncertainties/inaccuracies and laboratory methods than by actual differences in samples’ dates. These factors are apparent for ^14^C dated samples from well 116, where two samples from the same fill (fill 124) and the same 50-litre soil sample (C 482) from within that fill analysed by different laboratories yielded radiocarbon dates with probability ranges that hardly overlap (Supplementary Information Table 2). Error estimates of one standard deviation for ^14^C dates (68% probability of being correct, assuming normal distribution; Supplementary Information Table 2 below: values behind ± of ^14^C dates) also vary between labs^24^.

Due to the lack of ^14^C dates from a series of deposits (designated ‘fills’ in the excavation report) contained in wells 116 and 133, these contexts’ depositional history has to be reconstructed with the help of stratigraphy (study of stratification in an archaeological context, see sub-section ‘Stratigraphy of well 133’ below). ^14^C dating would only have contributed to an understanding of this depositional history if more fills had been sampled. In addition, if more fills had been sampled, the probability range of the ^14^C dates could have been significantly decreased with the help of Bayesian modelling^25^. The initial archaeological report of the site^19^ provides calibrated dates using the IntCal98 calibration curve^26^. Applying the more recent calibration curve IntCal20^27^, does not significantly alter the calibrated ^14^C dates of the site’s original publication (Supplementary Information Table 2). Nevertheless, up-to-date calibration methods should always be used.

| Context | Fill | Lab code | Sample | ^14^C age  BP | Calibrated date (IntCal98, 95% confidence level)  cal BCE | Calibrated date (IntCal20, 95% confidence level)  cal BCE |
| --- | --- | --- | --- | --- | --- | --- |
| Well 116 | 124 | OxA-7460 | C482 *barley* | 9315±60 | 8738–8326 | 8738–8346 |
|  | 123 | AA-33128 | C481 *grain* | 9235±70 | 8629–8287 | 8622–8296 |
|  | 124 | AA-33129 | C482 *grain* | 9110±70 | 8538–8208 | 8548–8227 |
| Well 133 | 264 | OxA-7461 | C531 *Pistacia* | 8185±55 | 7448–7062 | 7447–7056 |
|  | 264 | AA-33130 | C531 *Lolium sp.* | 8025±65 | 7176–6691 | 7136–6693 |

Supplementary Information Table 2. Cypro-PPNB 14C dates from Kissonerga-*Mylouthkia* (after Peltenburg^19^, table 11.1). Uncalibrated ^14^C ages are given in years BP at 68% confidence level, calibrated dates in years cal BCE at 95% confidence level . Calibration using IntCal98^26^ and IntCal20^27^ atmospheric curves based on OxCal 4.4^28^.

Human remains

Well 116 contained remains of at least one human individual of foetal age, well 133 remains of at least five adult and sub-adult human individuals. Human remains from the wells occurred in the form of cranial and post-cranial body parts. One adult male from well 133 displayed signs of a cultural practice known as occipital deformation (artificial head shaping) applied during infancy, previously observed at the later Neolithic site of Khirokitia^20^. A paleopathological lesion (healed *cribra orbitalia* of the frontal orbits) identified in an adult male possibly provides evidence that he suffered from anaemia at some point during his life. In some individuals from well 133, there is also evidence of severe dental disease (e.g. extensive dental caries, severe attrition leading to pulp exposure, periodontal disease, and in vivo loss of teeth)^20^.

Lorentz^21^ identifies external auditory exostoses (EAEs) in the preserved auditory canals of two adult males from well 133. This is interpreted as an indication of repeated maritime aquatic activity, such as extensive swimming and/or diving, for the purpose of fishing, as well as beachcombing for shells and shellfish, which could be used as food, tools, or ornamentation, as well as for ritual purposes. Parallels of such aquatic activities, evidenced by paleopathological EAEs, were reported in the Levant (Atlit-Yam), Central Anatolia (Aşıklı Höyük), southeastern Anatolia/Upper Mesopotamia (Çayönü), and the northwestern Zagros (Shanidar). Only at the first-mentioned site, however, the context is maritime, the other sites being rather suggestive of freshwater activities^21^.

Some cranial fragments exhibit signs of charring as a result of minimal exposure to fire, likely occurring post-mortem^20^. The patterned, disarticulated deposition of human body parts in well 133 suggests secondary burial rites in which certain body parts were chosen for deposition or the inhumation of individuals who were later cleared aside to make room for new depositions^19^. The human remains in well 133 were partly found in association with a macehead (stone object potentially expressing the bearer’s status and power) and a large number of remains of whole animal carcasses (primarily caprines)^29^. According to Peltenburg^19^, the combined assemblages of human and animal remains, as well as the macehead, may be an indication of the sumptuous depletion of food resources during funerary rituals and an instance of a more general early prehistoric Cypriot custom of depositing at least some of the dead in disused well shafts^19^. A parallel exists in form of a communal burial of about 30 individuals in a disused well at the site of Shillourokambos^19^.

Of the identified human remains, usable aDNA was previously extracted from three adult individuals from well 133^30^, which are further analysed in the present study.

| **Sample** | **Archaeological context** | **Skeletal element** | **Genetic sex** | **No. of autosomal SNPs** |
| --- | --- | --- | --- | --- |
| I4207/KMY1 | F133.260A | Skull (petrous bone) | Male | 35303 |
| I4209/KMYL2 | F133.260B | Skull (petrous bone) | Male | 35618 |
| I4210/KMYL3 | F133.282 | Petrous bone | Male | 51892 |

Supplementary Information Table 3. Basic information of genetically analysed samples from well 133 at Kissonerga-*Mylouthkia*, as reported in the original genetic investigation^30^ (further details on these samples and all other samples analysed in the current study can be found in Supplementary Table S1).

Stratigraphy of well 133

Ten different deposits (fills) were recorded during the excavation of well 133 (Supplementary Information Figure 1). It can generally be assumed that lower deposits were created earlier than deposits above them. Fills 333 and 332 are associated with the use life of well 133 as a water source^18^. The deposits above fills 333 and 332 are all associated with the abandonment of the well (fills 320, 331, 334, 282, 279, 278, 264, 260)^19^. Two samples for ^14^C dating were extracted from the same 120-litre soil sample within fill 264 (C 531), towards the top of well 133 (indicated by uncalibrated ^14^C date figures in Supplementary Information Figure 1).

How much time passed in-between the deposition of individual fills cannot be ascertained with certainty. The good preservation state of the walls of both wells 133 and 116, including handholds, suggests that they were filled up fairly soon after their abandonment, and within a fairly short period of time^19^ . In the case of well 133, this is supported by a seemingly continuous ‘concentration of originally whole caprine skeletons’^29^ stretching over c. 4.25 m of the shaft of the well and ranging across fills 260, 264, 278, 279, and 282 (indicated by grey area in Supplementary Information Figure 1). The deposition of articulated carcasses that did not apparently serve subsistence (remains that could be interpreted as food refuse were found only in low numbers in well 133) suggests a depositional context that is not merely linked to rubbish disposal^29^.

Two ‘concentrations’ of disarticulated human remains were found in well 133, one in the uppermost fills, 260 and 264, the other in fill 282 towards the bottom of the well shaft and fills beneath it (indicated by skulls and ‘HB’ legend in Supplementary Information Figure 1). Peltenburg^19^ and Croft^29^ disagree whether these two concentrations represent ‘a single major depositional episode’^19^ or ‘at least two separate major depositional episodes’^29^. Both assume that the deposition of human remains in well 133 should be associated with the deposition of caprine remains, found in high ‘concentration’ between fills 260 and 282.^19, 29^ This, however, does not account for the human remains found in fills below the ‘'concentration’' of former caprine carcasses (fills 329, 331, 332, 334), partly associated with the use life of the well (fill 329), and for ’Skull 1’ found in fill 260 above the ’concentration’ of caprine remains. It should be noted that human remains were observed in the vicinity of well 133, which had been disturbed by erosion and quarrying^18^. This renders it possible that all or some of the human remains in the well are to be associated with funerary activity outside the well and reached their final depositional context subsequently, by redeposition. Peltenburg^19^ suggests that the human remains in well 133 represent ‘intentional interment of secondary burials’ but notes that ’Skull 1’ –– found above the ’concentration‘ of caprine remains –– received differential treatment in burial than other human remains associated with this ’concentration’. How many depositional events, all in all, are represented by the human bone assemblage of well 133 cannot be ascertained.

‘Skull 1’, providing two samples of recently published aDNA^30^, reanalysed in the present study, represents the disarticulated remains of one or more human head(s) and a partial vertebra, contained in fill 260^20^. Our analysis using READ (Detailed Methods section 8.iii below) suggests that the two aDNA samples extracted from ’Skull 1’ do not represent close (2nd degree or closer) relatives and therefore do not derive from the same individual (Supplementary Table 8). A tooth found in the lower fill 264 was also associated with ‘Skull 1’ as it purportedly fit into a maxilla from fill 260^20^. On stratigraphic grounds, the association between ‘Skull 1‘ and the tooth is indeed possible –– if it is assumed that the skull was dislocated from its original depositional context (fill 264) by later depositional action (resulting in fill 260) –– but it cannot be proved. On the contrary, it is also possible that the tooth moved to the lower layer by bioturbation after deposition.

Fill 282, providing the third sample of recently published aDNA^30^, reanalysed in the present study, contained the remains of at least four individuals, including three skulls (‘Skulls 2–4‘)^20^. The skulls display a fairly structured depositional pattern, one skull (‘Skull 4’) being placed towards the middle of the well shaft, the other two towards the sides and spatially associated with a stone macehead^29, 31^. The sample code of the extracted aDNA does not indicate which of the minimum of four individuals associated with fill 282 is represented by the sample.

The relevance of ^14^C dated samples from fill 264 for parts of ‘'Skull 1’ found in fill 260 (providing two samples of recently published aDNA^30^ reanalysed in the present study), further above, can be argued for on archaeological grounds, based on the association of these human remains with a tooth from fill 264. This, however, might be doubted as the analysed parts of ’'Skull 1’ previously believed to have derived from the same individual are now known to derive from two individuals.

The relevance of ^14^C dated samples from fill 264 for human remains found in fill 282 (providing one sample of recently published aDNA^30^ reanalysed in the present study), c. 3 m further below, can be argued for on archaeological grounds, based on the association of these human remains with the continuous multi-fill ‘concentration’ of former carcasses.

The relevance of ^14^C dated samples from fill 264 for all human remains providing aDNA samples can be argued for on archaeological grounds, based on:

- the apparently short-lived depositional history of well 133, as described above;
- the structured depositional context conveyed by the human and animal bone assemblages between fills 260 and 282, not indicative of rubbish disposal, as noted above.

On grounds of inference, however, it might be questioned whether the pair of ^14^C dates from a single soil sample in fill 264 is relevant for dating the multi-fill ‘concentration’ of carcasses (logical fallacy: composition). Conversely, if an overall date is assumed for the multi-fill ‘concentration’ of carcasses, its application to individual objects contained therein is also a logical fallacy (division). In archaeological terms, these inferential problems are conveyed by the issues of inbuilt and relative age of objects at the time of deposition. In the case of well 133, these issues particularly concern the question of the number of depositional events and the identification of individuals represented by the human bone assemblage of well 133.

The relative age of the deposited human remains could have been better assessed if their nitrogen, fluorine, and uranium contents had been measured (viable for finds from the same deposit, here: fill 282)^32^.

Supplementary Information Figure 1. Schematic representation of the section of well 133, showing concentrations of human remains and location of ^14^C date samples in relation to fills (numbered). Adapted from ^31^, fig. 1, to include information contained in ^6^, fig. 29 (fills/deposits, descriptions of well morphology/site context).

Dating human bone samples from well 133

The recent publication of aDNA samples from PPNB Kissonerga-*Mylouthkia,* which are the main focus of the present paper, dates these samples to 8300–7000 BCE, based on their archaeological context^30^. We propose a different date range for these samples, namely 7600–6800 BCE, based on a critical review of the samples’ archaeological context and previous indirect dating methods applied to them.

The location of the three recently published aDNA samples, which are the main focus of the present paper, within well 133, can be inferred from their published sample codes^30^. Samples I4207/KMY1 and I4209/KMYL2 –– with full sample codes ‘KMY1; KMYL 1181, F133.260A, Skull1' and 'KMYL2; KMYL 1181 F133.260 B, Skull1, !2!' –– both seem to derive from fill 260 of well 133 (Supplementary Information Table 3). In addition, both samples are associated with ‘Skull 1’ –– the disarticulated remains of one or more human head(s) and a partial vertebra (see subsection 'Human remains’ above) –– contained in fill 260. Sample I4210/KMYL3 –– with full sample code ‘KMYL3; KMYL F133,282 !3!’ –– derives from fill 282 in well 133 (Supplementary Information Table 3). From the sample code it is not clear which of the minimum number of four individuals contained in fill 282^20^ this sample should be associated with.

The original publication of well 133 dates this context to Cypro-LPPNB based on two ^14^C dated samples extracted from fill 264 (see subsection Stratigraphy of well 133 above). It interprets the confidence intervals of the extracted samples’ calibrated ^14^C dates as `occupation phase 1B’ of Kissonerga-*Mylouthkia*, ranging between c. 7200 and 6800 cal BCE^17^. Since these samples should, however, be assumed to represent an event –– in this case, the harvest of the seeds sampled for ^14^C dating –– rather than a period of time, it is not possible to interpret their calibrated dates’ confidence intervals as a cultural phase per se. The overall series of ^14^C date samples from Cypro-PPNB contexts at Kissonerga-*Mylouthkia* (see subsection Kissonerga-*Mylouthkia* and the excavated wells above) is small and dates within this series represent a maximum of three events in archaeological terms (three archaeological contexts, here: fills) that have so far not been statistically modelled as ’'occupation phases’'. On formal grounds, therefore, we refrain from interpreting the confidence intervals of the ^14^C date samples from Cypro-PPNB contexts at Kissonerga-*Mylouthkia* as ’'occupation phases’'. We note, however, that the confidence intervals (at 95% confidence level) of the ^14^C date samples from well 133 at Kissonerga-*Mylouthkia* (c. 7500–6700 cal BCE, see Supplementary Information Table 2) roughly correspond to the Cyprus-wide LPPNB period date of c. 7600–7000/6800 BCE defined by Knapp (Supplementary Information Table 1)^16^. This makes it likely that the dated samples indeed date to Cypro-LPPNB. On archaeological grounds, it may be argued that this date is also relevant for the human bone remains contained in well 133 (see subsection Stratigraphy of well 133 above). We refrain from decreasing the overall confidence interval of the two ^14^C dates from well 133 by statistical combination after calibration (OxCal Combine command), since the confidence intervals (at 95% confidence level) of the two calibrated ^14^C dates hardly overlap, resulting in poor fit with the modelled combined date (tested with OxCal 4.4^28^). This could indicate problems with sampling and/or laboratory methods (e.g., sample pre-treatment).

The supplement to the recent publication of aDNA samples from PPNB Kissonerga-*Mylouthkia* does not make reference to the precise find context of these samples within well 133^30^. Instead, the supplement provides a date range for Cypro-PPNB of c. 8200–7200 BCE, a date range for `period 1A’ of occupation at Kissonerga-*Mylouthkia* of c. 8600–8200 cal BCE, and a date range for `period 1B’ of occupation at Kissonerga-*Mylouthkia* of c. 7200–6800 cal BCE. The latter two ranges clearly derive from the original publication of excavations at Kissonerga-*Mylouthkia*, interpreting the confidence intervals of calibrated ^14^C dates from well 116 (`period 1A’) and well 133 (‘period 1B’) as ’occupation phases’ of the site^17^. As argued above, this is not possible on formal grounds. The date range for Cypro-PPNB given in the supplement to the recent publication of aDNA samples from PPNB Kissonerga-Mylouthkia, c. 8200–7200 BCE, seems to derive from a misreading of the maximum range of the two ’occupation phases’ at Kissonerga-*Mylouthkia* (reading inner range 8200–7200 cal BCE, rather than maximum range 8600–6800 cal BCE). The same date range, 8200–7200 BCE, was used by another recent study investigating human remains from well 133 using paleopathological methods^21^.

We argue that the dates assigned to aDNA samples from PPNB Kissonerga-*Mylouthkia* in their recent publication^30^ do not accurately render an indirectly derived, contextual date for the following reasons:

- Date ranges for the samples given in the published dataset (see section 6. Detailed Methods below), 8300–7000 BCE, are claimed to be based on archaeological context. But these date ranges do not correspond to the date ranges for the archaeological context given in the supplement of the same publication, 8200–7200 BCE^30^. The difference between the two ranges cannot at present be explained.
- The date ranges in the supplement derive from a formally flawed interpretation of calibrated ^14^C date confidence intervals as site ‘occupation phases’ from the original publication of excavations at Kissonerga-*Mylouthkia*^17^–– and from a misreading of these ranges.
- The date ranges do not reflect the identity of the genetically analysed samples as deriving from the context of well 133. Instead, they include the irrelevant date ranges of calibrated ^14^C dates from well 116.
- The date ranges do not reflect the location of the genetically analysed samples in relation to the fills of well 133, with potential implications for indirectly dating these samples, based on the stratigraphic relationship between their fills and the fills that were sampled for ^14^C dating (see subsection Stratigraphy of well 133 above).

We consider the following approaches relevant to dating the recently published aDNA samples^30^ from Kissonerga-*Mylouthkia* by their archaeological context:

(1) *Dating by depositional context*. Human remains associated with ‘Skull 1’ from well 133, mainly from fill 260, are likely to have been deposited more recently than ^14^C dated samples from fill 264 (see sub-section Stratigraphy of well 133 above). It cannot be ascertained how much time passed in-between these hypothetically separate depositional events. Remains found in the lower fills of the well (fill 282 and below) are likely to have been deposited earlier than ^14^C dated samples from well 133. Apart from the difficulty of assessing stratigraphic disturbances for human bone samples from the upper fill(s), which could have been removed from their original depositional context and subsequently been redeposited in a later context, a further limitation to this dating method is that no data concerning inbuilt age and relative age differences between different samples are available.

Recommended date if this method is chosen: fixed point for relative dating c. 7500–6700 cal BCE (confidence intervals of calibrated ^14^C dates from well 133 at 95% confidence level, based on OxCal 4.4^28^, using atmospheric curve IntCal20^27^, see Supplementary Information Table 2). 7500–6700 cal BCE is *terminus post quem* for deposition of ‘Skull 1’ (including recently published aDNA samples I4207/KMY1 and I4209/KMYL2, see Supplementary Information Table 3), and *terminus ante quem* for deposition of human remains in fill 282 (including recently published aDNA sample I4210/KMYL3, see Supplementary Information Table 3).

Given the general limitations of this dating method, combining statistical distributions with fixed points in a chronology, and due to the lack of a series of ^14^C dated samples from the archaeological feature (well 133) overall that would allow a full chronological reconstruction of its depositional history , we do not use this dating method for deriving relative dates for the recently published aDNA samples from Kissonerga-*Mylouthkia*.

(2) *Dating by archaeological period*. The pair of ^14^C dated samples from fill 264 within well 133 yielded, approximately, Cypro-LPPNB dates. Using a Cyprus-wide period date (derived from a range of calibrated ^14^C dates from different sites) for dating the specific context of well 133 is preferable to using the date range of the reconstructed Cypro-LPPNB ‘occupation phase 1B’ at Kissonerga-*Mylouthkia* (7200–6800 cal BCE) reported in the original archaeological report^17^, since the latter represents a formally flawed interpretation of confidence intervals associated with ^14^C dates.

Recommended date if this method is chosen: 7600–6800 cal BCE^8, 16^, into which the calibrated dates of ^14^C dated samples from well 133 approximately fall: c. 7500–6700 cal BCE (confidence intervals of calibrated ^14^C dates from well 133 at 95% confidence level, based on OxCal 4.4^28^, using atmospheric curve IntCal20 ^27^, see Supplementary Information Table 2). This date range is slightly different from the date range given for Cypro-LPPNB by Knapp^16^ (c. 7600–7000/6800 BCE, Supplementary Information Table 1). Using 6800 BCE as lower limit is preferable to using the inner limit 7000 BCE as the former lower limit is grounded in Manning's^8^ ^14^C date model-based chronological scheme rather than archaeological studies prior to Knapp^16^ (see subsection Overview of Cypriot chronology during the Epipaleolithic and Pre-Pottery Neolithic above). In addition, the larger range of 7600–6800 BCE incorporates a larger proportion of the confidence intervals (at 95% confidence level) of calibrated ^14^C dates from well 133 at Kissonerga-*Mylouthkia* (c. 7500–6700 cal BCE , see Supplementary Information Table 2).

In the absence of a larger series of ^14^C dates from Kissonerga-*Mylouthkia* or dates obtained directly from the human bone samples that are the focus of the present study, the period date is a last resort. Due to the limitations of dating the recently published aDNA samples from Kissonerga-*Mylouthkia* by relative dating in their depositional context (method (1) above), we date the samples by archaeological period (7600–6800 cal BCE). This is the date quoted in the main manuscript text of the present study.

For potential future studies of aDNA samples from Cypro-PPNB contexts we recommend direct dating as best practice (obtaining ^14^C dates from samples identical to aDNA samples). The archaeological literature sets multiple precedents of intrusive elements in human bone assemblages, resulting in vast chronological gaps within these assemblages that would have remained unidentified if ^14^C dating had not been performed on them^33, 34^. Such precedents demonstrate the importance of direct dating to aDNA studies. Potentially erroneous dates of genetically analysed samples, particularly if they are of relatively low coverage, fundamentally influence the conclusions that can be drawn from aDNA analysis. Applying valid dating procedures is therefore an essential part of aDNA analysis.

**2. Overview of the archaeogenetic context during the late Pleistocene–early Holocene in regions surrounding Cyprus**

In the process of determining the genetic ancestry of one of the oldest insular Mediterranean Neolithic groups at Kissonerga-*Mylouthkia*^6^, we performed a comprehensive phylogenetic re-evaluation of all published Neolithic Near Eastern and the earliest southeastern European population groups, providing important insights into the genetic composition and admixture dynamics of the Pre-Pottery (Aceramic) and the early Pottery (Ceramic) Neolithic of the Fertile Crescent (the regions of the Levant, Upper Mesopotamia, and the Zagros) as well as Central and northwestern Anatolia. Below follows an overview of the archaeogenetic context of these regions (with emphasis on the Levant and Anatolia), during the late Pleistocene – early Holocene, based on the current literature and in light of new findings from the present study.

*Late Pleistocene*

During the late Pleistocene, the archaeological record of the Near East and southeastern Europe, reveals the presence of distinct hunter-gatherer (HG) groups in the Balkans^35^, Anatolia^36^, the Levant^37^, the Zagros^38^, and the Caucasus^39^. Archaeogenetic evidence accumulated over the past 10 years, clearly reveals that, even though these groups were sharing similar cultural practices and customs, they were genetically distinct, forming the following broad groups: (i) Mesolithic Balkan HGs (residing primarily along the Danube, with the archaeogenetics record comprising primarily of individuals from the Iron Gates)^40^, (ii) Central Anatolian HGs (one individual sampled from the site of Pinarbaşi, genetically appearing to share deep ancestry with Balkan HGs but forming a clearly distinct genetic group)^41^, (iii) Levantine HGs of the Natufian culture (sampled from the Raqefet Cave in southern Levant and forming a distinct genetic group from their neighbouring Anatolian HGs)^42^; and (iv) Central Zagros Mesolithic/Neolithic individuals and Caucasus HGs, deriving primarily from a single gene pool and forming a unique genetic cluster distinct from all the aforementioned groups^43, 44^. For the present study, we also include a forager group beyond West Eurasia, that is North African Late Palaeolithic Iberomaurusian HGs (sampled from the Taforalt cave in northwestern Africa), previously identified as having genetic proximity to Levantine Natufians, plus a substantial genetic component from sub-Saharan Africa^45^.

Our principal components analysis (PCA) (Fig. 2, main manuscript) captures successfully the overall expected genetic variation of these groups, based on previous analyses^40-42, 46, 47^. In particular, Iberomaurusian hunter gatherers (HGs), Balkan HGs, and groups from Mesolithic/Neolithic Central Zagros and Caucasus appear at the edges of the plot, highlighting their ancestral nature, while Epipaleolithic HGs from Anatolia (Pınarbaşı) and the Levant (Natufians), occupy a more central position in the plot, clustering closely with early Neolithic groups from the same locations, highlighting clearly the (relative) genetic continuity between the late Pleistocene and early Holocene observed in these regions, as previously noted^41, 42^. It should also be noted that although the Epipaleolithic/Mesolithic population groups included in our analyses appear genetically distinct, some of these (e.g. Anatolian HGs and Balkan HGs) share deep ancestral routes from prior Palaeolithic populations^41, 48, 49^. This however does not compromise at all their use as ancestral sources for later Neolithic populations, as due to differential admixture history and genetic drift over the millennia, they did form distinct genetic groups by the late Epipaleolithic/Mesolithic.

Our *qpAdm* distal admixture analysis (Fig. 3, main manuscript; Supplementary Table S3), reveals an admixture model comprising groups from Epipaleolithic Central Anatolia, Epipaleolithic Levant, and Mesolithic/Neolithic Central Zagros, accounting for the deep ancestry of all Neolithic population groups from the Fertile Crescent and Anatolia under study (discussed in detail below).

*Early Holocene*

During the early Holocene, the agricultural revolution arose in the Near East, approximately 12,000 years ago^50^ during a period of intense climate change, characterised by warmer and wetter conditions and accompanied by substantial environmental change^51^. As a response to these more favourable changes, hunter-gatherer-foragers of the Fertile Crescent began a systematic and unprecedented undertaking of modifying local environments and biotic communities to encourage plant cultivation, animal domestication, and stock-keeping, shifting into a food-producing subsistence economy^50, 52^.

Levant

The Levant, where the earliest evidence of sedentism and crop manipulation emerged^37, 53^, saw high developments and increasing urbanization (in its primal form) during the pre-pottery Neolithic A (PPNA), is generally considered as the core region of the agricultural revolution^54^. The archaeogenetics record consists primarily of PPNB farmers from the southern Levant spanning the 8^th^ millennium BCE, from the sites of ʿAin Ghazal^42^ and Ba'ja^41, 55^ (modern day Jordan), and the sites of Tel Motza^42^ and Kfar HaHoresh^41^ (modern day Israel). A spatiotemporal presentation of these samples can be seen in Fig. 1 in the main manuscript, while further details are available in Table 1 and Supplementary Table S1.

Consistent with previous evidence^42^, our detailed spatiotemporal analysis (Figs. 2 and 3, main manuscript; Supplementary Fig. S3, Supplementary Table S3) reveals that Levantine PPNB farmers harbour primarily local Natufian HG ancestry, with substantial however Anatolian HG admixture and minor Mesolithic/Neolithic Central Zagros admixture. This genetic composition is not entirely uniform across the Levant (Fig. 3, main manuscript; Supplementary Table S3), with some groups showing a more basal profile than others, characterised by higher Natufian-like ancestry and lower admixture from outside (e.g. Anatolia and the Zagros). A PPNB individual from Tel Motza (modern day Israel), is an example of this phenomenon, showing high Natufian admixture (∼70%) and very minor ancestry from the Zagros. A similarly basal profile is seen among two individuals from the southern Levantine site of Ba'ja (Jordan), who however also show some minor ancestry (9%) from Zagros (additional to the Anatolian ancestry mentioned above).

Unlike these two cases, individuals from ʿAin Ghazal (Jordan), a large settlement in southern Levant, show relatively lower Natufian ancestry (60%) and higher admixture from Epipaleolithic Anatolian (32%) and Mesolithic/Neolithic Zagros (8%) groups. An even more admixed genetic composition is seen in an early PPNB individual from Kfar HaHoresh, residing further to the north, still in southern Levant (Israel), with even lower Natufian ancestry (51%) and substantial ancestry from Mesolithic/Neolithic Zagros (16%) and particularly Epipaleolithic Anatolia (33%), appearing to shift clearly towards Neolithic Anatolian groups in the PCA plot (Fig. 2, main manuscript). Our admixture dating analysis, reveals that the influx of Anatolian/Zagros-related admixture into the Levant started occurring around 10,000 BCE ± 700 years (Supplementary Table S11).

Interestingly, the two groups that show increased admixture from Anatolia and Zagros, are chronologically earlier than the groups that show a more basal genetic profile, indicating thus that the genetic differentiation of different groups in the southern Levant during the early Neolithic might not be temporal, but rather geographical. In other words, the exact genetic profile of different groups was probably shaped by the populations who happened to migrate there. For example, the site of Kfar HaHoresh, showing evidence of high admixture from outside the Levant, is located further north relative to the other sites, so it would be reasonable to hypothesize that migrants from Anatolia and Mesopotamia/Zagros had easier access to this site, rather than to sites located further south (e.g. Ba'ja). Another important aspect to consider is the ‘cosmopolitan’ nature of these sites, with some (e.g. ʿAin Ghazal) showing evidence of immigration and population increase^56^, hence an expected increase in admixture resulting from migrants from more distant sites. The lack of northern Levantine samples does not allow any definite conclusions, but it would be probable that northern Levantine groups had an increased Anatolian/Zagros admixture, compared to southern Levantine groups, due to proximity with these regions.

Upper Mesopotamia

Further to the north, in a region bordering northern Levant, that is southeastern Anatolia (modern day Turkey), belonging to the broader region of Upper Mesopotamia, home of a highly active and innovative early Neolithic culture^57^, the archaeogenetics record, comprises a group of the earliest PPN farmers available in the literature from the sites of Nevalı Çori^55^, Çayönü Tepesi^58^, and Boncuklu Tarla / Mardin^30^ (not to be confused with Central Anatolian Boncuklu Höyük discussed below). Spatiotemporal presentation of these samples can be seen in Fig. 1, while further details are available in Table 1 and Supplementary Table S1

In the present study, we systematically analysed all available samples and consistent with previous evidence^30, 55, 58^, we reveal a uniform genetic profile, characterised by high admixture, split roughly equally between ancestral populations from the surrounding regions (Epipaleolithic Anatolia, Epipaleolithic Levant, and Mesolithic/Neolithic Central Zagros) (Fig. 3, main manuscript; Supplementary Table S3; Supplementary Fig. S1). Our admixture dating analysis, reveals that the admixture events giving rise to Upper Mesopotamian Neolithic groups, started occurring between 10,000-11,000 BCE (Supplementary Table S11).

Our results, therefore, confirm previous findings^58^ indicating that 9^th^ millennium BCE Upper Mesopotamian sites appear as ‘melting pots’ of populations from around the Fertile Crescent, resulting in exchange of ideas and practices. This could potentially explain the pioneering role of these regions as regards agricultural innovations and animal husbandry^38^, as well as advanced ritual practices^59^.

Zagros

Further to the east, bordering Upper Mesopotamia, the region of the northwestern Zagros (modern day Iraqi Kurdistan) is also home to very early Neolithic groups^60^, with the archaeogenetics record comprising a small number of early farmers from the sites of Nemrik 9 and Shanidar cave^30^ (spatiotemporal presentation of these samples in Fig. 1 and further details in Table 1 and Supplementary Table S1).

Based on our analysis, early Pre-Pottery Neolithic individuals from Nemrik 9 and Shanidar, show a similarly highly admixed genetic profile as Upper Mesopotamian groups residing a few kilometres to the west, comprising Epipaleolithic Anatolian, Levantine, and Mesolithic/Neolithic Central Zagros ancestry. Unlike Upper Mesopotamian Neolithic groups, this three-way admixture is not split equally between the three ancestral components, with Nemrik 9 and Shanidar farmers showing increased ancestry from Central Zagros, further to the east (Supplementary Fig. S1). In fact, Mesolithic/Neolithic groups from the Central Zagros (e.g. Hotu IIIb, Tepe Abdul Hosein) comprise about two thirds of the ancestry of their northwestern neighbours (Fig 3. main manuscript; Supplementary Table S3).

The region of the Central Zagros (modern day Iran) is considered a key area as regards early crop and animal domestication practice processes and is well characterised archaeogenetically, including a probable Mesolithic individual from the Hotu cave (Hotu IIIb)^42^, and early Neolithic farmers from Ganj Dareh^42^, Tepe Abdul Hosein^61^, and Wezmeh cave^61^, as well as a low coverage sample from Bestansur^30^, further to the west (not included in the current analysis due to low coverage).

In our PCA plot (Fig 2. main manuscript) and MDS plot (Supplementary Fig. S3), all aforementioned Central Zagros groups cluster at the edge of each plot, positioned close to Caucasus HGs, indicating a clear genetic continuity from the Pleistocene to the Holocene and unlike groups from the western Fertile Crescent, no admixture is evident from any outside region during the early Neolithic (Fig 3. main manuscript; Supplementary Table S3). The abovementioned northwestern Zagros farmers (Nemrik 9 and Shanidar), appear close to Central Zagros farmers in the PCA plot (Fig 2. main manuscript), but do not cluster with them, confirming the genetic input from other contemporaneous groups in the Levant and Anatolia, possibly via Upper Mesopotamia.

Central and Northwestern Anatolia

Central Anatolia, particularly the regions of Cappadocia and the Konya plain, is the first region outside of the core Fertile Crescent (discussed above), to acquire the Neolithic ‘package’, by approximately 8000 BCE, eventually giving rise to extensive advanced Neolithic societies^36^. The archaeogenetics record is quite rich as regards these regions, comprising an Epipaleolithic hunter-gatherer from Pınarbaşı Höyük^41^; and early Aceramic Neolithic farmers from Boncuklu Höyük^41, 62^, Aşıklı Höyük^62^, and Musular^63^; as well as Ceramic Neolithic groups from Çatalhöyük^62^ and Tepecik-Çiftlik^64^. The agricultural way of living spread to northwestern Anatolia (the Marmara region), by about 7000 BCE^65^. The archaeogenetic record comprises genomes from a number of northwestern Anatolian Ceramic Neolithic sites, namely Aktopraklik^48^, Barcin Höyük^30, 47, 48^, Menteşe^30^, and Ilıpınar^30^.

From our analyses, it becomes apparent from the PCA plot (Fig 2. main manuscript) and the MDS plot (Supplementary Fig. S3) that all early Neolithic Anatolian populations are slightly shifted towards Neolithic Fertile Crescent groups, relative to the Central Anatolian Epipaleolithic Pınarbaşı HG, indicating that despite the genetic proximity to their ancestral Anatolian foragers, these groups have input from outside regions (i.e. Levant and Zagros). In more detail, two Anatolian clusters emerge, one comprised of the Epipaleolithic Pınarbaşı HG, Aceramic Neolithic farmers from Boncuklu and Aşıklı Höyük, and Ceramic Neolithic groups from the Marmara region in northwestern Anatolia (Barcin Höyük, Menteşe, Ilıpınar). Cypro-LPPNB individuals from Mylouthkia, as well as very early European farmers (VEEF) are also positioned in the first Anatolian cluster (further discussion about this in the main manuscript). A second Anatolian cluster comprises Aceramic and Ceramic Neolithic groups from Central Anatolia (Musular, Çatalhöyük, Tepecik-Çiftlik) and shifts further towards Levant and to a lesser extent Zagros, indicating higher genetic affinity (compared to the first Anatolian cluster) to population groups from these regions (Fig 2. main manuscript).

Similarly, in our distal admixture analysis (Fig 3. main manuscript, Supplementary Table S3), we confirm the previously reported high genetic affinity between early Boncuklu Höyük farmers and the Central Anatolian Epipaleolithic HG from nearby Pınarbaşı^41^. However, this high genetic affinity is not unform throughout Aceramic Neolithic Central Anatolia, with the nearby ‘twin’ Cappadocian sites of Aşıklı Höyük and Musular, showing relatively high admixture from outside Anatolia. In particular, although early farmers from Boncuklu appear to derive 84% of their ancestry from earlier Central Anatolian HGs (with the remaining 16% derived from Mesolithic/Neolithic Zagros), this basal Anatolian ancestry drops to 69% in Aşıklı Höyük and even further to 48% in Musular. The sites of Musular, actually constitutes the earliest evidence (always based on existing data) of extensive population admixture from dual sources (the Zagros and the Levant) in Central Anatolia. Our admixture dating analysis, reveals that the influx of Zagros-related admixture into Central Anatolia occurred around the same time, if not earlier (as evident in Boncuklu) than the case of southeastern Anatolia / Upper Mesopotamia (see sub-section above). Based on our findings, the earliest Zagros-related admixture in Central Anatolia, evident in Boncuklu, started occurring as early as 12,500 BCE ± 1700 years (Supplementary Table S11).

As in the case of the Levant, given that the 3 aforementioned Aceramic Neolithic sites are roughly contemporaneous, points to these genetic differences being a result of differential migrations from outside of Anatolia into specific sites, probably as a result of proximity and other yet unknown sociocultural reasons. These migrations have been recently suggested to stem from population movements from southeastern Anatolia / Upper Mesopotamia^30^. Interestingly, the archaeological record is consistent with these findings, pointing to low-level crop cultivation and limited animal management at Boncuklu and much more advanced and systematic agricultural practises, with a wide range of crops and significant investment in herding, at Aşikli Höyük^36^. These observations, in light of the present genetic findings, could indicate that the influx of Levantine/Zagros groups in Central Anatolia was instrumental in the adoption of agriculture by local foragers.

Later, Ceramic Neolithic, farmers from Çatalhöyük, (Konya plain) and Tepecik-Çiftlik (Cappadocia), show a very similar genetic profile with Aceramic populations residing in the same regions, supporting archaeological evidence for continuity between the Aceramic and Ceramic Neolithic of Central Anatolia^36^. This transition period between the Aceramic and Ceramic Neolithic in Central Anatolia is beyond the scope of the current paper, as this phenomenon postdates the formation of the early Neolithic in Cyprus.

Ceramic Neolithic groups from the Marmara region in northwestern Anatolia (Barcın, Menteşe, Ilıpınar) show lower input from Levantine and Zagros sources (e.g. 15% respectively in the case of Barcin), deriving the majority of their ancestry (70%) from Epipaleolithic Anatolian HGs. Based on our findings, the Zagros/Levant-related admixture, started occurring in northwestern Anatolia, around 11,500 BCE ± 1600 years, from populations form southeastern Anatolia / Upper Mesopotamia (Supplementary Table S11).

A more basal genetic profile (93% ancestry from Epipaleolithic Pınarbaşı HG) is seen in an early Neolithic individual from Aktopraklik, at the time of transition from the Aceramic to Ceramic Neolithic in northwestern Anatolia, indicating differential admixture dynamics in different sites of the Marmara region. This specific individual also shows evidence of increased affinity with Balkan HGs (Supplementary Table S3), which might result from shared deep ancestry between Anatolian and Balkan HGs^41, 48^. If we take this individual as representing the initial Ceramic Neolithic population of Marmara, it could be suggested that, as in the case of Central Anatolia, the influx of migrants from the east (Upper Mesopotamia^66^), might have sparked major developments in the region^36, 65^.

Cyprus

Around the same time as the ‘Neolithic package’ arrived in Anatolia, groups of early Neolithic farmers from the Near East embarked in systematic and increasingly larger scale seafaring journeys, reaching the eastern Mediterranean island of Cyprus by about 9000 BCE, along with their tools, crops, domesticated livestock and even domesticated wild animals, such as dogs and cats^67, 68^. All aforementioned Near Eastern Pre-Pottery Neolithic populations could potentially have managed a long-distance (by Neolithic era standards) seafaring journey and subsequent migration to Cyprus^12, 69-71^. Archaeological evidence from the earliest Cypriot sites (e.g. Ayios Tychonas-*Klimonas*, Ayia Varvara-*Asprokremmos*) points to the Levant as the most likely origin of these settlers, suggested by morphological aspects of lithic assemblages found on Cyprus, domestic architecture, and the zooarchaeological record^1, 4, 68, 70^, while maritime connections with Anatolia, indicated by the presence Central Anatolian obsidian^72^, are also apparent. Relevant archaeological evidence on the possible homelands of these early Cypriot settlers can be found in sections 3 and 4, below. Currently, there is no archaeological consensus on whether very early Eastern Mediterranean seafarers originated from both areas and their exact origin is still under fierce investigation and subject to debate^12, 69-71, 73^.

In terms of the archaeogenetic record, only three Neolithic samples are available in the literature, recovered from the western Cypro-PPNB site of Kissonerga-*Mylouthkia*^30^ (discussed in detail in section 1 of this Supplementary Information). Being the main focus of the present study, genetic findings on these samples are included in the main manuscript text.

**3. Literature overview on uniparental marker ancestry among Cypro-LPPNB Mylouthkia and surrounding population groups**

In terms of paternal ancestry, two out of three analysed Cypro-LPPNB farmers were assigned to Y-haplogroup H2 (H-P96). The third was preliminary assigned to macrohaplogroup F^30^ due to low coverage and is excluded hereafter. From the ancient samples analysed in the present study, Y-haplogroup H is found in 33% of analysed Levantine PPNB farmers ^41, 42, 55^ and 19% of analysed Anatolian Neolithic farmers from Barcin^30, 47, 48, 74^ (Supplementary Fig. S12**;** Supplementary Table S13) and according to previous evidence^75^, could be an indicator of Anatolian ancestry in both Cypro-LPPNB and Anatolian-admixed Levantine PPNB farmers.

In terms of mitochondrial DNA (mtDNA) (Supplementary Fig. S13; Supplementary Table S14), two out of three Cypro-LPPNB individuals providing informative markers, were assigned to haplogroups T2 and X2b, respectively^30^. These haplogroups are probably associated with the Anatolian Neolithic, as among the analysed samples, T2 is found in 13% of Aceramic and 15% in Ceramic Neolithic Central Anatolians^62, 64^, 7% in northwestern Anatolians (Barcin^30, 47, 48, 74^), and sporadically (1 individual at each site) in southeastern Anatolia / Upper Mesopotamia (Nevali Cori^55^), the Central Zagros (Tepe Abdul Hosein^61^), and initial Neolithic Bulgaria^30, 40^. Haplogroup X2b has only been identified in an early Neolithic individual from Revenia^74^, northern Greece.

Overall, the reviewed uniparental marker evidence corroborates our autosomal analyses, highlighting a primarily Anatolian ancestry for the tested Cypro-LPPNB.

**4. Overview of the evidence on the plausibility of different admixture scenarios for Cypro-LPPNB Mylouthkia, as revealed in the present study**

Supplementary Information Table 4 below, provides an overview of different scenarios on the ancestral sources of Cypro-LPPNB, as revealed in the present study (including the predominating scenario described above), as well as alternative scenarios supported by other lines of evidence.

Supplementary Information Table 4. Admixture scenarios for Cypro-LPPNB Mylouthkia

|  | Likely ancestral sources | Supporting evidence | Limitations |
| --- | --- | --- | --- |
| Admixture scenarios for Cypro-LPPNB backed by the present study | | |  |
| *1. Two-way admixture between PPN Central Anatolian and Epipaleolithic Levantine sources* | Major Aceramic Neolithic Central Anatolian genetic component (e.g. represented by PPN Boncuklu Höyük)  Minor basal Levantine component (e.g. represented by Epipaleolithic Natufians) | High level of allele sharing between Cypro-LPPNB Mylouthkia and PPN Boncuklu (Supplementary Table S4)  Very good two-way admixture model fit between Anatolian Boncuklu and Levantine Natufians (Supplementary Table S7)  Timing of specific admixture event consistent with archaeological evidence (Supplementary Table S11)  Supporting archaeological (early lithic assemblage and related toolset, as well as domestic architecture consistent with Levantine practices; later presence of Central Anatolian obsidian) and zooarchaeological (presence of animal species first attested in Levant, such as suids, domestic dogs, cats, and the commensal house mouse in early Neolithic Cypriot sites) evidence^4, 7, 68, 72, 76^ (Sections 5 and 6, below)  Maritime travelling from south-central Anatolia and the Levantine shores to Cyprus consistent with prehistoric seafaring simulation models^12, 69-71, 73^ (Sections 5 and 6, below) | Limited archaeological evidence for permanent Epipaleolithic settlements on the island (Section 5, below)  Limited archaeological evidence for clear Anatolian cultural expressions in early Neolithic Cypriot settlements (Section 5, below) |
| *2. Two-way admixture between PPN Central Anatolian and PPNB Levantine sources* | Major Aceramic Neolithic Central Anatolian genetic component (e.g. represented by PPN Boncuklu Höyük).  Minor basal-like PPNB Levantine component characterised by low admixture from outside the Levant | High level of allele sharing between Cypro-LPPNB Mylouthkia and PPN Boncuklu (Supplementary Table S4)  Good two-way admixture model fit between Anatolian Boncuklu and some PPNB Levantine groups (Supplementary Table S7)  Supporting archaeological and zooarchaeological evidence^4, 7, 68, 72, 76^ (as in scenario 1 above, see Sections 5 and 6, below)  Consistent with prehistoric seafaring simulation models^12, 69-71, 73^ (as in scenario 1 above, see Sections 5 and 6, below) | Statistical plausibility of admixture model slightly weaker than for scenario 1 above (Supplementary Table S7)  Somewhat inconsistent with estimated timing of admixture event - Levantine PPNB postdates the estimated admixture timing (Supplementary Table S11)  Limited archaeological evidence for clear Anatolian cultural expressions in early Neolithic Cypriot settlements (Section 5, below) |
| Other admixture scenarios for Cypro-LPPNB | |  |  |
| *3. Direct ancestry from south-central Anatolian sources* | Theoretical genetic composition very similar to that of Cypro-LPPNB, from Aceramic Neolithic sites in south-central Anatolia in close proximity to the southern shores of Anatolia, a few kilometres to the north of Cyprus, such as Pınarbaşı | High level of allele sharing between Cypro-LPPNB Mylouthkia and Epipaleolithic Pınarbaşı (Supplementary Table S4)  Strong prehistoric seafaring simulation support^12, 69-71, 73^ (Overview in section 5, below) | No available genetically characterised samples from early Neolithic Anatolian sites with close proximity to Cyprus (with the exception of Boncuklu) |
| *4. Direct ancestry from PPN northern Levantine sources* | Theoretical genetic composition very similar to that of Cypro-LPPNB, from major northern Levantine PPNA sites, such as Mureybet and Abu Hureyra | Strong archaeological support (e.g. cultural affinities between earl PPN Cypriot settlements and northern Levantine sites, such as Mureybet^8^)  Prehistoric seafaring simulation support^12, 69-71, 73^ (Overview in section 5, below) | No available genetically characterised samples from northern Levant  Available genomes from nearby sites in SE Anatolia / Upper Mesopotamia (e.g. Nevali Cori) have a genetic profile uncharacteristic of Cypro-LPPNB due to excess basal Zagros admixture (Supplementary Table S7) |

**5.** **Overview of archaeological evidence on the origins of the earliest seafarers reaching Cyprus**

Cyprus has been isolated from the surrounding mainland by open sea since the Late Miocene–Pliocene^69^, fulfilling thus the criteria of a ‘true’ oceanic island^12^. Despite this, two reviews of the latest evidence^69, 71^, conclude that during the Pleistocene, on a clear day, the northern coast of Cyprus would have been visible from certain locations at the southern Anatolian coast (e.g. between Tasucu Bay and the eastern end of the Gulf of Antalya), while the eastern Cypriot peninsula (Karpasia) would have been visible both from the Nur Mountains (southeastern Anatolia / northern Levant) and the Syrian Coastal Mountain Range (Central Levant). The easternmost tip of the island (Cape Apostolos Andreas) would have been visible from the coastal location of Cape Ras al Basit in northern Levant^69^. The mountains of Cyprus would also have been visible during significant parts of the maritime journey between the mainland and Cyprus, guiding early seafarers towards their ultimate destination^71^.

Archaeological evidence shows that Epipaleolithic hunter-gatherers from the mainland crossed the open sea and reached Cyprus, systematically, since the late Pleistocene (mid-11^th^ millennium BCE), as documented at the site of Akrotiri-*Aetokremnos* in southern Cyprus^1, 12^. More recently, a confirmed Epipaleolithic mountainous site, Vretsia-*Roudias*, was identified further inland^10^, while a possible coastal Epipaleolithic site at the western tip of the island (Akamas-*Aspros*) is currently under archaeological investigation^11^. Moutsiou et al.^77^, applying predictive modelling to map the likely distribution of Pleistocene archaeological locales on Cyprus, argue that the density of mobile hunter-gatherer sites on Cyprus is much greater than the limited number of currently excavated sites^1, 10, 11^ and therefore easily underestimated.

These Epipaleolithic settlements (and possibly more to follow) gave way to more organised settlements, during the early Holocene such as Ayios Tychonas-*Klimonas*, Ayia Varvara-*Asprokremmos*^2, 3^, some of which presenting themselves as organised Cypro-PPNA villages of hunter-cultivators (e.g. *Klimonas*), during the first half of the 9^th^ millennium BCE^4^. These were followed by even more organised, agricultural settlements during the Cypro-PPNB, such as Parekklisha-*Shillourokambos*^5^, Kalavasos *Tenta*^13^, Kritou Marottou-*Ais Giorkis*^15^, Akanthou *Arkosyko*^14^, and possibly Kissonerga-*Mylouthkia*^6^ (the only Neolithic Cypriot site with genetically analysed human remains at the time of writing of the current manuscript - see section 1 in this Supplementary Information).

During the Epipaleolithic, with surely less advanced seafaring technology than the later PPN, a trip from the Levant appears a huge undertaking given that at least 60-70 km of open sea separated Cyprus from the nearest coast of northern Levant, even at periods of intense marine regression (compared to just 30-40 km from southern Anatolia)^70, 71^. Additionally, geological and glacio-hydro-eustatic evidence, including bathymetric and palaeo-shoreline reconstruction^78, 79^ reveals that a small steppingstone island likely existed between the Iskenderum basin (bordering northern Levant and southeastern Anatolia) and Cyprus at the time the Epipaleolithic seafarers had reached the island around 10,500 BCE. This islet would have reduced the total journey distance to 42 km from northern Levant to the islet and 25 km from the islet to Cape Apostolos Andreas at the easternmost tip of Cyprus^70^.

Studies aiming to evaluate all available evidence for testing the plausibility of different maritime routes between Cyprus and the mainland, including simulation of ancient sea levels and currents, paleo-coastline reconstruction, ancient prevailing seasonal and diurnal wind, possible sailing routes, watercraft types, and navigational skills, conclude that a trip from the Levantine coast to Cyprus could be possible but surely much more difficult compared to a trip from southern Anatolia^71, 73^. Applying virtual sea-drifting experiments, Kyriakidis et al^73^ conclude that a trip from the Levantine coast to Cyprus would have only been possible from very specific locations in northern (not southern or central) Levant, at the southernmost tip of the Antakya Bay, just south of the İskenderun Bay. Such a drifting trip on a small wooden raft (approximately 2 m long, 1 m wide, and 20 cm thick) capable of carrying up to 5 people, would have lasted at least 4 days and would have been plausible only during specific periods in the year (e.g. winter), conditional to the early Holocene currents being the same or very similar to those observed today^73^. It should be noted however, that others propose a much more sophisticated maritime technology, characterized of vessels carrying a sailing mast^70, 71^ and having small storage rooms (also facilitating the unintentional introduction of the house mouse)^68^. These authors convincingly suggest that a simple sea-drifting journey, would have been impossible given the load of Neolithic vessels (large livestock, sacks of crops, obsidian, etc.). Particularly weaning animals (more probable than adult animals), onboard wouldn’t survive a journey of more than a couple of days^68^.

Knapp^12^ proposes that the homelands of specific Levantine groups were threatened by rising sea level towards the end of the Younger Dryas, with their subsistence base being under threat, which forced them to pursue and advance seafaring, reaching Cyprus around the time at the end of the cool and arid Younger Dryas climate episode. Additionally, Vigne et al.^70^ go further to suggest that early systematic Eastern Mediterranean seafarers might have formed specialised groups or even communities, somehow distinct from farmers dealing with agricultural innovations, with the main aim of acquiring and perfecting navigation, who must have had at least basic knowledge of winds, currents, landmarks and possible landing places, even harnessing wind power with primitive sails, which would have made the journey from the mainland much quicker, an important factor when transporting weaned animals^12, 70^.

The zooarchaeological record is consistent with this hypothesis, as several species introduced from the Epipaleolithic through the initial Neolithic on the island are apparent in the Epipaleolithic and early Neolithic record of the Levant but appear at Anatolian Neolithic sites only several centuries after they were introduced to Cyprus^80-82^. For example, suid bones have been confirmed at Epipaleolithic Akrotiri-Aetokremnos^76^ with Vigne at al.^68^ concluding that the most probable scenario is that wild boars were introduced by humans to Cyprus towards the end of the Late Glacial from the (northern) Levant, corresponding to the Natufian era. These early introduced wild boars, showed clear evidence of insular dwarfism when sampled for the first time at Aetokremnos, where they were hunted/butchered by the very early seafarers reaching there^68^. This highlights an introduction well into the Epipaleolithic (i.e. prior to 11,000 BCE), with northern Levant being the most likely origin of very early seafaring to Cyprus.

Further support for a continuing connection of Cyprus with Epipaleolithic / early PPNA Levant, comes indirectly from the presence of domestic dogs (*Canis familiaris*), e.g., at the PPNA site of Ayios Tychonas-*Klimonas*, as well as the commensal house mouse (*Mus musculus domesticus*) and its main predator, the commensal Near Eastern cat (*Felis s. lybica*)^4^. Interestingly, the current consensus based on all available zooarchaeological evidence, suggests that the commensal nature of all three species with humans originated among Natufians in the Levant, at least as far as the Near East is concerned^80-82^. As regards Cyprus, Vigne et al^68^ propose that early domestic dogs were brought by humans most likely from the Levant to aid in the hunting of the wild boar (themselves introduced from the same region a couple of millennia earlier), while commensal cats were introduced for catching mice, already acting as pests in the early insular Neolithic societies. Additionally, the widespread introduction of the Mesopotamian fallow deer (much more abundant in the Levant than in Central Anatolia) and sheep into the island a few centuries later, also points to a Levantine origin of the initial Neolithic seafarers^68, 83^. Along the same lines, evidence of early crop introduction, such as emmer wheat and hulled barley (most abundant in the Levant than Anatolia)^84, 85^ in the initial Neolithic Cypriot sites, points to a probable Levantine (or at least southeastern - but not Central - Anatolian) origin of the first settlers.

Furthermore, at the sites of Ayios Tychonas-*Klimonas* and nearby *Throumbovounos*, lithic assemblage and chipped stone tools show close parallels to northern Levant (Mureybet and Sheikh Hassan)^4^, characterised by the Khiamian culture, marking the transition between the Natufian and the Levantine PPNA^68^. Particularly at PPNA Klimonas, the lithic industry and presence of a shaft straightener, with unidirectional blade debitage, as well as small arrowheads with a short tang, show very clear parallels with the Levantine PPNA tradition^2^. Additionally, once colourful conic artefacts, discovered at the site and interpreted as Neolithic stone figurines, probably of symbolic use, appear similar to objects observed in Levantine PPNA sites, particularly Mureybet^2^.

Finally, the circular architecture observed at Cypro-PPNA sites, has been interpreted as an indicator of Levantine PPNA heritage of their inhabitants^12^. For example, a large (10m diameter) communal building at Klimonas closely resembles similar communal structures at Levantine PPNA sites^2^.

**6. Overview of archaeological evidence on the maritime connections between Anatolia and Cyprus during PPNB**

Like in the case of the Levant, Cyprus was visible in clear weather from certain parts of mainland southern Anatolia (e.g. Tasucu Bay and the eastern end of the Gulf of Antalya). In a comprehensive review of the maritime connections between Cyprus and the mainland during the Neolithic, considering all available archaeological, paleoclimate and paleo-coast evidence, Bar-Yosef Mayer et al^71^ conclude that the optimal Neolithic maritime route to Cyprus would have been from southern Anatolia (as compared to any trip from the Levantine coast), particularly between April and October. Similar conclusions have been reached by Kyriakidis et al^73^ through virtual sea-drifting simulation, this time identifying likely departure and landing spots on the mainland and Cyprus, respectively. This work points to a location on the western edge of the Mersin Bay (corresponding to the southwestern boundaries of the Cilician plain) as the most plausible source for a successful sea-drifting trip to the northern coast of Cyprus, on a small wooden raft. According to the same simulations and considering contemporary currents, such a trip would have been much more successful during the autumn.

Despite the above, the archaeological record does not indicate close parallels between either the Epipaleolithic or the initial PPN sites of Cyprus and those of south-central Anatolia. As described above, Cypriot PPNA sites (e.g. Klimonas, Asprokremmos), had clear Levantine cultural affiliations. However, even at the very early site of Klimonas, there is evidence of small amounts of imported Anatolian obsidian^2^, which accumulated to very high quantities in later PPNB sites, such as Shillourokambos, Kalavasos-*Tenta*, and Kissonerga-*Mylouthkia* (genetically analysed in the current study)^72^. Importantly, by far the highest amounts of Anatolian obsidian was found at the site of Akanthou-*Arkosyko* on Cyprus’ north coast, having obviously the closest proximity to Anatolia. This provides unequivocal evidence of maritime links between Cyprus and Anatolia during the early Neolithic^12, 72^. Moutsiou^72^ further suggests that the presence of obsidian artefacts deriving from multiple Central Anatolian sources and widely dispersed in the island, with decreasing frequency from north to south, indicates that early Neolithic Cypriots engaged in a wide trade network and general social landscape with its neighbouring Anatolian mainland and beyond.

Recent chemical and fluorescent spectroscopy analyses pinpoint the source of imported Cypriot obsidian during the Neolithic, as deriving from different sites in Central Anatolian Cappadocia (e.g. Göllü Dağ, Nenezi Dağ, and Kömürcü-Kaletepe)^14, 72^.

**7. List of Supplementary Tables and Figures**

Table S1: List of ancient samples used in the present study and their relevant characteristics.

Table S2: Characteristics of Epipaleolithic/Mesolithic and Neolithic population groups used in the main analyses.

Table S3: *qpAdm* admixture weights and standard errors showing distal genetic composition for all analysed Neolithic populations, as derived from 3 ancestral Epipaleolithic/Mesolithic sources from Anatolia, the Levant, and Central Zagros.

Table S4: Outgroup *f3*-statistics (outgroup; test, comparison) for estimating shared genetic drift between Mylouthkia Cypro-LPPNB (target) and a list of all ancient populations used in the current study (comparisons) from an outgroup population (Mbuti), sorted by genetic affinity.

Table S5: Matrix of 1-*f3*-statistic (Mbuti; test, comparison) displaying pairwise genetic distances between all Near Eastern Neolithic/Mesolithic and European earliest Neolithic populations used in the current study.

Table S6: *f4*-statistics (outgroup pop, test pop; comparison pop 1, comparison pop 2) for estimating comparative shared genetic drift between Mylouthkia Cypro-LPPNB (test pop) and available Epipaleolithic and early Neolithic Anatolian and Levantine potential sources (comparison pops 1 and 2), compared to an outgroup population (Mbuti).

Table S7: *qpAdm* admixture weights and standard errors showing proximal genetic composition of Mylouthkia Cypro-LPPNB, after testing all available Epipaleolithic and Pre-Pottery Neolithic sources from Anatolia, Levant, Mesopotamia, and Zagros.

Table S8: Kinship analysis results derived using READ and admixture weights derived using *qpAdm* (best fitting proximal model) for Cypro-LPPNB Mylouthkia individuals (n=3).

Table S9: Admixture weights and standard errors for the best fitting proximal *qpAdm* models for Mylouthkia Cypro-LPPNB, PN Anatolia Marmara, and PN Central Anatolia, applied respectively to each other.

Table S10: Model fit of constructed admixture graphs involving Cypro-LPPNB Mylouthkia and other relevant ancient populations, as presented in Supplementary Figs. S5-S9.

Table S11: Inferred time of admixture between an ancestral Levantine and an ancestral Anatolian population for Mylouthkia Cypro-LPPNB as target.

Table S12: *f4*-statistics (outgroup pop, test pop; comparison pop 1, comparison pop 2) for estimating comparative shared genetic drift between very early European farmers and either Mylouthkia Cypro-LPPNB (comparison pop 1) or any of the available Epipaleolithic and early Neolithic Anatolian sources (comparison pop 2).

Table S13: Y-Haplogroup frequencies among the main population groups analysed in the present study by archaeological period and region.

Table S14: mtDNA-Haplogroup frequencies among the main population groups analysed in the present study by archaeological period and region.

Supplementary Figure S1: Ratio of basal Levant to Zagros ancestry among analysed Pre-Pottery Neolithic populations.

Supplementary Figure S2: Outgroup *f3*-statistics (Mbuti; test, comparison) displaying pairwise genetic distances between Cypro-LPPNB and all Epipaleolithic/Mesolithic and Neolithic population groups included in the study.

Supplementary Figure S3: MDS plot based on outgroup f3 (Mbuti; test, comparison) displaying two-dimensional genetic distances between all Near Eastern Epipaleolithic/Mesolithic/Neolithic and earliest European Neolithic populations.

Supplementary Figure S4: *f4*-statistics displaying shared genetic drift between potential Epipaleolithic/Mesolithic/PPN Near Eastern sources and Cypro-LPPNB.

Supplementary Figure S5: Admixture graphs involving Cypro-LPPNB, constructed following a manually arranged topology using *qpGraph* (panel a) or with an automated approach using *findGraphs*, involving 3 admixture events (panels b-e).

Supplementary Figure S6: Admixture graphs involving Cypro-LPPNB, constructed following an automated approach using *findGraphs*, involving 4 admixture events.

Supplementary Figure S7: Admixture graphs involving Cypro-LPPNB, constructed following an automated approach using *findGraphs*, involving 5 admixture events.

Supplementary Figure S8: Admixture graphs involving Cypro-LPPNB, with Aşıklı Höyük as the Central Anatolian source rather than Boncuklu, constructed following an automated approach using *findGraphs*, involving 4 and 5 admixture events.

Supplementary Figure S9: Admixture graphs involving Çatalhöyük or Barcin instead of Cypro-LPPNB, constructed following an automated approach using *findGraphs*, involving 4 admixture events.

Supplementary Figure S10: Inferred admixture timing ±1 SE for Cypro-LPPNB and other contemporaneous Near Eastern populations.

Supplementary Figure S11: *f4*-statistics displaying shared genetic drift between Cypro-LPPNB samples vs Anatolian PPN/PN populations and very early European Neolithic populations.

Supplementary Figure S12: Frequency of major Y-chromosome haplogroups among Near Eastern and Southeast European Epipaleolithic/Mesolithic and early Neolithic populations analysed in the present study.

Supplementary Figure S13: Frequency of major mitochondrial DNA haplogroups among Near Eastern and Southeast European Epipaleolithic/Mesolithic and early Neolithic populations analysed in the present study.

**8. Detailed Methods**

*Ancient sample selection and compilation of working dataset*

In order to achieve our main aim of elucidating the exact ancestry of previously genetically sampled Cypro-LPPNB individuals^30^, as well as test genetic connections with other ancient surrounding populations, we utilised published publicly available data, from 3 different resources:

1. The Harvard Dataverse repository (<https://dataverse.harvard.edu/>)
2. The Allen Ancient DNA Resource (AADR) (<https://doi.org/10.7910/DVN/FFIDCW>)^86^
3. The European Nucleotide Archive (ENA) (<https://www.ebi.ac.uk/ena/browser/home>)^87^

The three Cypro-LPPNB samples from the site of Kissonerga-*Mylouthkia*, the main focus of the present study, were derived from the first resource, specifically the working dataset from the original publication: Lazaridis I; Alpaslan-Roodenberg S; Pinhasi R; Reich D, 2022, "The genetic history of the Southern Arc: A bridge between West Asia and Europe"^30^, <https://doi.org/10.7910/DVN/3AR0CD> (Harvard Dataverse, V1).

The specific dataset, also includes an additional 724 ancient individuals from Anatolia, southeastern Europe and West Asia, spanning 10,000 years^30^. Genomic data comprise 3 files in eigenstrat format: (i) ‘geno’ file: genotype data in binary form ('packedancestrymap' format); (ii) ‘snp’ file: information on analysed SNPs (SNP id, physical/genetic location, reference/variant alleles, based on hg19); (iii) ‘ind’ file: basic characterisation of all individual samples included (individual ID, sex determination, group label - population). Selected Epipaleolithic/Mesolithic and early Neolithic Near Eastern and southeastern European samples of interest were retrieved from the specific dataset, for the purposes of the present study (see further below for specific selection criteria).

Additional Epipaleolithic/Mesolithic and early Neolithic samples of interest not included in the above dataset, were retrieved from the comprehensive AADR curated dataset^86^. The dataset consists of 4 files, in eigenstrat format, as described above: (i) ‘geno’ file; (ii) ‘snp’ file; (iii) ‘ind’ file; and (iv) ‘anno’ file (detailed meta-information for all individual samples included).

For additional recently published genomes (e.g. Aktopraklik, Musular, Çayönü, Nevalı Çori) were not available in AADR, or in case where higher coverage versions of samples of interest were not available in the specific resource (e.g. Aşıklı Höyük - Ash128), the relevant raw data (BAM files of aligned reads) were retrieved from the European Nucleotide Archive (ENA)^87^. ENA provides a comprehensive record of global nucleotide sequencing information, covering raw sequencing data, sequence assembly information and functional annotation^87^.

Samples to be analysed were selected in a systematic way, following specific inclusion and exclusion criteria. All eligible samples fulfilling both of the following Inclusion criteria were initially selected:

1. *Geographical location*: Samples from the regions of the Fertile Crescent (Levant, Mesopotamia, Zagros), Anatolia, and southeastern Europe
2. *Chronological period*: Samples from the Epipaleolithic, Mesolithic, Pre-Pottery (Aceramic) Neolithic (PPN), early Pottery (Ceramic) Neolithic (PN), initial Early Neolithic (EN) of Europe (older than 5500 BCE)

Following the initial selection, the below exclusion criteria were applied:

1. Samples labelled as ‘contaminated’ in the source curated datasets
2. Samples with very low coverage (<0.01X) or samples with low coverage (0.01-0.05X), which show signs of shifting from their corresponding population cluster in our PCA analysis (described further below)
3. Samples labelled as ‘outliers’ in the source curated datasets (this has been double-checked in our PCA analysis, described below)
4. Early European Neolithic samples (older than 5,500 BCE) labelled as having European hunter-gatherer (HG) admixture (this has been double-checked in our PCA analysis, described below).

*Compilation of the final archaeogenetic dataset*

After retrieving all samples fulfilling the above criteria from the 3 abovementioned resources, we followed the below procedure to compile the final merged analysis dataset:

- For all ancient samples retrieved from the ENA database, available only as trimmed BAM files of aligned reads, Samtools *mpileup* ([http://www.htslib.org/doc/samtools-mpileup.html)](http://www.htslib.org/doc/samtools-mpileup.html)80)^88^ was used to create pileups of BAM files of ds libraries format, ignoring RG tags and applying mapping quality for an alignment and base quality thresholds of 30.
- The tool *pileupCaller* (<https://github.com/stschiff/sequenceTools/tree/master/src/SequenceTools>) was used for extracting genotypes from pileups (random haploid option) and saving into eigenstrat format
- The tool *mergeit* from the EIGENSOFT (v.6.01) package^89, 90^ (<https://github.com/argriffing/eigensoft/tree/master/CONVERTF>) was used to merge the resulting dataset from the above procedure with the datasets retrieved from the Harvard Dataverse repository and the AADR database, creating the final analysis dataset.

Our final working dataset comprises 179 ancient samples, with our main samples of interest spanning a period of c. 7,000 years (13,000–6000 BCE). More specifically, our working dataset includes:

- 11 population groups from the Palaeolithic and Epipaleolithic/Mesolithic Near East, North Africa, and Eurasia (TUR_Pinarbasi_EpiP^41^, ISR_Natufian_EpiP^30, 42^, GEO_Kotias^44^, GEO_Satsurblia^44^, IRN_Mesolithic_HotuIIIb^42, 43^, MAR_Taforalt_EpiP^45^, SRB_Iron_Gates_HG^40^, EHG^47^, WHG^44, 91-93^, MA1^47^, AfontovaGora3^92^), comprising a total of 59 samples.
- 17 population groups from PPN Near East (CYP_Mylouthkia_PPNB^30^, TUR_C_Boncuklu_PPN^41, 62^, TUR_C_AsikliHoyuk_PPN^62^, TUR_C_Musular_PPN^63^, ISR_Motza_PPNB^42^, ISR_KfarHaHoresh_PPNB^41^, JOR_AinGhazal_PPNB^30, 42^, JOR_AinGhazal_PPNC^30, 42^, JOR_Baja_PPNB^41, 55^, TUR_SE_NevaliCori_PPNB^55^, TUR_SE_Cayonu_PPNB^58^, TUR_SE_Mardin_PPNA^30^, IRQ_Nemrik9_PPNA^30^, IRQ_Shanidar_PPNA^30^, IRN_Ganj_Dareh_PPN^43^, IRN_Tepe_Abdul_Hosein_PPN^61^, IRN_Wezmeh_PPN^61^), comprising a total of 55 samples.
- 6 population groups from PN Near East (TUR_Mar_Aktopraklik_PN^48^, TUR_C_Catalhoyuk_PN^62^, TUR_C_TepecikCiftlik_PN^64^, TUR_Mar_Barcin_PN^30, 47, 48^, TUR_Mar_Mentese_PN^30, 47^, TUR_Mar_Ilipinar_PN^30^), comprising a total of 40 samples.
- 14 population groups from the initial European EN, labelled as Very Early European Farmers – VEEF (GRC_North_Revenia_EN^74^, GRC_North_NeaNikomedeia_EN^48^, GRC_South_Alepotrypa_EN^40^, BGR_Yabalkovo_EN^30, 40^, BGR_Dzhulyunitsa_EN^40^, BGR_MalakPreslavets_EN^40^, ROU_Carcea_EN^30, 40^, ROU_Cotatcu_EN^40^, ALB_Podgorie_EN^30^, MKD_Govrlevo_EN^48^, SRB_LepenskiVir_EN^48^, HRV_Zemunica_Cardial_EN^40^, HUN_Tiszapuspoki_Koros_EN^94^, ITA_GrottaContinenza_EN^95^), comprising a total of 21 samples.

Modern country abbreviations relevant to the above populations, are as follows: CYP (Cyprus), TUR (Turkey), ISR (Israel), JOR (Jordan), GEO (Georgia), IRN (Iran), IRQ (Iraq), MAR (Morocco), GRC (Greece), ITA (Italy), BGR (Bulgaria), ROU (Romania), ALB (Albania), MKD (North Macedonia), SRB (Serbia), HRV (Croatia), HUN (Hungary). Specific information regarding the above listed population groups and the included ancient individuals can be found in Supplementary Tables S1 and S2.

Just for the purpose of checking the population structure of our ancient samples using principal components analysis (PCA), described below, we merged our dataset with 61 publicly available modern West Eurasian populations^42^ (1080 individuals from the following populations: Abkhasian.HO, Adygei.HO, Albanian.HO, Armenian.HO, Assyrian.HO, Balkar.HO, Basque.HO, BedouinA.HO, BedouinB.HO, Belarusian.HO, Bulgarian.HO, IBS_CanaryIslands.HO, Chechen.HO, Croatian.HO, Cypriot.HO, Czech.HO, Druze.HO, English.HO, Estonian.HO, Finnish.HO, French.HO, Georgian.HO, Greek.HO, Hungarian.HO, Icelandic.HO, Iranian.HO, Italian_North.HO, Italian_South.HO, Jew_Ashkenazi.HO, Jew_Georgian.HO, Jew_Iranian.HO, Jew_Turkish.HO, Jew_Iraqi.HO, Jew_Libyan.HO, Jew_Moroccan.HO, Jew_Tunisian.HO, Jew_Yemenite.HO, Jordanian.HO, Kumyk.HO, Lebanese.HO, Lebanese_Christian.HO, Lebanese_Muslim.HO, Lezgin.HO, Lithuanian.HO, Maltese.HO, Mordovian.HO, Norwegian.HO, Orcadian.HO, Palestinian.HO, Romanian.HO, Russia_NorthOssetian.HO, Russian.HO, Sardinian.HO, Saudi.HO, Scottish.HO, Sicilian.HO, Spanish.HO, Spanish_North.HO, Syrian.HO, Turkish.HO, Ukrainian.HO) from the AADR database (described above), which includes over 10,000 present-day individuals genotyped on the Illumina Affymetix Human Origins array (~500,000 SNPs). Merging was performed using tool *mergeit* from the EIGENSOFT (v.6.01) package^89, 90^ (<https://github.com/argriffing/eigensoft/tree/master/CONVERTF>), keeping the intersection of SNPs between the two panels.

*Principal Components Analysis*

For determining population structure and the overall genetic variation in the analysed ancient samples, we conducted a principal components analysis (PCA) using the *smartpca* tool from the EIGENSOFT (v.6.01) package (<https://github.com/chrchang/eigensoft/blob/master/POPGEN/smartpca.info>)^89, 90^, with option ’*shrinkmode’.*

Since high rates of missingness are common in ancient genomes, potentially leading to bias, the PCA was initially computed on the abovementioned 61 Human Origins modern West Eurasian populations and the ancient individuals included in our working dataset were projected on the modern western Eurasian genetic variation^89, 90^. The first two principal components (PCs) were plotted creating a 2-dimensional plot.

This analysis was repeated twice. For the first run, we confirmed that all individual samples labelled as ‘outlier’, ‘contaminated’, or ‘low coverage’ in the original source datasets, indeed diverged from the cluster of their corresponding population groups and hence were excluded to avoid introduction of bias in subsequent formal statistics analyses. Along the same lines and concentrating specifically on VEEF samples, we excluded any individuals showing evidence of European HG ancestry, i.e. clearly diverging from the main VEEF cluster and shifting towards Balkan HGs. Since a secondary aim of the present study was to investigate genetic connections between Cypro-PPNB and VEEF, any European HG admixture among the latter (which is in fact rather rare during the initial European Neolithic) would introduce bias and lead to misleading results and conclusions.

Once the above samples were excluded, the PCA was performed on a final sample of 166 ancient individuals (total 179 genomes in the final working dataset minus 9 Palaeolithic/Mesolithic Eurasian genomes used simply as references in admixture analyses) and 1080 modern individuals, achieving an ideal population structure among the different groups and revealing an unbiased genetic variation for the working dataset (Figure 2, main manuscript).

*F-statistics*

We used the package ADMIXTOOLS v.2.0.0^96, 97^ in R (version 4.1.1)^98^, operated using RStudio (v. 2022.07.1+554)^99^, to estimate outgroup *f3-*statistics^96, 97^ of the form *f3(outgroup; population A, population B)*, where in our case, outgroup = Mbuti, population A = Cypro-LPPNB, and population B = all ancient populations in our working dataset, in turn.

Outgroup *f3-*statistics estimate the relative divergence time for pairs of populations (Cypro-LPPNB vs other ancient population, in this case), determining pairwise genetic distance^96, 97^. This is achieved by calculating allele frequency correlations between pairs of populations (e.g. A and B) to measure shared genetic drift between them from an outgroup population. If the outgroup of choice is a truly unrelated population to both A and B, outgroup *f3-*statistics will estimate the genetic distance between the outgroup and the points of separation between A and B, providing thus a measure of genetic affinity, based on allele sharing, between the two test populations^96, 97^. The higher the value of the outgroup *f3-statistic* the higher is the genetic affinity (allele sharing / shared drift) between populations A and B. In our case, this test was used to determine genetic affinity between Cypro-LPPNB and all ancient populations of interest in our dataset.

Compared to simpler statistical approaches for estimating pairwise genetic distance (e.g. *Fst* or *f2*), *f3-*statistics are less affected by differences in population size and in particular small sample sizes, as well as from genetic drift experienced by either of the tested populations (A and B)^96, 97^.

In order to provide an overall picture of genetic affinities between tested populations, estimated using formal statistics, we constructed a dissimilarity matrix comprising pairwise outgroup *f3*-statistics of the form 1 - *f3*(Mbuti; pop1, pop2)^49^. This matrix presented in Supplementary Table S5, includes pairwise genetic distances between relevant Epipaleolithic and all Neolithic populations in our comparative dataset, including Cypro-LPPNB. Based on this matrix, we ran Multidimensional Scaling (MDS) analysis using the *cmdscale* function in R 4.1.1. Goodness of fit parameters for this model were estimated as R2=0.77, F=2301, and a p-value of <0.001. We then constructed an MDS plot (Supplementary Fig. S3), displaying genetic affinities between the tested populations in a two-dimensional panel.

In order to further investigate genetic distances between populations, this time in comparative terms, we estimated *f4-*statistics^96, 97^ of the form *f4(outgroup, population A; population B, population C)*. This analysis was performed for two purposes:

(a) Determine allele sharing and consequently genetic proximity between a set of two ancient Near Eastern populations and Cypro-LPPNB Mylouthkia, in comparative terms. In this case: outgroup = Mbuti, population A = Cypro-LPPNB, population B = first comparison ancient Near Eastern population, and population C = second comparison ancient Near Eastern population. In this case, we followed a systematic approach by first grouping population groups into meta-populations (Anatolia, Levant, Zagros/Mesopotamia) and comparing allele sharing between meta-populations, to test which of these as a group, shares more alleles with Cypro-LPPNB. We then compared allele sharing between population groups this time within meta-populations (e.g. Anatolians: TUR_Pinarbasi_EpiP *vs* TUR_C_Boncuklu_PPN, TUR_Pinarbasi_EpiP *vs* TUR_C_AsikliHoyuk_PPN, etc.; Levantines: ISR_Natufian_EpiP *vs* JOR_AinGhazal_PPNB, ISR_Natufian_EpiP *vs* ISR_KfarHaHoresh_PPNB, etc.; Zagros/Mesopotamians: IRN_HotuIIIb_Mes *vs* IRN_Ganj_Dareh_PPN, IRN_HotuIIIb_Mes *vs* IRN_Tepe_Abdul_Hosein_PPN, etc.).

(b) Determine allele sharing and genetic proximity between Cypro-LPPNB and a series of Anatolian populations from the Epipaleolithic to Pottery Neolithic, as regards their comparative genetic affinity with Very Early European Farmers. In this case, outgroup = Mbuti, population A = VEEF population groups included in our dataset (Supplementary Table S1), population B = Cypro-LPPNB, and population C = Anatolian Epipaleolithic/Neolithic populations included in our dataset, in turn.

As in the case of *f3*-statistics, *f4*-statistics are based on correlations of allele frequency differences, thus estimating shared genetic drift, but involving four different populations instead of three^89^. Statistically, *f4* is the covariance of allele frequency differences between two pairs of populations, i.e. *f4*(O, A; B, C).

Assuming no additional admixture, the allele frequency difference between O and A should be independent from the allele frequency difference between B and C, giving a null (0) *f4* value. Since O is an outgroup population, with no evident admixture with either population B or population C, negative *f4* values indicate increased gene flow between populations A and B, while positive *f4* values indicate increased gene flow between populations A and C. In our case, this test was used to examine whether VEEF share more genetic drift (i.e. higher number of alleles) with Cypro-LPPNB than with Neolithic Anatolian populations.

As in the case of *f3-*statistics, the strength of *f4*-statistics is that they are robust to divergence time, small population sizes, missing data, and lower data quality^100^. For all relevant *f*-statistics, standard errors were estimated by the default option of 5 cM block jack-knifing.

*qpAdm*

The tool *qpAdm* included in the package ADMIXTOOLS v.2.0.0^96, 97^, utilizes *f-*statistics (*f4* in particular) to estimate admixture proportions for determining the ancestry of given target populations from a list of source populations. In particular, *qpAdm* determines whether a target population is the product of admixture between a list of source populations included in the model. In order to run such a model, *qpAdm* additionally requires a list of reference (outgroup) populations, whose role is to provide information about the relationships among the target and source populations. The method requires that at least some reference populations are more closely related to some source populations than to others^100^.

A recently implemented methodology, termed ‘rotating model’^100^, requires a single set of populations (i.e. not separated into sources and references). From this set of populations, *qpAdm* randomly selects sources, treating the remaining populations, each time, as references. With this ‘rotating model’ approach, each population is treated both as a source and as a reference (in subsequent models), generating consistent results on a common set of principles, rendering the findings more robust and directly comparable^100^. In fact, using simulation data, the rotating model approach was found to distinguish better between plausible and implausible admixture models, compared to the ‘base’ model approach, in which source populations (‘left’) and reference populations (‘right’) are defined separately^100^. The rotating model approach, if required, can also be set to treat some populations from the list only as references and not use them as potential sources of admixture for the target population. With the rotating model approach therefore, the composition of admixture models to be tested can be considered as being ‘blind’, meaning that it does not rely on prior hypotheses of the researcher.

The strengths of *qpAdm*, from the new ADMIXTOOLS 2 package, include: (i) provision of robust and unbiased estimates even in cases of genetic drift in one or more of the populations included in the model, since these are based on *f4*-statistics; (ii) provision of robust and unbiased admixture estimates even in cases where the target, source, or reference populations have small sample sizes or even in situations of singleton populations (i.e. comprising only 1 individual); (iii) provision of robust and unbiased admixture estimates even in cases where one or more populations in the model has a high rate of missing data^100^. This is dealt with using the option ‘allsnps = TRUE’, in which case every *f4* statistic (on which *qpAdm* admixture estimations are based) is calculated using the intersection of SNPs with no missingness in the four populations involved in the specific estimation, hence every *qpAdm* model is estimated using a unique set of SNPs, which increases the number of sites analysed and conserves statistical power and precision. These strengths of the tool are particularly important for the analyses in the present paper, since the main population group of interest (CYP_Mylouthkia_PPNB) comprises only 3 samples of relatively low coverage (0.031-0.046X).

In our analysis, we have operated *qpAdm* in R (version 4.1.1) using packages *admixtools* and *tidyverse*, for two purposes: (i) to determine the distal (deep) ancestry of all Neolithic populations included in our dataset; and (ii) to determine the proximal ancestry of Cypro-LPPNB Mylouthkia.

(i) Distal ancestry of all Neolithic population groups of interest

For the first purpose, we used Epipaleolithic/Mesolithic populations from southwestern Asia and southeastern Europe, plus a North African population, testing thus all possible sources of ancestry in early Neolithic Near Eastern and the earliest European Neolithic Populations (Table 1, main manuscript; Supplementary Table S1).

The list of populations in distal ancestry model includes: TUR_Pinarbasi_EpiP, ISR_Natufian_EpiP, IRN_C_Zagros_Mes_N, EHG, SRB_Iron_Gates_HG, CHG, MAR_Taforalt_EpiP, WHG, RUS_AfontovaGora3, RUS_MA1_HG, Mbuti.DG, with the latter 4 fixed in the reference list (i.e. used only as references and not as sources). The choice of reference populations is based on the original publication reporting results for the CYP_ Mylouthkia_PPNB samples, in order to aid consistency^66^.

The list of target populations comprised the following populations: CYP_Mylouthkia_PPNB, TUR_C_Boncuklu_PPN, TUR_C_AsikliHoyuk_PPN, TUR_C_Musular_PPN, TUR_Mar_Aktopraklik_N, TUR_C_Catalhoyuk_N, TUR_C_TepecikCiftlik_N, TUR_Mar_Barcin_N, TUR_Mar_Mentse_N, TUR_Mar_Ilipinar_N, ISR_Motza_PPNB, ISR_KfarHaHoresh_PPNB, JOR_AinGhazal_PPNB, JOR_AinGhazal_PPNC, JOR_Baja_PPNB, TUR_Cayonu_PPN, TUR_NevaliCori_PPN, TUR_SE_Mardin_PPNA, IRQ_Nemrik9_PPNA, IRQ_Shanidar_PPN, IRN_Ganj_Dareh_PPN, IRN_Wezmeh_PPN, GRC_ North_Revenia_EN, GRC_North_NeaNikomedeia_EN, GRC_South_Alepotrypa_EN, BGR_Yabalkovo_EN, BGR_Dzhulyunitsa_EN, BGR_MalakPreslavets_EN, ROU_Cotatcu_EN, ROU _Carcea_EN, ALB_Podgorie_EN, MKD_Govrlevo_EN, SRB_LepenskiVir_EN, HRV_Zemunica_Cardial_EN, HUN_Tiszapuspoki_Koros_EN, ITA_GrottaContinenza_EN.

It should be noted that population group ‘IRN_C_Zagros_Mes_N’ meant to represent Mesolithic Central Zagros, is actually composed of a relatively low coverage Mesolithic sample from HotuIIIb (Central Zagros) and a higher coverage early Neolithic sample from Tepe Abdul Hosein (Central Zagros)^42^, who is positioned very closely to the aforementioned Mesolithic individual in our PCA plot (Fig 2, main manuscript), indicating high genetic affinity (also confirmed via a pairwise *f3*-statistcs run between the two). The reason for combining the two samples in a single population group (IRN_C_Zagros_Mes_N) for the purposes of running our deep ancestry analyses in *qpAdm*, is simply to overcome statistical issues relating to low precision when the low coverage Mesolithic sample is used on its own. With this approach, we thus simulate the possible Central Zagros Mesolithic ancestry, while increasing statistical power, without biasing the estimates. We have tested running the model with and without the Neolithic sample incorporated and results were almost identical, yet with lower precision (higher standard errors) when the Mesolithic sample was used on its own. We have also tested an alternative approach, where we used a different Neolithic source from the same region (Ganj Dareh) to represent Mesolithic Central Zagros, which however gave inferior model fits, particularly for earlier Neolithic groups (including Cypro-LPPNB).

Overall, our rotating model approach resulted in simultaneously testing 245 different models for each target population, each model with a different combination of source populations. Once all models were run, following recent guidelines^100^ we classified models as implausible if: (i) the estimated admixture proportions for the target population fall outside the biologically relevant range (0–1); (ii) the model was rejected statistically with a p-value<0.05; and (iii) the precision was low, as indicated by large estimate standard errors. For the second criterion, it should be noted that some researchers might choose more ‘lenient’ significance levels (e.g. 1% - i.e. rejecting models with a p-value <0.01 only), but in case of the use of a rotating model (see above), where hundreds of different admixture models are tested simultaneously, we strongly support the use of a significance level of 5%, to reduce the possibility of type 1 errors. For the third criterion, it should be noted that there is no specific cut-off for rejecting models based on the size of the estimate standard error, however, as a rule of thumb, we have rejected models with standard errors exceeding 0.10 and interpreted with caution models with standard errors between 0.06-0.10.

From the tested models, one stood out providing plausible admixture profiles for all Neolithic samples tested, comprising Pınarbaşı HG, Levantine Natufian HGs, and the Zagros Mesolithic/Neolithic pair noted above, as deep ancestral population groups. Admixture composition for all tested Neolithic population groups, based on this deep ancestral model can be found in Supplementary Table S3. It should be noted that: (i) for few population groups, CHG provided a slightly better model fit than Central Zagros Mesolithic/Neolithic; and (ii) for a very small number of population groups there was evidence for additional plausible models including very minor Mesolithic Balkan HG admixture, additional to the three aforementioned ancestral components (i.e. four-way admixture). Supplementary Table S3 includes notes, for each tested population group, regarding the above two points.

(ii) Proximal ancestry of Cypro-LPPNB Mylouthkia samples

For our main aim, that is to determine the ancestry composition of Cypro-LPPNB Mylouthkia, we followed a systematic approach, where subsequent models were fit in *qpAdm*, following again a rotating model approach. Our choice of source populations was based on the relevant literature^42, 66^, as well as on findings from our distal model, which revealed (in terms of deep ancestry) the main admixture composition of Cypro-LPPNB, as deriving from an ancestral Epipaleolithic Anatolian source, an Epipaleolithic Levantine source, and a Mesolithic Zagros source. For our proximal ancestry model, we chose to use both Epipaleolithic and PPN Neolithic sources, always making sure that the latter do not postdate the specific Cypro-LPPNB Mylouthkia group.

Each model ran included CYP_Mylouthkia_PPNB as the target population and a combination of the following source populations: (i) an Epipaleolithic Anatolian source (TUR_Pinarbasi_EpiP), (ii) a PPN Anatolian source (one from TUR_C_Boncuklu_PPN, TUR_C_AsikliHoyuk_PPN, TUR_C_Musular_PPN, in turn), (iii) an Epipaleolithic/PPN Levantine source (one from ISR_Natufian_EpiP, ISR_Motza_PPNB, ISR_KfarHaHoresh_PPNB, JOR_AinGhazal_PPNB, JOR_Baja_PPNB, in turn), (iv) a Mesopotamian/Zagros PPN source (one from TUR_SE_NevaliCori_PPNB, TUR_SE_Cayonu_PPNB, TUR_SE_NevaliCori_PPNB, TUR_SE_Mardin_PPNA, IRQ_Nemrik9_PPNA, IRQ_Shanidar_PPNA, in turn). Since we wanted the proximal ancestry model to be as realistic as possible, we did not include among the sources Neolithic populations groups geographically very distant from Cyprus (e.g. those from Central Zagros or the Caucasus), assuming that such ancestry would have reached Cyprus indirectly via more proximal sources (e.g. Upper Mesopotamia and northwestern Zagros), which are in fact included among our sources.

Due to the high number of closely related populations, it was not deemed appropriate to include in a single model, sources from the same region showing evidence of high genetic affinity. Therefore, subsequent models were run, each including a population from each set noted above. For example, different combinations of source populations, for subsequent rotating models could be:

- Model 1: TUR_Pinarbasi_EpiP, TUR_C_Boncuklu_PPN, ISR_Motza_PPNB, IRQ_Nemrik9_PPNA.
- Model 2: TUR_Pinarbasi_EpiP, TUR_C_AsikliHoyuk_PPN_PPN, ISR_Motza_PPNB, IRQ_Nemrik9_PPNA.
- Model 3: TUR_Pinarbasi_EpiP, TUR_C_Musular_PPN, ISR_Motza_PPNB, IRQ_Nemrik9_PPNA.
- Model 4: TUR_Pinarbasi_EpiP, TUR_C_Boncuklu_PPN, JOR_Baja_PPNB, IRQ_Nemrik9_PPNA.
- etc.

The above was followed until all plausible combinations of sources was examined. The reference list for all models included the following populations, anticipated to be differentially related to the sources, as required for a successful *qpAdm* model^100^: Mbuti.DG, MAR_Taforalt_EpiP, RUS_AfontovaGora3, RUS_MA1_HG, WHG, SRB_Iron_Gates_HG, TUR_C_Catalhoyuk_PN, CHG, IRN_Ganj_Dareh_PPN, EHG.

Secondary proximal admixture models were run, with the following Pottery Neolithic population groups included as potential Anatolian sources: TUR_Mar_Aktopraklik_PN, TUR_Mar_Barcin_N, TUR_C_Catalhoyuk_PN. It should be noted that these population groups did not fulfil our criteria for being included as primary sources, as they postdate the analysed Cypro-LPPNB samples (c. 7,600–6,800 BCE) by about half to one millennium (Aktopraklik c. 6,700–6,500 BCE; Barcin c. 6,500–5,900 BCE; Çatalhöyük c. 7,100–6,000 BCE). The rationale for testing these Pottery Neolithic groups as sources for Mylouthkia is for consistency reasons with the previously published analysis on the Cypro-LPPNB Mylouthkia samples^66^, which also included PN populations as sources. For the aforementioned reason (Cypro-LPPNB predates PN Anatolians), results from this secondary analysis should be interpreted with caution, as discussed in the main manuscript.

In order to distinguish plausible from implausible models, we applied the same criteria as described above. All relevant models including potentially ancestral to Cypro-LPPNB populations from Anatolia, the Levant, Upper Mesopotamia, and northwestern Zagros can be found in Supplementary Table S7. The table denotes which models have a good or poor fit, as well as clearly statistically rejected.

(iii) Proximal ancestry of other contemporaneous to Mylouthkia Near Eastern groups for comparative purposes

The above-described rotating model approach was repeated including PN Anatolian population groups, with similar genetic profile to Cypro-LPPNB, namely Marmara (Barcin, Menteşe, Ilıpınar) and Central Anatolian Çatalhöyük, as targets. The purpose of this additional analysis was to determine whether the inferred admixture events giving rise to Cypro-LPPNB Mylouthkia, also explain the ancestry of other roughly contemporaneous populations from the mainland, or whether they are specific to Cypriot Neolithic.

*qpGraph* and *findGraphs*

The tool *qpGraph* included in the package ADMIXTOOLS v.2.0.0^96, 97^, utilizes estimated *f3*-statistics and a given topology (graph shape based on a parameter file) and relevant genetic parameters (drift and admixture weights) to derive an ideal admixture graph, representing admixture dynamics and genetic drift for a given list of population groups (or individual samples). *qpGraph* achieves this by identifying the edges weights that minimize the difference between fitted and estimated *f3*-statistics^96, 101^.

The minimal difference between fitted and estimated *f3*-statistics is summarised in a log-likelihood (LL) score. A good model is expected to fit all *f3*-statistics and have an LL score close to zero. Additionally, fitted and estimated *f4*-statistics are utilised to provide *f4*-statistic residuals and the worst residual z-score (WR score). The WR score is also used as an indication of model fit, with a lower score indicating a better admixture graph fit. It should be noted that the LL score is generally considered a better indicator of the quality of the model fit^97^. A limitation of *qpGraph* is that it evaluates a single graph by finding the best combination of weights for a specific topology, set by the user. As a result, the tool relies on the subjective understanding and perception of the user regarding the anticipated admixture dynamics for a given set of population groups.

The *findGraphs* tool, also within the ADMIXTOOLS v.2.0.0^96, 97^ package, detects the best fitting graph topologies for a set of *f*-statistics, estimated for a given set of ancient populations groups, with the outgroup and number of admixture events set by the user and held constant. In this approach ideal admixture graphs are automatically inferred, either without intervention by the user or with some ‘guidance’, as noted by Meier et al^97^. In a fully automated approach, *findGraphs* starts with a random admixture graph and continuously improves it by making random, as well as targeted changes, continuously testing improvements in model fit.

With this approach, all possible admixture graphs for a given set of population groups and pre-specified number of admixture events could be exhaustively tested, discarding topologically redundant or implausible graphs ^97^. A major advantage of this exhaustive approach is that it empirically tests all graph topologies that are consistent with the data for a specified level of admixture events, which is not biased by the manual topology manually set by the user (see *qpGraph*). According to the developers, *findGraphs* can ‘get stuck’ in attempting to improve a non-ideal randomly chosen graph, particularly in cases where many populations and admixture events are set. Therefore, it is recommended to run *findGraphs* several times, as each run will begin with a different random graph. This increases the chances of identifying optimal and plausible graphs.

A limitation of the automated *findGraphs* approach, is that it can robustly and reliably be applied only for relatively simple admixture graphs, usually not exceeding 6 population groups and a small number of admixture events. More complex admixture graphs including a high number of population groups and admixture events will lead to an exponential increase in the number of statistically plausible admixture graphs, involving usually archaeologically implausible admixture events, with overfitted resulting graphs^97^.

(i) Automated construction of admixture graphs for Cypro-LPPNB Mylouthkia

For investigating admixture dynamics involving the samples of interest (Cypro-LPPNB Kissonerga-*Mylouthkia*) the recently proposed protocol for fitting admixture graphs in archaeogenetic studies^97^ was followed. The proposed ‘automated’ admixture graph fitting approach was applied using *findGraphs* in R (version 4.1.1) using packages *admixtools*. *tidyverse*, and *magrittr*. Initially, *f2*-statistics blocks were derived directly from genotype files (Eigenstrat format). requires a selection of populations, which in this case included all potential sources for Cypro-LPPNB, as identified in *qpAdm* (Epipaleolithic Natufian, Epipaleolithic Pinarbaşi, PPN Boncuklu Höyük, and Levant PPNB), as well as other more distal sources (e.g. Central Zagros Mesolithic/Neolithic and CHG), treating Mbuti.DG as the outgroup population.

Following the protocol by Maier et al.^97^, an initial scan was carried out using *findGraphs* to identify the ideal graph complexity class (i.e. ideal number of allowed admixture events), by setting option ‘numadmix’ from 3 (the minimum number of known admixture events among the included populations) to 8, with 100 algorithm iterations per graph complexity class. checking model fit based on the LL score and the WR score.

As a second step, *findGraphs* was ran again on the chosen complexity class (in this case 3-5 admixture events) and saving those with the best model fit (smallest LL score and WR score) after each iteration, applying bootstrapping to detect the graphs that fit significantly better than the rest. Topological constraints were also set to ‘demand’ admixture events for PPN populations groups (Cypro-LPPNB Mylouthkia, PPN Boncuklu, and Levant PPNB). The list of all good fitting admixture graphs was then inspected manually to evaluate their archaeological/archaeogenetic plausibility (*findGraphs* might provide good fitting graphs where very ancient populations appear to be the result of admixture between more recent populations - e.g. Epipaleolithic Natufians appearing as a mix between a basal ancestral population and Levant PPNB, rather than *vice versa*). Implausible models with such features were identified and rejected.

The final admixture graphs were then compared to each other for identifying common features (e.g. graph topology and in particular the admixture event giving rise to Cypro-LPPNB based on each graph). According to Maier et al.^97^, once a set of fitting graphs and stable topological features shared between them is identified, and assuming that these involve different numbers of admixture events, the graphs with the minimal number of admixture events and not substantially worse fit (based on LL score and WR score), are preferred.

The above automated procedure was repeated, including PPN Aşıklı Höyük instead of PPN Boncuklu Höyük, in order to investigate whether Boncuklu is the best fitting Anatolian source for Cypro-LPPNB Mylouthkia, as revealed in *qpAdm* analysis (see above). As with the main analysis, the best fitting admixture graphs from this secondary analysis were saved and their fit and graph topology plausibility, was compared with the original set of derived admixture graphs.

The specificity of the derived admixture graphs for Cypro-LPPNB Mylouthkia (i.e. whether the inferred admixture dynamics that gave rise to the Mylouthkia samples are specific to their ancestry or can successfully be applied to other roughly contemporaneous populations from Anatolia) was also investigated, by repeating the exact same automated admixture graph analysis with the following two populations in the place of Cypro-LPPNB Mylouthkia, in turn: (i) Çatalhöyük, and (ii) Barcin Höyük.

(ii) Manual construction of admixture graph for Cypro-LPPNB Mylouthkia

In addition to the above automated approach, *qpGraph* was applied by manually setting the graph topology based on *qpAdm* findings (see previous sub-section) on the admixture composition of Cypro-LPPNB and other population groups of interest (see below). In this case, the topology specified an outgroup (Mbuti.DG) and included all populations included in the automated approach described above (Epipaleolithic Natufian, Epipaleolithic Pinarbaşi, Central Zagros Mesolithic/Neolithic, CHG, PPN Boncuklu Höyük, and Levant PPNB). The manual graph topology was set to 3 admixture events: one between Epipaleolithic Pinarbaşi and Central Zagros Mesolithic/Neolithic giving rise to Boncuklu; one between Natufians and Boncuklu giving rise to Levant PPNB; and one between Natufians and Boncuklu giving rise to Cypro-LPPNB. *qpGraph* was applied using default parameters with option ‘return_fstats’ set to TRUE.

*Distribution of Ancestry Tracts of Evolutionary Signals (DATES)*

We applied the Distribution of Ancestry Tracts of Evolutionary Signals (DATES) tool to infer the timing of the admixture event for all plausible 2-way admixture models for Cypro-LPPNB, as identified in *qpAdm* (see sub-section above)^102^. DATES utilises the weighted ancestry covariance patterns across the genome of a target population to infer the time of admixture from two source populations. Inferred admixture timing is provided in generations prior to the date the target samples have lived. Generations are converted to years by assuming a mean generation time of 28 years ^102, 103^. In order to derive an absolute chronological date for the admixture event (e.g. in years BCE), the sampling age of the target ancient genomes is added.

An important aspect of DATES is that it does not require phased data and can be effectively used with pseudo-haploid (instead of diploid) genotype calls, making it suitable for ancient DNA data. Another feature particularly important for the present study, is its robustness to small sample sizes and large numbers of missing SNPs in the source/reference populations, the target population, or both^102^.

Model fit is estimated through the normalized root-mean-square deviation (NRMSD) between the empirical ancestry covariance values z and the fitted values, across all the genetic distance bins^102^. The developers of the tool and method, propose the following (not definite) criteria for rendering estimated dates of admixture ‘significant’: (a) Z-score > 2; (b) time of admixture (λ) < 200 generations; and (c) NRMSD < 0.7.

DATES was applied according to the developers’ recommendations, with default options and binsize: 0.001, maxdis: 0.7 (also testing other options, e.g. 0.5, 1.0, etc., which provided slightly poorer model fits), qbin: 10 (also testing other values, with no substantial differences in estimates or model fit), jackknife: YES, lovalfit: 0.45, and minparentcount: 1.

The tool was initially applied for estimating admixture timing for Cypro-LPPNB Mylouthkia, based on the best-fitting 2-way admixture models as identified in *qpAdm*. Following the tool’s developers’ recommendations^102^, in addition to using admixture pairs comprising individual population groups as sources, closely related groups involved in the ancestry of a specific target (e.g. TUR_C_Boncuklu_PPN and TUR_Pinarbasi_EpiP or Levant_PPNB and ISR_Natufian_EpiP) were combined in a single group, to obtain a more precise estimate of admixture timing.

Following the inference of admixture timing for Cypro-LPPNB Mylouthkia, admixture timings were also inferred for other surrounding populations from the mainland, including Central Anatolian PPN groups (TUR_C_Boncuklu_PPN, TUR_C_AsikliHoyuk_PPN, TUR_C_Musular_PPN), SE Anatolian / Upper Mesopotamian PPN groups (TUR_SE_NevaliCori_PPNB, TUR_SE_Cayonu_PPNB, TUR_SE_Mardin_PPNA), Levantine PPNB groups, and northwestern Anatolian (Marmara) groups combined, in order to determine whether the inferred admixture timing for the latter is unique and specific and does not characterise other roughly contemporaneous groups from the surrounding mainland. For the same purpose, the best-fitting model for Cypro-LPPNB, was also applied to PN Anatolian Marmara groups, who appear to have a similar genetic profile to Mylouthkia. These additional analyses serve another important purpose, that is to derive a plausible timeframe for the emergence of the Cypro-LPPNB Mylouthkia group within the surrounding genetic landscape of the early Neolithic Near East, with relatively high certainty.

*Relationship Estimation from Ancient DNA (READ)*

The tool READ^104^ was utilised to infer family relationships for the three Cypro-LPPNB Kissonerga-*Mylouthkia* samples of interest (I4207, I4209, I4210). READ is designed to estimate the degree of relationship for pairs of ancient samples based on pseudo-haploid data (i.e. one randomly sampled allele per individual and SNP site), which is common in low coverage ancient genomes. READ achieves this by dividing the corresponding genomes into non-overlapping windows of 1 Mbps, calculating the proportion of non-matching alleles within each window (termed ‘P0’), for each. Initially, P0 is normalised using the pairwise allele differences among unrelated individuals within the population (α), to deal with within population diversity, SNP ascertainment and marker density, based on the median of all average pairwise P0 across all sampled individuals, which is expected to correspond to a pair of unrelated individuals, given a sufficiently large sample. For a given ‘α’, the P0 for two identical genomes will be α/2, therefore identical segments of the genome from samples belonging to the same individual can be detected.

Following the above-described approach, READ infers kinship and outputs the best fitting degree of relationship for each pair of tested samples as: ‘identical’ (i.e. two samples from same individual or identical twins)’; ‘second-degree relatives’ (i.e. nephew/niece-uncle/aunt, grandparent-grandchild or half-siblings); ‘first-degree relatives’ (parent-offspring or siblings); or ‘unrelated’. This classification is based on the point estimate of the average P0, which according to the tool’s developers^104^, as well as independent evaluations^105^, shows consistent inference of kin relationships and a low number of false positives, even at low genome coverages (0.1-0.5X). The output of READ also includes the uncertainties for a given kinship estimation, such as the distance to the classification cutoffs, expressed as multiples of the standard error of the mean (Z).

**9. References**

1. Simmons, A. H. Akrotiri-Aetokremnos (Cyprus) 20 years later: an assessment of its significance. *Eurasian Prehistory* **10**, 139-156 (2013).

2. Vigne, J., Briois, F. & Zazzo, A. A new early pre-pottery neolithic site in Cyprus: Ayios tychonas-klimonas (ca. 8700 cal BC). *Neo-Lithics* **1**, 3-18 (2011).

3. Manning, S. W., McCartney, C., Kromer, B. & Stewart, S. T. The earlier Neolithic in Cyprus: recognition and dating of a Pre-Pottery Neolithic A occupation. *Antiquity* **84**, 693-706 (2010).

4. Vigne, J. *et al*. First wave of cultivators spread to Cyprus at least 10,600 y ago. *Proceedings of the National Academy of Sciences* **109**, 8445-8449 (2023).

5. Guilaine, J. *et al*. The Pre-pottery Neolithic settlement at Shillourokambos (Parekklisha, Cyprus). **126**, 590-597 (2002).

6. Peltenburg, E. J. The Colonisation and Settlement of Cyprus: Investigations at Kissonerga-Mylouthkia, 1976-1996. (P. Åströms förlag, Sävedalen, 2003).

7. Knapp, A. B. Chronology, current research and interpretative context. in *The Archaeology of Cyprus: From Earliest Prehistory Through the Bronze Age* (Cambridge University Press, Cambridge, 2013).

8. Manning, S. W. New Radiocarbon Chronology for Prehistoric and Protohistoric Cyprus, ca. 11,000–1050 Cal BC. in *The archaeology of Cyprus: from earliest prehistory through the Bronze Age* (ed Knapp, A. B.) 485–518 (Cambridge University Press, Cambridge, 2013).

9. Reimer, P. J. *et al*. IntCal09 and Marine09 Radiocarbon Age Calibration Curves, 0–50,000 Years cal BP. *Radiocarbon* **51**, 1111-1150 (2009).

10. Efstratiou, N. The late Epipalaeolithic camp site of Vretsia-Roudias in upland Troodos: the third season of fieldwork (2011). *Report of the Department of Antiquities, Cyprus* (2017).

11. Ammerman, A. J. The Submerged Final Palaeolithic of Aspros Dive Site C. in *The Archaeology of Europe’s Drowned Landscapes* (eds Bailey, G., Galanidou, N., Peeters, H., Jöns, H. & Mennenga, M.) 429-442 (Springer International Publishing, Cham, 2020).

12. Knapp, A. B. Maritime Narratives of Prehistoric Cyprus: Seafaring as Everyday Practice. *Journal of Maritime Archaeology* **15**, 415-450 (2020).

13. Clarke, J. & Wasse, A. Time out of joint: A re-assessment of the Cypriot Aceramic Neolithic site of Kalavasos-Tenta and its regional implications. *Levant* **51**, 26-53 (2019).

14. Sevketoglu, M. & Hanson, I. Akanthou-Arkosykos, a ninth Millenium BC coastal settlement in Cyprus*. Environmental Archaeology* **20**, 225-238 (2015).

15. Simmons, A. H., DiBenedetto, K. & Keach, L. Neolithic Kritou Marottou-*Ais Giorkis*, Cyprus—Living in the Uplands. *Bulletin of the American Schools of Oriental Research* **379**, 171-195 (2018).

16. Knapp, A. B. *The Archaeology of Cyprus: From Earliest Prehistory Through the Bronze Age* (Cambridge University Press, Cambridge, 2013).

17. Introduction. in *The Colonisation and Settlement of Cyprus: Investigations at Kissonerga-Mylouthkia, 1976-1996* (ed Peltenburg, E.) xxxiii–xxxvii (P. Åströms förlag, Sävedalen, 2003).

18. Croft, P. The Wells and Other Vestiges. in *The Colonisation and Settlement of Cyprus: Investigations at Kissonerga-Mylouthkia, 1976-1996* (ed Peltenburg, E. J.) 3-9 (P. Åströms förlag., Sävedalen, 2003).

19. Peltenburg, E. Conclusions: Mylouthkia 1 and the Early Colonists of Cyprus. in *The Colonisation and Settlement of Cyprus: Investigations at Kissonerga-Mylouthkia, 1976-1996* (ed Peltenburg, E.) 83-103 (P. Åströms förlag, Sävedalen, 2003).

20. Fox, S. C., Lunt, D. A. & Watt, M. E. Human remains. in *The Colonisation and Settlement of Cyprus: Investigations at Kissonerga-Mylouthkia, 1976-1996* (ed Peltenburg, E. J.) 221-224 (P. Åströms förlag., Sävedalen, 2003).

21. Lorentz, K. O. External auditory exostoses and early Neolithic aquatic resource procurement in Cyprus: Results from Cypro-PPNB Kissonerga-Mylouthkia in regional context. *International Journal of Paleopathology* **30**, 98-104 (2020).

22. Cucchi, T., Vigne, J., Auffray, J., Croft, P. & Peltenburg, E. Introduction involontaire de la souris domestique (Mus musculus domesticus) à Chypre dès le Néolithique précéramique ancien (fin IXe et VIIIe millénaires av. J.-C.). *Comptes Rendus Palevol* **1**, 235-241 (2002).

23. McCartney, C. & Gratuze, B. The Chipped Stone. in *The Colonisation and Settlement of Cyprus: Investigations at Kissonerga-Mylouthkia, 1976-1996 Studies in Mediterranean Archaeology* (ed Peltenburg, E. J.) 11-30 (P. Åströms förlag, Sävedalen, 2003).

24. Scott, E. M., Naysmith, P. & Cook, G. T. Why do we need 14C inter-comparisons?: The Glasgow -14C inter-comparison series, a reflection over 30 years. *Quaternary Geochronology* **43**, 72-82 (2018).

25. Falconer, S. E., Ridder, E., Pilaar Birch, S. E. & Fall, P. L. PREHISTORIC BRONZE AGE RADIOCARBON CHRONOLOGY AT POLITIKO-TROULLIA, CYPRUS. *Radiocarbon* **65**, 97-119 (2023).

26. Stuiver, M. *et al*. INTCAL98 Radiocarbon Age Calibration, 24,000–0 cal BP. *Radiocarbon* **40**, 1041-1083 (1998).

27. Reimer, P. J. *et al*. The IntCal20 Northern Hemisphere Radiocarbon Age Calibration Curve (0–55 cal kBP). *Radiocarbon* **62**, 725-757 (2020).

28. Bronk Ramsey, C. Bayesian Analysis of Radiocarbon Dates. *Radiocarbon* **51**, 337-360 (2009).

29. Croft, P. The Animal Bones. in *The colonisation and settlement of Cyprus: investigations at Kissonerga-Mylouthkia, 1976-1996* (ed Peltenburg, E. J.) 49-58 (P. Åströms förlag., Sävedalen, 2003).

30. Lazaridis, I. *et al*. The genetic history of the Southern Arc: A bridge between West Asia and Europe. *Science* **377** (2022).

31. Peltenburg, E., Croft, P., Jackson, A., McCartney, C. & Murray, M. A. Well-Established Colonists: Mylouthkia 1 and the Cypro-Pre-Pottery Neolithic B. in *The Earliest Prehistory of Cyprus: From Colonization to Exploitation (CAARI Monograph Series 2)* (ed Swiny, S.) 61-94 (American Schools of Oriental Research, Boston, 2001).

32. Aitken, M. J. in *Science-based Dating in Archaeology* 219-220 (Longman, London, 1990).

33. Meiklejohn, C., Chamberlain, A. & Schulting, R. Radiocarbon dating of Mesolithic human remains in Great Britain. *Mesolithic Miscellany* **21**, 20-58 (2011).

34. Conard, N. J., Grootes, P. M. & Smith, F. H. Unexpectedly recent dates for human remains from Vogelherd. *Nature* **430**, 198-201 (2004).

35. Bonsall, C. & Boroneanț, A. The Iron Gates Mesolithic – a brief review of recent developments. . *L'Anthropologie* **122**, 264-280 (2018).

36. Baird, D. *et al*. Agricultural origins on the Anatolian plateau. *Proceedings of the National Academy of Sciences* **115**, E3077-E3086 (2018).

37. Bar-Yosef, O. & Valla, F. R. Natufian Foragers in the Levant: Terminal Pleistocene social changes in Western Asia. (Berghahn Books, New York, 2013).

38. Vahdati Nasab, H., Shirvani, S. & Rigaud, S. The Northern Iranian Central Plateau at the End of the Pleistocene and Early Holocene: The Emergence of Domestication. *Journal of World Prehistory* **32**, 287-310 (2019).

39. Tejero, J. *et al*. New insights into the Upper Palaeolithic of the Caucasus through the study of personal ornaments. Teeth and bones pendants from Satsurblia and Dzudzuana caves (Imereti, Georgia). *PLoS One* **16**, e0258974 (2021).

40. Mathieson, I. *et al*. The genomic history of southeastern Europe. *Nature* **555**, 197-203 (2018).

41. Feldman, M. *et al*. Late Pleistocene human genome suggests a local origin for the first farmers of central Anatolia. *Nature Communications* **10**, 1218 (2019).

42. Lazaridis, I. *et al*. Genomic insights into the origin of farming in the ancient Near East. *Nature* **536**, 419-424 (2016).

43. Narasimhan, V. M. *et al*. The formation of human populations in South and Central Asia. *Science* **365**, eaat7487 (2019).

44. Jones, E. R. *et al*. Upper Palaeolithic genomes reveal deep roots of modern Eurasians. *Nature Communications* **6**, 8912 (2015).

45. van de Loosdrecht, M. *et al*. Pleistocene North African genomes link Near Eastern and sub-Saharan African human populations. *Science* **360**, 548-552 (2018).

46. Kılınç, G. M. *et al*. Archaeogenomic analysis of the first steps of Neolithization in Anatolia and the Aegean. *Proc.Biol.Sci.* **284**, 20172064 (2017).

47. Mathieson, I. *et al*. Genome-wide patterns of selection in 230 ancient Eurasians. *Nature* **528**, 499-503 (2015).

48. Marchi, N. *et al*. The genomic origins of the world’s first farmers. *Cell* **185**, 1859.e18 (2022).

49. Posth, C. *et al*. Palaeogenomics of Upper Palaeolithic to Neolithic European hunter-gatherers. *Nature* **615**, 117-126 (2023).

50. Zeder, M. A. The Origins of Agriculture in the Near East. *Curr.Anthropol.* **52**, S221-S235 (2011).

51. Willcox, G., Buxó, R. & Linda, H. Late Pleistocene and Early Holocene climate and the beginnings of cultivation in northern Syria. *The Holocene* **19**, 151-158 (2009).

52. Bellwood, P. *First Farmers: The Origins of Agricultural Societies* (Wiley-Blackwell, Hoboken, 2023).

53. Blockley, S. P. E. & Pinhasi, R. A revised chronology for the adoption of agriculture in the Southern Levant and the role of Lateglacial climatic change. *Quaternary Science Reviews* **30**, 98-108 (2011).

54. Price, T. D. & Bar-Yosef, O. An Introduction to Supplement 4; The Origins of Agriculture: New Data, New Ideas. *Curr.Anthropol.* **52**, S163-S174 (2011).

55. Wang, X. *et al*. Isotopic and DNA analyses reveal multiscale PPNB mobility and migration across Southeastern Anatolia and the Southern Levant. *Proceedings of the National Academy of Sciences* **120** (2023).

56. Rollefson, G. O., Simmons, A. H. & Kafafi, Z. Neolithic Cultures at 'Ain Ghazal, Jordan. *Journal of Field Archaeology* **19**, 443-470 (1992).

57. Peters, J., Pöllath, N. & Arbuckle, B. S. in *The Oxford Handbook of Zooarchaeology* (eds Albarella, U., Rizzetto, M., Russ, H., Vickers, K. & Viner-Daniels, S.) 247-265 (Oxford University Press, Oxford, 2017).

58. Altınışık, E. *et al*. A genomic snapshot of demographic and cultural dynamism in Upper Mesopotamia during the Neolithic Transition. *Science Advances* **8** (2022).

59. Dietrich, O., Köksal-Schmidt, Ç, Kürkçüoğlu, C., Notroff, J. & Schmidt, K. Göbekli Tepe - A Stone Age ritual center in southeastern Turkey. *Actual Archaeology Magazine* **2**, 32-51. (2012).

60. Barbaro, C., Iamoni, M., Morandi, D., Moscone, D. & Qasim, H. The Prehistory and Protohistory of the northwestern region of Iraqi Kurdistan: Preliminary results from the first survey campaigns. *Paléorient* **45**, 207-229 (2019).

61. Broushaki, F. *et al*. Early Neolithic genomes from the eastern Fertile Crescent. *Science* **353**, 499-503 (2016).

62. Yaka, R. *et al*. Variable kinship patterns in Neolithic Anatolia revealed by ancient genomes. *Current Biology* **31**, 2468.e18 (2021).

63. Koptekin, D. *et al*. Spatial and temporal heterogeneity in human mobility patterns in Holocene Southwest Asia and the East Mediterranean. *Current Biology* **33**, 41-57.e15 (2023).

64. Kilinc, G. *et al*. The Demographic Development of the First Farmers in Anatolia. *Current Biology* **26**, 1-8 (2016).

65. Karul, N. The beginning and the development of farming-based village life in Northwestern Anatolia. in *6000 BC: Transformation and Change in the Near East and Europe* (eds Biehl, P. F. & Rosenstock, E.) 231-246 (Cambridge University Press, Cambridge, 2022).

66. Lazaridis, I. *et al*. Ancient DNA from Mesopotamia suggests distinct Pre-Pottery and Pottery Neolithic migrations into Anatolia. *Science* **377**, 982-987 (2022).

67. Vigne, J., Guilaine, J., Debue, K., Haye, L. & Gérard, P. Early Taming of the Cat in Cyprus. *Science (New York, N.Y.)* *Science* **304**, 259 (2004).

68. Vigne, J., Carrère, I., Briois, F. & Guilaine, J. The Early Process of Mammal Domestication in the Near East: New Evidence from the Pre-Neolithic and Pre-Pottery Neolithic in Cyprus. *Curr.Anthropol.* **52**, S255-S271 (2023).

69. Moutsiou, T. Climate, environment and cognition in the colonisation of the Eastern Mediterranean islands during the Pleistocene. *JMA* **577**, 1-14 (2021).

70. Vigne, J. *et al*. The transportation of mammals to Cyprus sheds light on early voyaging and boats in the Mediterranean Sea. *Eurasian Prehistory* **10**, 157-176 (2014).

71. Bar-Yosef Mayer, D. E., Kahanov, Y., Roskin, J. & Gildor, H. Neolithic Voyages to Cyprus: Wind Patterns, Routes, and Mechanisms. *The Journal of Island and Coastal Archaeology* **10**, 412-435 (2015).

72. Moutsiou, T. The Obsidian Evidence for Trans-maritime Interactions in the Eastern Mediterranean: The View from Aceramic Neolithic Cyprus. *JMA* **31**, 229-248 (2023).

73. Kyriakidis, P. *et al*. Virtual Sea-Drifting Experiments between the Island of Cyprus and the Surrounding Mainland in the Early Prehistoric Eastern Mediterranean. *Heritage* **5**, 3081-3099 (2022).

74. Hofmanová, Z. *et al*. Early farmers from across Europe directly descended from Neolithic Aegeans. *Proceedings of the National Academy of Sciences* **113**, 6886-6891 (2023).

75. Rohrlach, A. B. *et al*. Using Y-chromosome capture enrichment to resolve haplogroup H2 shows new evidence for a two-path Neolithic expansion to Western Europe. *Scientific Reports* **11**, 15005 (2021).

76. Vigne, J. *et al*. Pre-Neolithic wild boar management and introduction to Cyprus more than 11,400 years ago. *National Academy of Sciences* **106**, 16135-16138 (2023).

77. Moutsiou, T., Reepmeyer, C., Kassianidou, V., Zomeni, Z. & Agapiou, A. Modelling the Pleistocene colonisation of Eastern Mediterranean islandscapes. *Plos One* **16**, e0258370 (2021).

78. Okyar, M., Ergin, M. & Evans, G. Seismic stratigraphy of Late Quaternary sediments of western Mersin Bay shelf, (NE Mediterranean Sea). *Mar.Geol.* **220**, 113-130 (2005).

79. Lambeck, K. & Purcell, A. Sea-level change in the Mediterranean Sea since the LGM: model predictions for tectonically stable areas. *Quaternary Science Reviews* **24**, 1969-1988 (2005).

80. Tchernov, E. & Valla, F. F. Two New Dogs, and Other Natufian Dogs, from the Southern Levant. *Archaeological Science* **24**, 65-95 (1997).

81. Weissbrod, L. *et al*. Origins of house mice in ecological niches created by settled hunter-gatherers in the Levant 15,000 y ago. *Proceedings of the National Academy of Sciences* **114**, 4099-4104 (2017).

82. Marshall, F. Cats as predators and early domesticates in ancient human landscapes. *Proceedings of the National Academy of Sciences* **117**, 18154-18156 (2020).

83. Wasse, A. & Clarke, J. Mesopotamian fallow deer and the chase in later Neolithic Cyprus: insights from upper Mesopotamia, the Levant and badia. *Journal of Archaeological Science: Reports* **48**, 103909 (2023).

84. Weiss, E. & Zohary, D. The Neolithic Southwest Asian Founder Crops: Their Biology and Archaeobotany. *Curr.Anthropol.* **52**, S237-S254 (2011).

85. Abbo, S., Lev-Yadun, S. & Gopher, A. Plant Domestication in the Near East. in *Encyclopaedia of the History of Science, Technology, and Medicine in Non-Western Cultures* (ed Selin, H.) 1-9 (Springer Netherlands, Dordrecht, 2008).

86. Mallick, S. *et al*. The Allen Ancient DNA Resource (AADR) a curated compendium of ancient human genomes. *Scientific Data* **11**, 182 (2024).

87. Harrison, P. W. *et al*. The European Nucleotide Archive in 2020. *Nucleic Acids Res.* **49**, D82-D85 (2020).

88. Danecek, P. *et al*. Twelve years of SAMtools and BCFtools. *Gigascience* **10**, giab008 (2021).

89. Patterson, N., Price, A. L. & Reich, D. Population Structure and Eigenanalysis. *Plos Genetics* **2**, e190 (2006).

90. Price, A. L. *et al*. Principal components analysis corrects for stratification in genome-wide association studies. *Nat.Genet.* **38**, 904-909 (2006).

91. Olalde, I. *et al*. Derived immune and ancestral pigmentation alleles in a 7,000-year-old Mesolithic European. *Nature* **507**, 225-228 (2014).

92. Fu, Q. *et al*. The genetic history of Ice Age Europe. *Nature* **534**, 200-205 (2016).

93. Lazaridis, I. *et al*. Ancient human genomes suggest three ancestral populations for present-day Europeans. *Nature* **513**, 409-413 (2014).

94. Lipson, M. *et al*. Parallel palaeogenomic transects reveal complex genetic history of early European farmers. *Nature* **551**, 368-372 (2017).

95. Antonio, M. L. *et al*. Ancient Rome: A genetic crossroads of Europe and the Mediterranean. *Science* **366**, 708-714 (2019).

96. Patterson, N. *et al*. Ancient Admixture in Human History. *Genetics* **192**, 1065-1093 (2012).

97. Maier, R. *et al*. On the limits of fitting complex models of population history to f-statistics. *eLife* **12**, e85492 (2023).

98. R Core Team. R: A language and environment for statistical computing. (2021).

99. RStudio Team. RStudio: Integrated Development Environment for R. (2022).

100. Harney, É, Patterson, N., Reich, D. & Wakeley, J. Assessing the performance of qpAdm: a statistical tool for studying population admixture. *Genetics* **217**, iyaa045 (2021).

101. Lipson, M. Applying f4-statistics and admixture graphs: Theory and examples. *Mol Ecol Resour* **20**, 1658-1667 (2020).

102. Chintalapati, M., Patterson, N. & Moorjani, P. The spatiotemporal patterns of major human admixture events during the European Holocene. *eLife* **11** (2022).

103. Moorjani, P. *et al*. A genetic method for dating ancient genomes provides a direct estimate of human generation interval in the last 45,000 years. *Proceedings of the National Academy of Sciences* **113**, 5652-5657 (2023).

104. Monroy Kuhn, J. M., Jakobsson, M. & Günther, T. Estimating genetic kin relationships in prehistoric populations. *PLoS One* **13**, e0195491 (2018).

105. Marsh, W. A., Brace, S. & Barnes, I. Inferring biological kinship in ancient datasets: comparing the response of ancient DNA-specific software packages to low coverage data. *BMC Genomics* **24**, 111 (2023).

**9. Supplementary Figures**

**Supplementary Figure S1: Ratio of basal Levant to Zagros ancestry among analysed Pre-Pottery Neolithic populations.**


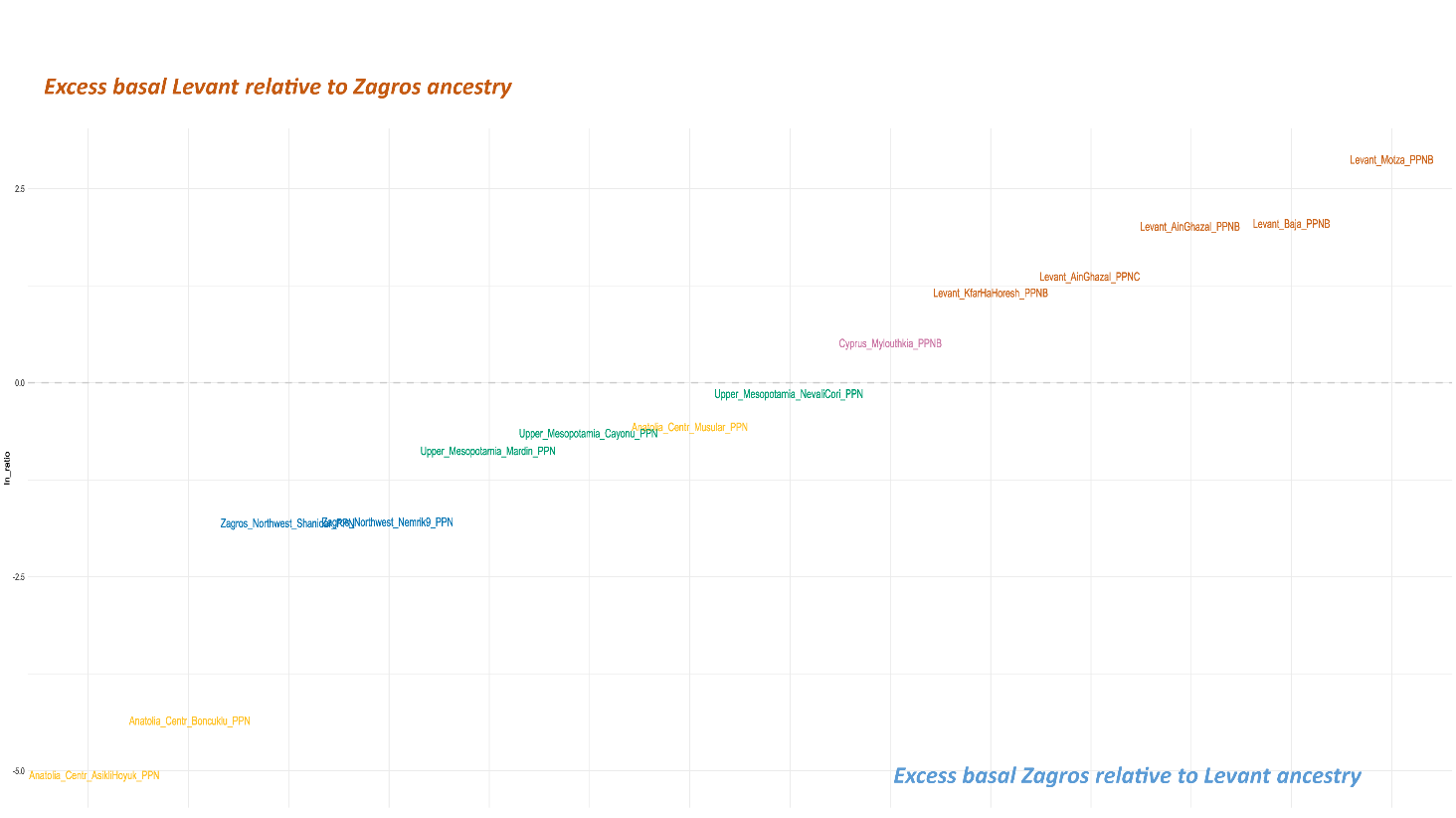


The plot displays the natural log of the ratio (ln_ratio) of the admixture proportion from an ancestral Levantine source (ISR_Natufian_EpiP) to the admixture proportion from an ancestral Central Zagros source (IRN_C_Zagros_Mes_N), as estimated using a distal ancestry *qpAdm* model. The plot focuses only on PPN populations contemporaneous or earlier than Cypro-LPPNB. Positive values indicate excess basal admixture from the Levant relative to the Central Zagros. Negative values indicate excess basal admixture from the Central Zagros relative to the Levant. This scale, being a ratio, estimates excess admixture among analysed populations in relative and not absolute terms. The analysed Cypro-LPPNB show excess Levantine relative to Zagros distal ancestry, in contrast to contemporaneous Central Anatolian and Upper Mesopotamian population groups. Colour-coding represents geographical regions as denoted in Fig. 1. All abbreviations in the plot as in Table 1. The admixture estimates on which these calculations were made can be found in tabular form in Supplementary Table S3.

**Supplementary Figure S2: Outgroup *f3*-statistics (Mbuti; test, comparison) displaying pairwise genetic distances between Cypro-LPPNB and all Epipaleolithic/Mesolithic and Neolithic population groups included in the study.**


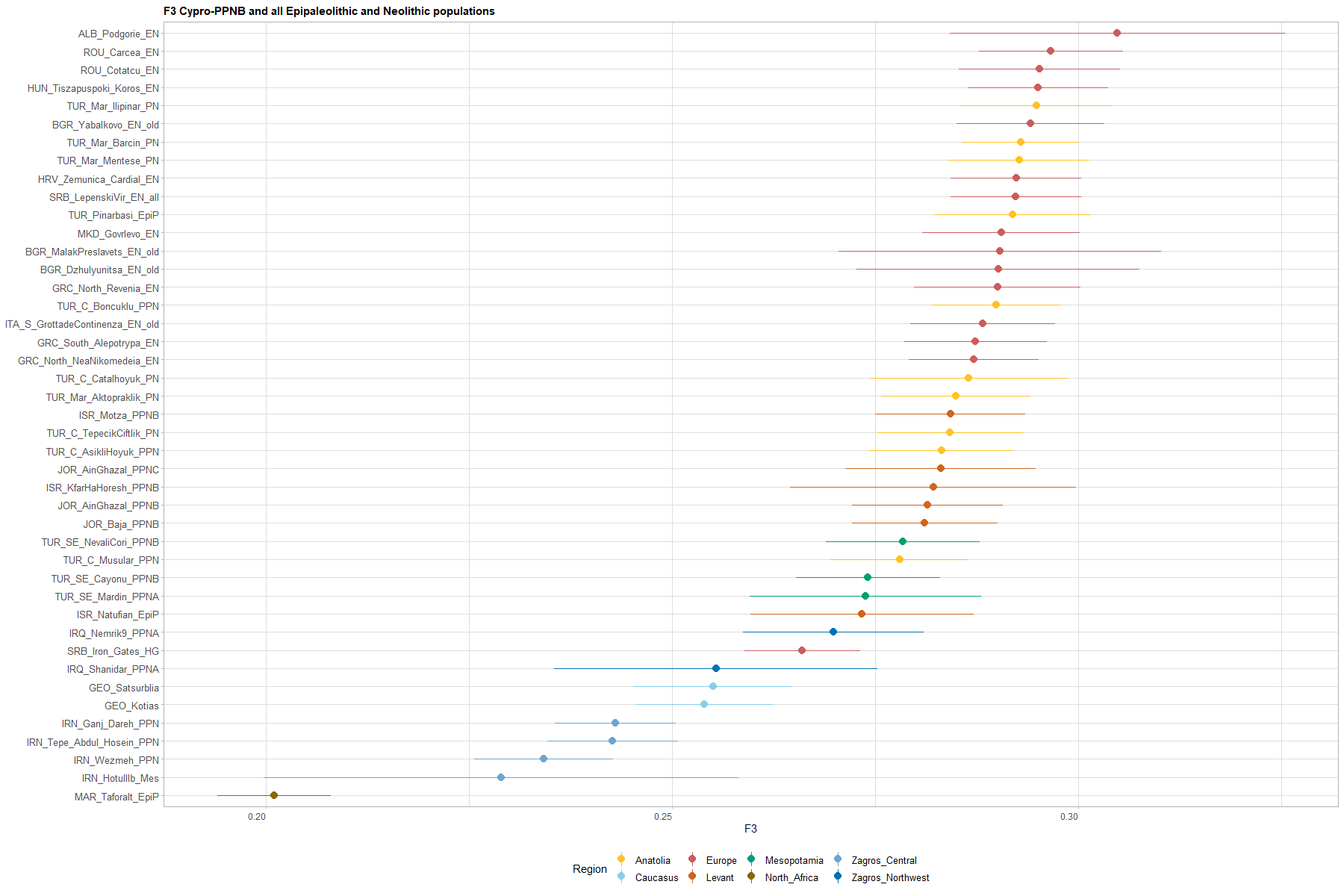


The plot displays pairwise *f3* statistics (±3 standard errors) between Cypro-PPNB and all tested ancient populations. The further to the right the points are in each plot, the higher the allele sharing with Cypro-PPNB. Wider error bars indicate lower precision, as a result of smaller number of available SNPs in the given pairwise comparison. Colour-coding represents geographical regions as denoted in Fig. 1. Cypro-PPNB show high allele sharing with Central Anatolian Epipaleolithic Pınarbaşı HG, Central Anatolian PPN Boncuklu Höyük, Northwest Anatolian PN Marmara groups, and the earliest Neolithic European farmers. *F3* results with low precision (e.g. HotuIIIb) should be interpreted with caution, due to low number of available SNPs. All abbreviations in the plot as in Table 1. These results are also presented by region in main Figure 4. The displayed information can be found in tabular form in Supplementary Table 4.

**Supplementary Figure S3: MDS plot based on outgroup *f3* (Mbuti; test, comparison) displaying two-dimensional genetic distances between all Near Eastern Epipaleolithic/Mesolithic/Neolithic and earliest European Neolithic populations.**


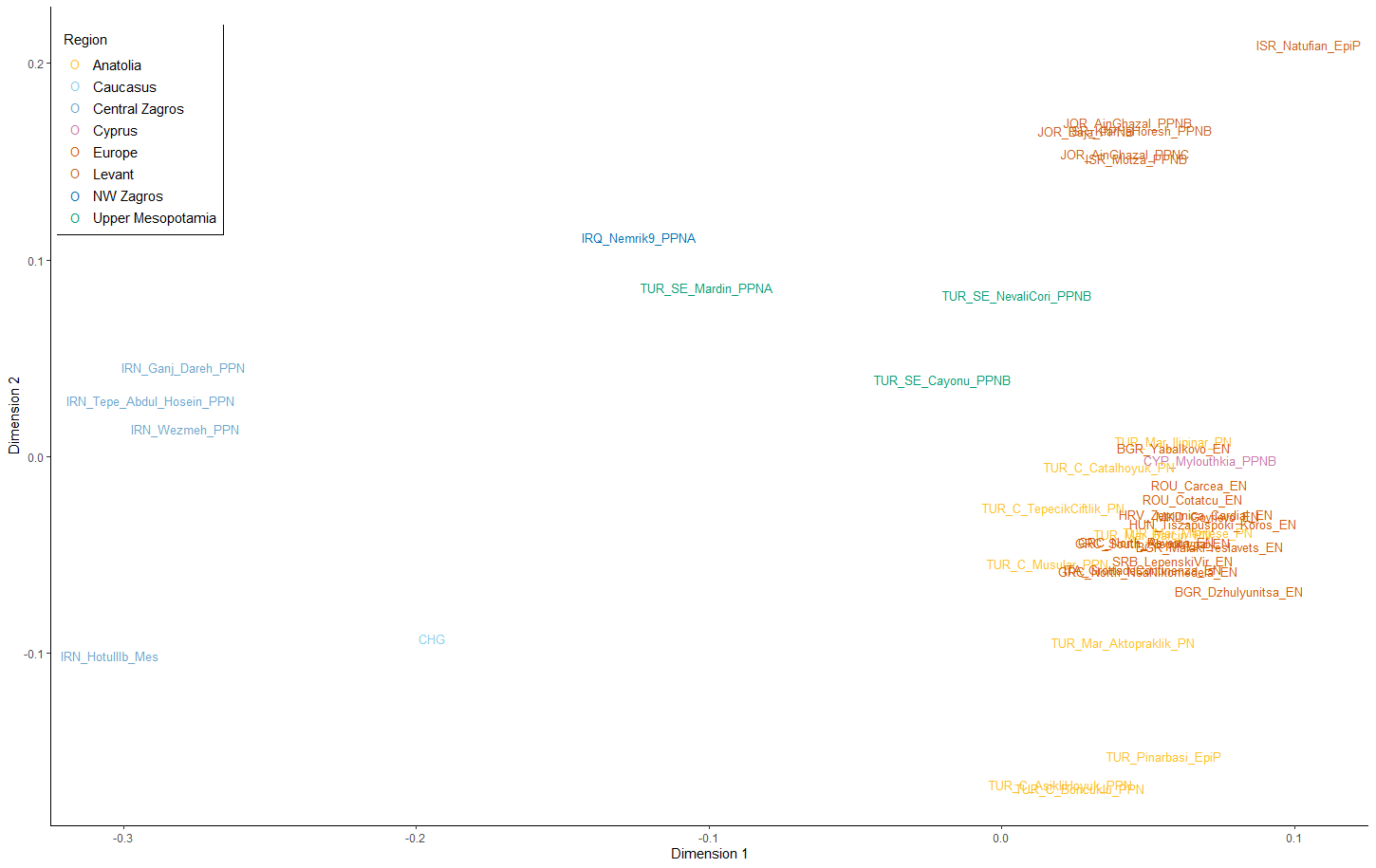


MDS plot based on pairwise outgroup *f3*-statistics of the form *f3* (Mbuti; test, comparison) displaying genetic differentiation based on allele sharing among Mesolithic/Epipaleolithic and Neolithic Near Easterners and the earliest European farmers. Epipaleolithic/Mesolithic HGs (Levantine Natufians, Anatolian Pınarbaşı HG, Iran Hotu IIIb, and CHG) can be seen at the edges of the plot, while Anatolian and very early European farmers, as well as PPNB Cypriots, cluster within a genetic cline between Epipaleolithic Pınarbaşı HG and Levantine Natufians. Colour-coding represents geographical regions as denoted in Fig. 1. All abbreviations in the plot as in Table 1. A matrix of raw genetic distances of the form 1 - *f3* can be found in Supplementary Table S5.

**Supplementary Figure S4: *f4*-statistics displaying shared genetic drift between potential Epipaleolithic/Mesolithic/PPN Near Eastern sources and Cypro-LPPNB.**


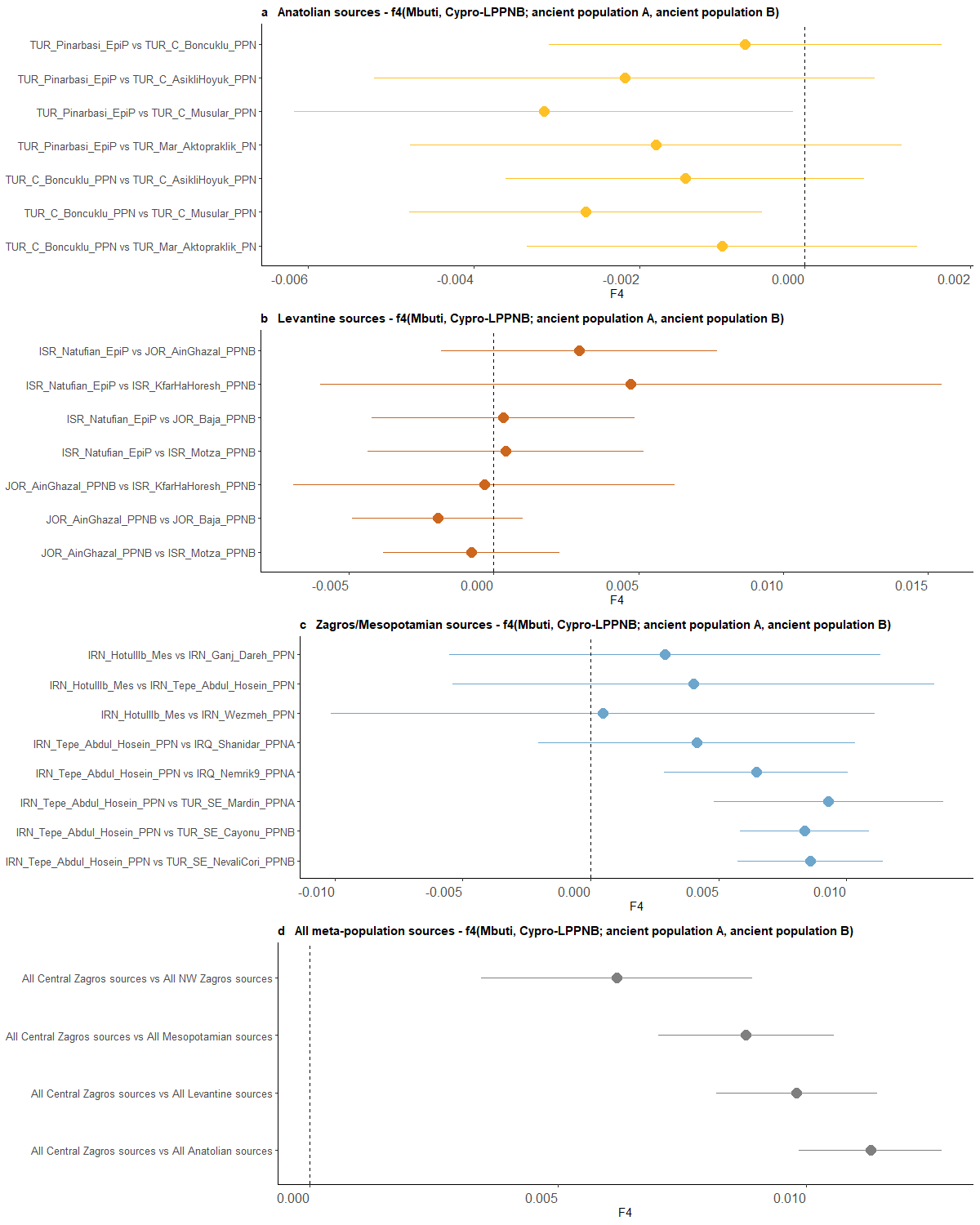


Plot of *f4*-statistics of the form *f4(Mbuti, Cypro-PPNB;* *ancient population A, ancient population B)* ±3 standard errors, estimating shared genetic drift, based on allele sharing. Cypro-PPNB is treated as the ‘test’ population. Ancient populations A and B comprise, in turn, Epipaleolithic/Mesolithic and PPN population sources from Anatolia (panel a), the Levant (panel b), Upper Mesopotamia and the Zagros (panel c), and meta-populations from all aforementioned regions (panel d). Meta populations in panel d are as follows: All Central Zagros sources (IRN_HotuIIIb_Mes, IRN_Ganj_Dareh_PPN, IRN_Tepe_Abdul_Hosein_PPN, IRN_Wezmeh_PPN); All Northwestern Zagros sources (IRQ_Shanidar_PPNA, IRQ_Nemrik9_PPNA); All Upper Mesopotamian sources (TUR_SE_Mardin_PPNA, TUR_SE_Cayonu_PPNB, TUR_SE_NevaliCori_PPNB). All Levantine sources (ISR_Natufian_EpiP, JOR_AinGhazal_PPNB, ISR_KfarHaHoresh_PPNB, JOR_Baja_PPNB, ISR_Motza_PPNB); All Central Anatolian sources (TUR_Pinarbasi_EpiP, TUR_C_Boncuklu_PPN, TUR_C_AsikliHoyuk_PPN). Negative *f4* values indicate higher allele sharing between Cypro-LPPNB and population A rather than population B. Positive values indicate the opposite, while a null *f4* indicates the same level of allele sharing. Colour-coding represents geographical regions as denoted in Fig. 1. All abbreviations in the plot as in Table 1. The displayed information can be found in tabular form in Supplementary Table S6.

**Supplementary Figure S5: Admixture graphs involving Cypro-LPPNB, constructed following a manually arranged topology using *qpGraph* (panel a) or with an automated approach using *findGraphs*, involving 3 admixture events (panels b-e).**


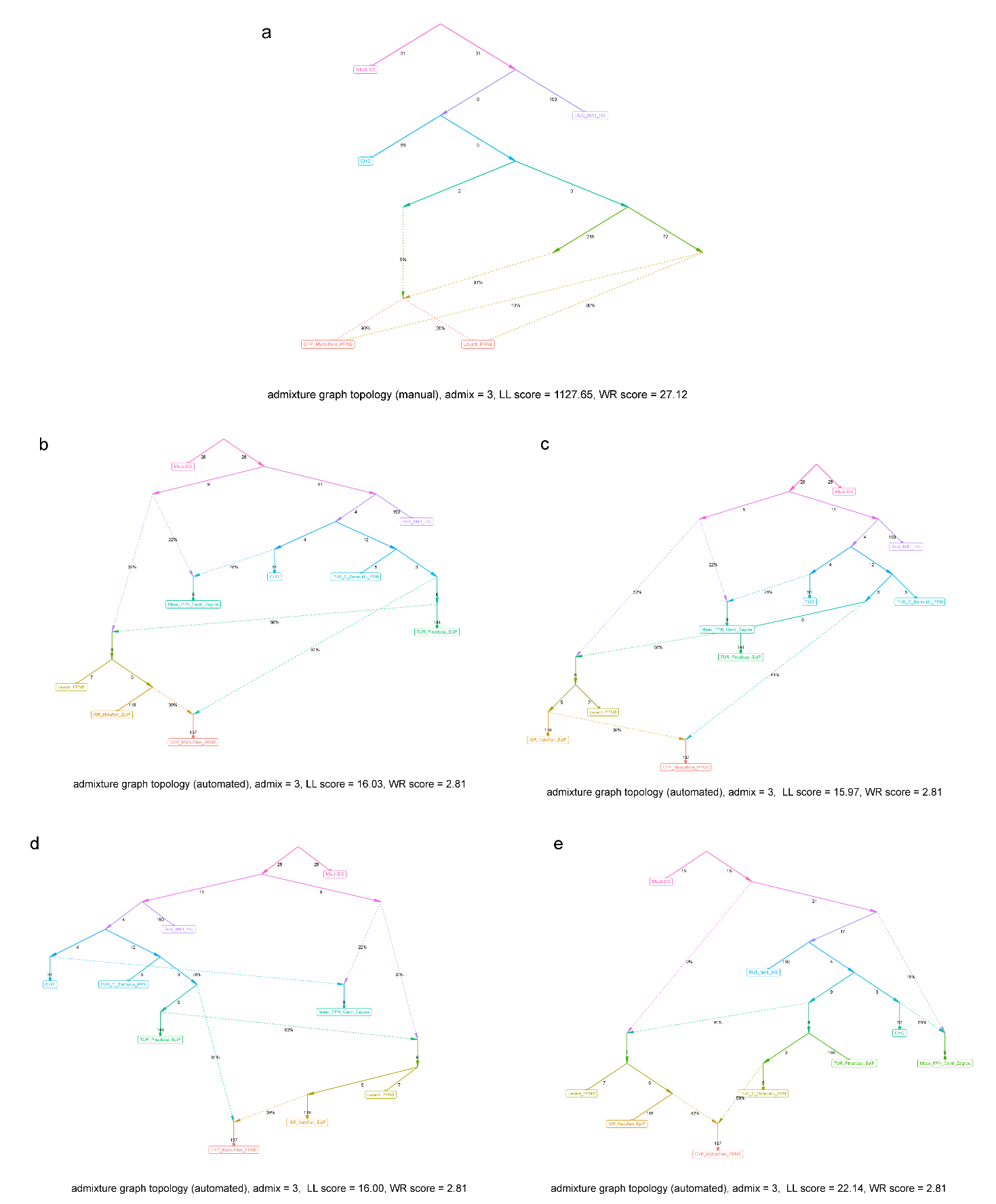


Admixture graphs presenting inferred admixture dynamics between Cypro-LPPNB Mylouthkia and other ancient population groups of interest, allowing for 3 admixture events between the groups comprising the graph topology. Model fit is evaluated based on the log-likelihood (LL) scores and the f-statistic worst residuals (WR) score. The graph in panel (a) is a parsimonious, manually constructed fit using *qpGraph*, based on *qpAdm* findings from the present study, fitting Mylouthkia as a 2-way mix between Levantine Natufians and Central Anatolian Boncuklu, the latter deriving from a mix between Epipaleolithic Pinarbasi and Mesolithic/Neolithic Central Zagros. The specific graph has a poor fit, indicating that alternative admixture dynamics, potentially involving other closely related population groups are involved. The presented graphs in panels b-e are the best fitting following a thorough automated approach with *findGraphs*, where Mylouthkia appear to derive from a sister group to Boncuklu, admixing with a Natufian-like Levantine source, with much improved model fit and an overall admixture graph topology with high plausibility, based on archaeological and archaeogenetic evidence. Proportions on dotted lines represent estimated 2-way admixture weights. Branch lengths on solid lines are in units of genetic distance (FST) × 1000.

**Supplementary Figure S6: Admixture graphs involving Cypro-LPPNB, constructed following an automated approach using *findGraphs*, involving 4 admixture events.**


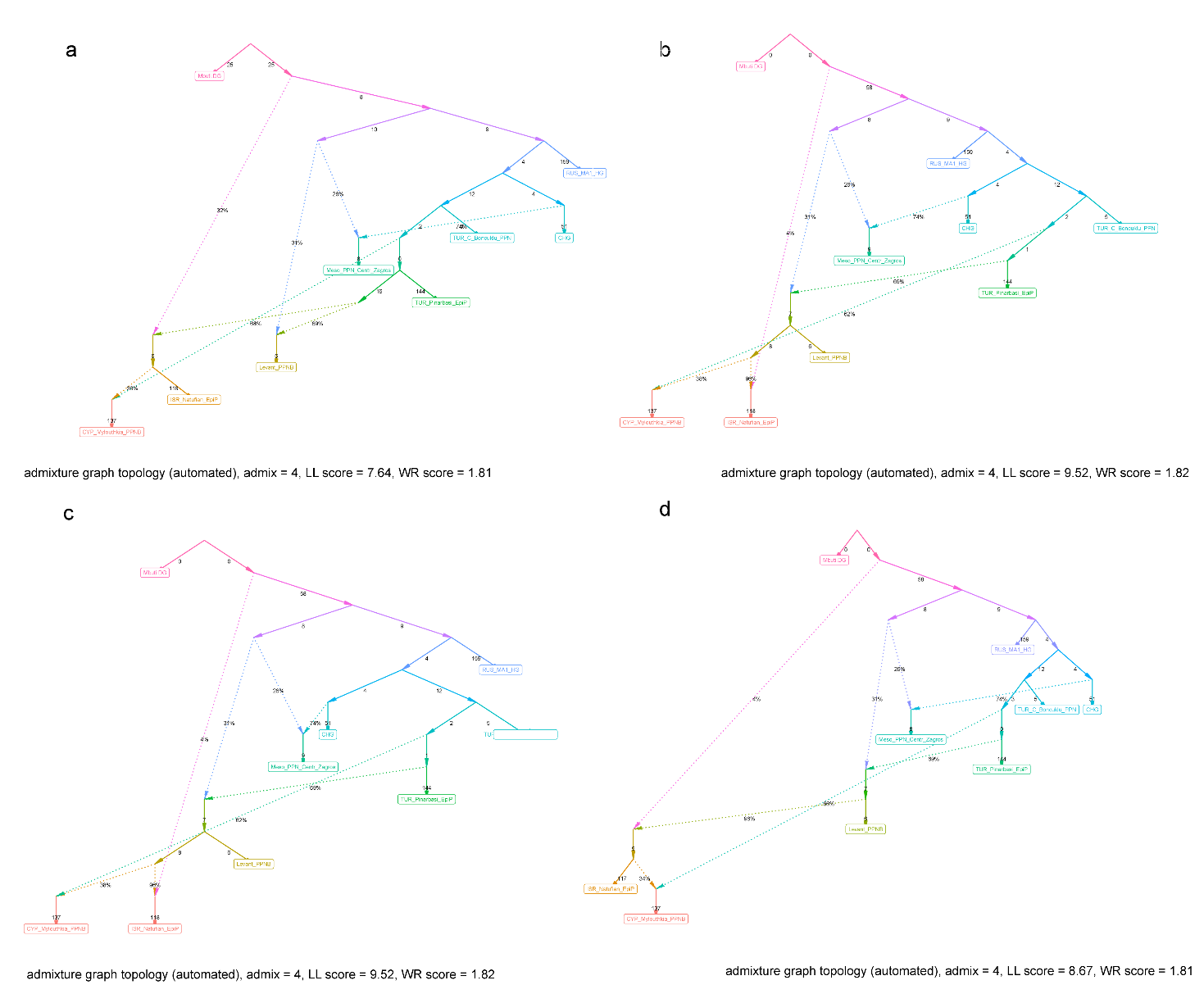


Admixture graphs presenting inferred admixture dynamics between Cypro-LPPNB Mylouthkia and other ancient population groups of interest, allowing for 4 admixture events between the groups comprising the graph topology. Model fit is evaluated based on the log-likelihood (LL) scores and the f-statistic worst residuals (WR) score. The presented graphs (panels a-d) are the best fitting following a thorough automated approach with *findGraphs*, where Mylouthkia appear to derive from a sister group to Boncuklu, admixing with a Natufian-like Levantine source, with a very good model fit and an overall admixture graph topology with high plausibility, based on archaeological and archaeogenetic evidence. Proportions on dotted lines represent estimated 2-way admixture weights. Branch lengths on solid lines are in units of genetic distance (FST) × 1000.

**Supplementary Figure S7: Admixture graphs involving Cypro-LPPNB, constructed following an automated approach using *findGraphs*, involving 5 admixture events.**


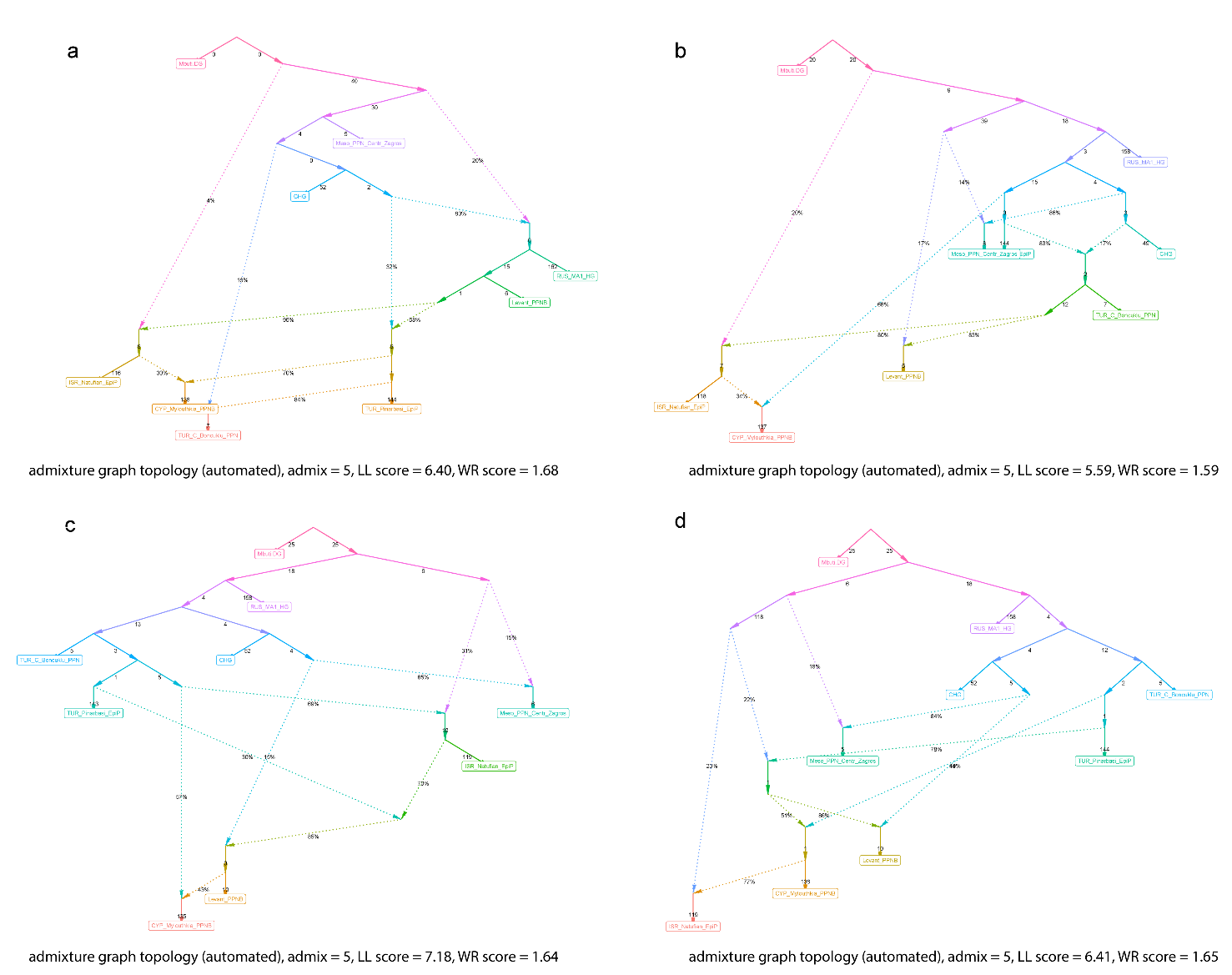


Admixture graphs presenting inferred admixture dynamics between Cypro-LPPNB Mylouthkia and other ancient population groups of interest, allowing for 5 admixture events between the groups comprising the graph topology. Model fit is evaluated based on the log-likelihood (LL) scores and the f-statistic worst residuals (WR) score. The presented graphs (panels a-d) are the best fitting following a thorough automated approach with *findGraphs*, where Mylouthkia appear to derive from either an Epipaleolithic Pinarbasi-related group or a sister group to Boncuklu, admixing with a Natufian-like Levantine source, with a very good model fit and an overall admixture graph topology with moderate to low plausibility, based on archaeological and archaeogenetic evidence. Proportions on dotted lines represent estimated 2-way admixture weights. Branch lengths on solid lines are in units of genetic distance (FST) × 1000.

**Supplementary Figure S8: Admixture graphs involving Cypro-LPPNB, with Aşıklı Höyük as the Central Anatolian source rather than Boncuklu, constructed following an automated approach using *findGraphs*, involving 4 and 5 admixture events.**


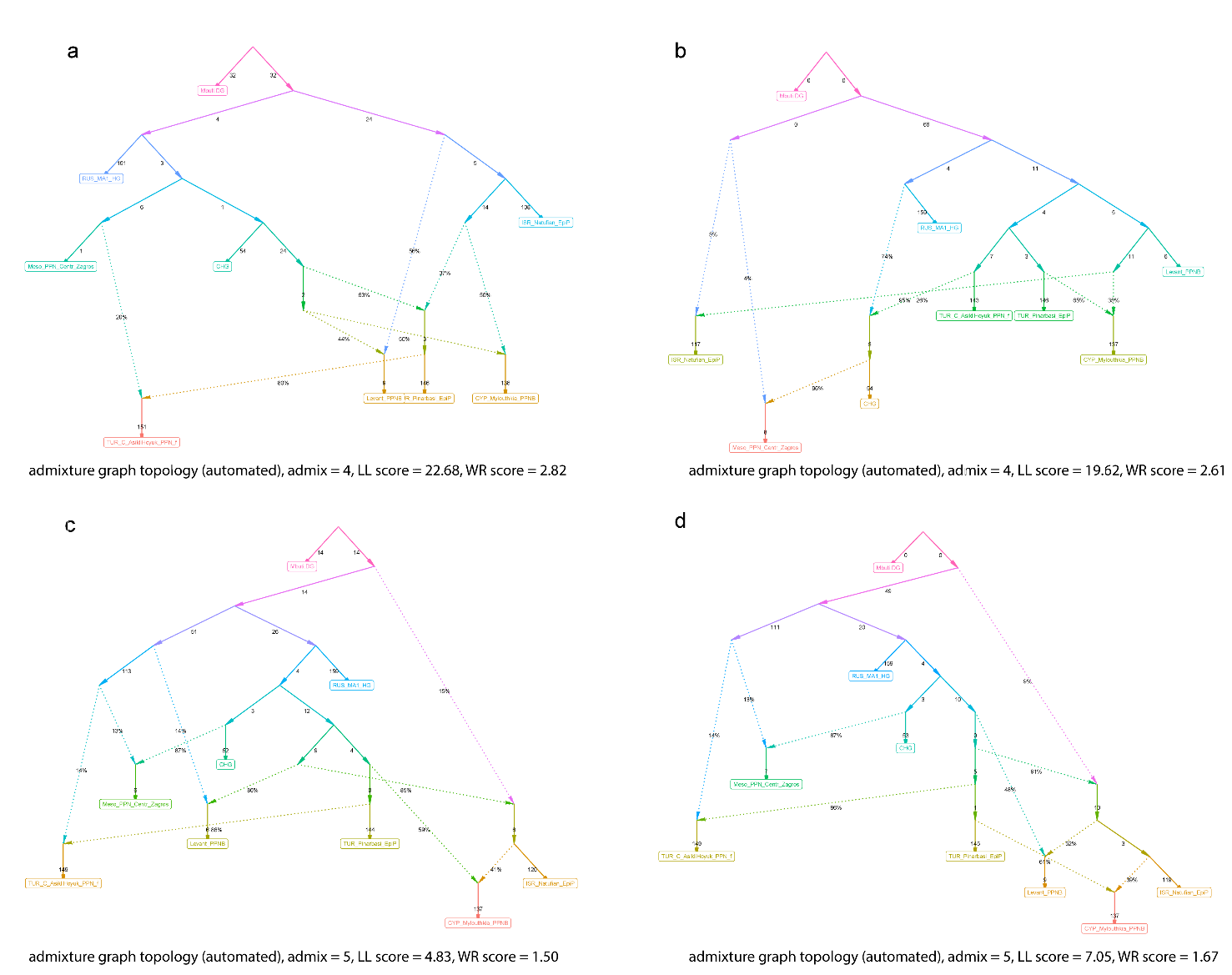


Admixture graphs presenting inferred admixture dynamics between Cypro-LPPNB Mylouthkia and other ancient population groups of interest, including Aşıklı Höyük as the Central Anatolian source rather than Boncuklu and allowing for 4 and 5 admixture events between the groups comprising the graph topology. Model fit is evaluated based on the log-likelihood (LL) scores and the f-statistic worst residuals (WR) score. The presented graphs (panels a-d) are the best fitting following a thorough automated approach with *findGraphs*. In contrast to topologies involving Boncuklu, Aşıklı Höyük is not picked up as a plausible Central Anatolian source for Mylouthkia, who appear to derive primarily from an Epipaleolithic Pinarbasi-related group, admixing with a Natufian-like or PPNB Levantine source, with a moderate model fit and an overall admixture graph topology with moderate to low plausibility, based on archaeological and archaeogenetic evidence. Proportions on dotted lines represent estimated 2-way admixture weights. Branch lengths on solid lines are in units of genetic distance (FST) × 1000.

**Supplementary Figure S9: Admixture graphs involving Çatalhöyük or Barcin instead of Cypro-LPPNB, constructed following an automated approach using *findGraphs*, involving 4 admixture events.**


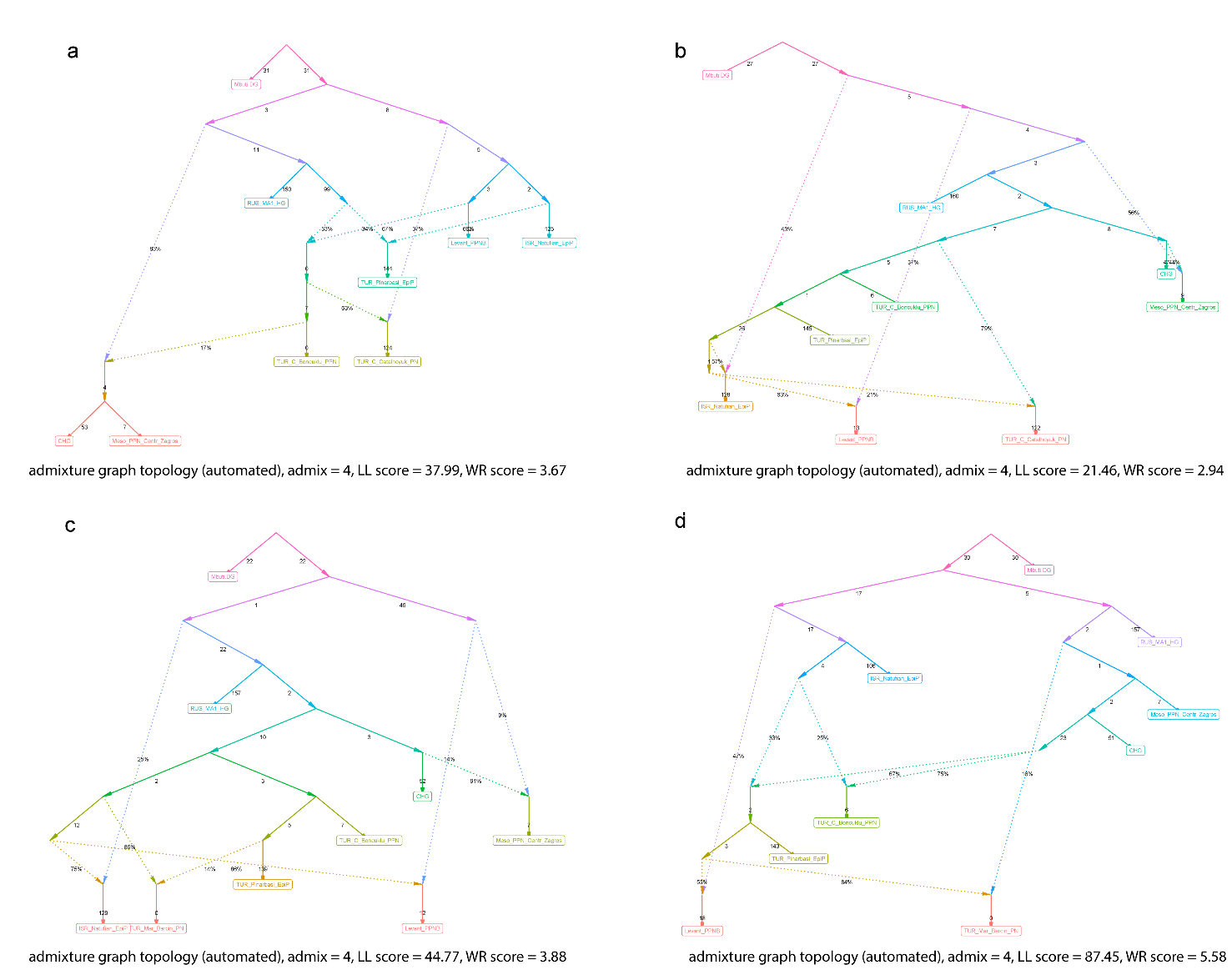


Admixture graphs presenting inferred admixture dynamics involving PN Central Anatolian Çatalhöyük (panels a-b) or PN northwestern Anatolian Barcin (panels c-d), instead of Cypro-LPPNB Mylouthkia, keeping the same list of ancient populations in the graph topology and allowing for 4 admixture events between the tested groups. Model fit is evaluated based on the log-likelihood (LL) scores and the f-statistic worst residuals (WR) score. The presented graphs (panels a-d) are the best fitting following a thorough automated approach with *findGraphs*. In contrast to topologies involving Mylouthkia, the selected list of potentially ancestral populations do not provide good fitting models for either Çatalhöyük or Barcin, indicating that admixture events presented in Supplementary Figures S5-S7, are specific to the ancestry of Mylouthkia and reflect the actual admixture dynamics giving rise to this population group. Proportions on dotted lines represent estimated 2-way admixture weights. Branch lengths on solid lines are in units of genetic distance (FST) × 1000.

**Supplementary Figure S10: Inferred admixture timing ±1 SE for Cypro-LPPNB and other contemporaneous Near Eastern populations.**


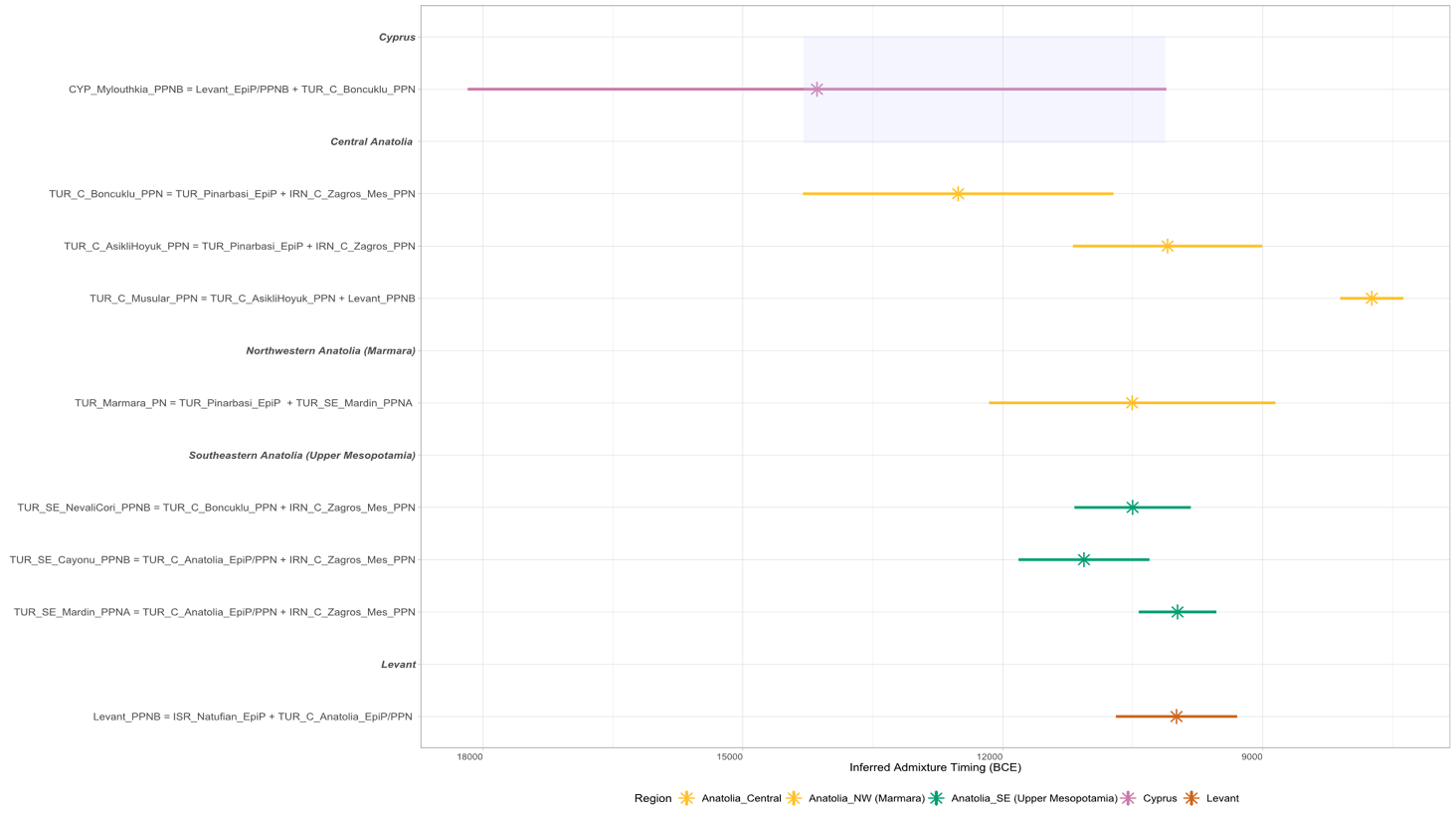


The figure presents inferred time of admixture ±1 standard error (SE) for Cypro-LPPNB Mylouthkia and other contemporaneous populations from Anatolia, Upper Mesopotamia, and the Levant, derived using tool *DATES*. The inferred admixture time frame for Mylouthkia, involving Aceramic Neolithic Boncuklu and Epipaleolithic/Neolithic Levantine groups, is estimated at 18,176 to 10,112 BCE. The estimated admixture timing for other Near Eastern PPN populations, reveals a picture of an early influx of Zagros-related groups into Central Anatolia, contemporary, or even earlier in the case of Boncuklu, to the influx detected in southeastern Anatolia / Upper Mesopotamia. Around the same time (c. 10,000 to 11,500 BCE), and later than the inferred admixture timing for Mylouthkia, groups from Aceramic Neolithic southeastern Anatolia / Upper Mesopotamia and Central Anatolia, appear to have admixed with local Epipaleolithic groups in the Marmara region of Anatolia and the Levant, respectively, giving rise to Pottery Neolithic groups in the specific regions. The inferred admixture time frame for Mylouthkia is wide due to the low coverage of these samples, preventing the inference of highly precise timing estimated. Assuming that the formation of Mylouthkia involves admixture from Central Anatolian Boncuklu (as supported in all analyses from the present study), the range of the admixture timing for this Cypro-LPPNB group can be narrowed down (shaded range), using the upper bound for the formation of Boncuklu, rather than the upper bound inferred for Mylouthkia.

**Supplementary Figure S11: *f4*-statistics displaying shared genetic drift between Cypro-LPPNB samples vs Anatolian PPN/PN populations and very early European Neolithic populations.**


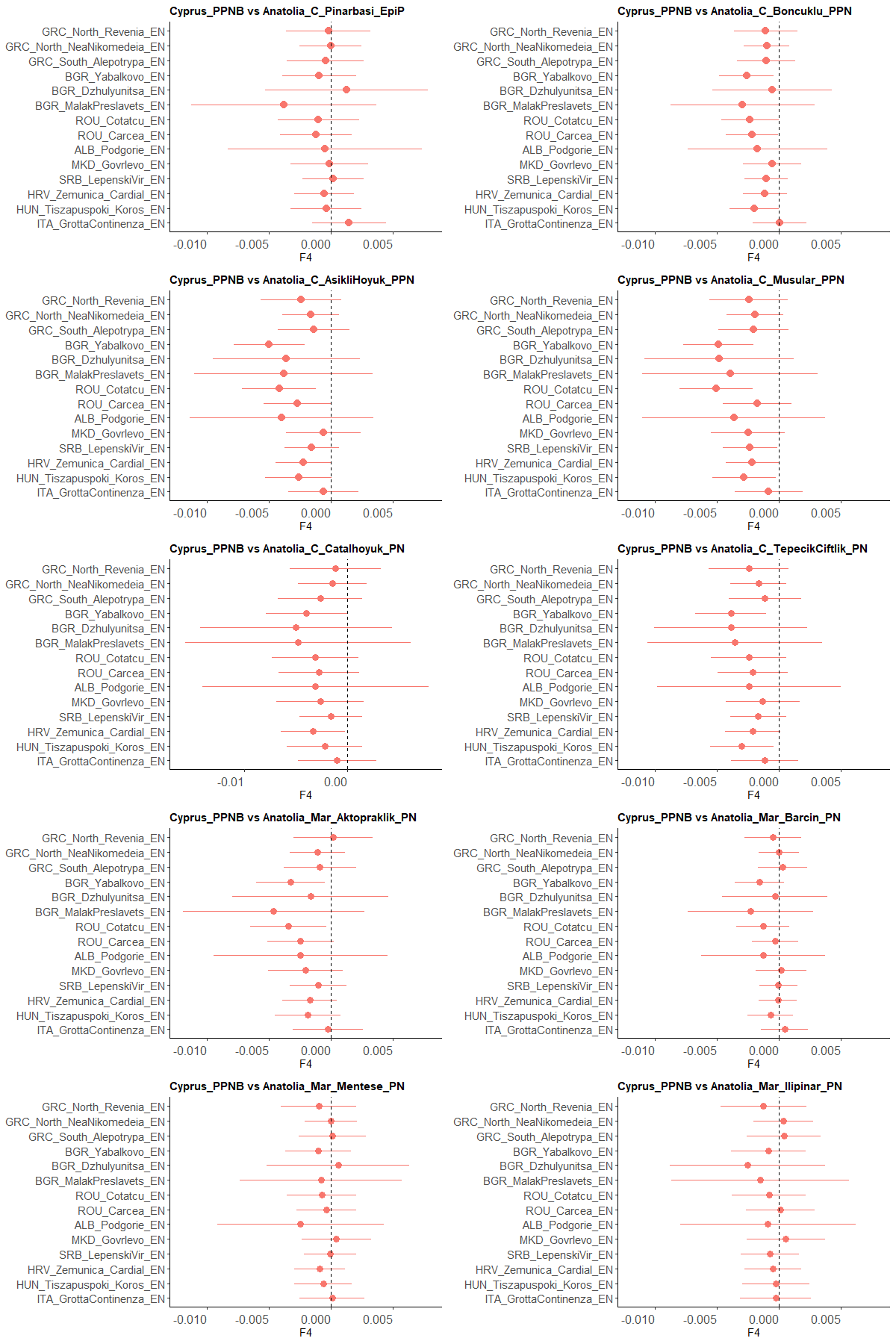


Plot of *f4*-statistics of the form *f4(Mbuti, ancient population A; Cypro-PPNB, ancient population C)* ±3 standard errors, estimating shared genetic drift, based on allele sharing. Ancient population A is treated as the ‘test’ population in this case and includes, in turn, all Very Early European Farming (VEEF) populations used in the current study. Ancient population C includes, in turn, Anatolian PPN and PN populations. Negative *f4* values indicate more allele sharing between Cypro-LPPNB and VEEF than between Anatolians and VEEF. Positive values indicate the opposite, while a null *f4* indicates the same level of allele sharing. Colour-coding represents geographical regions as denoted in Fig. 1. All abbreviations in the plot as in Table 1. The displayed information can be found in tabular form in Supplementary Table S10.

**Supplementary Figure S12:** **Frequency of major Y-chromosome haplogroups among Near Eastern and Southeast European Epipaleolithic/Mesolithic and early Neolithic populations analysed in the present study.**


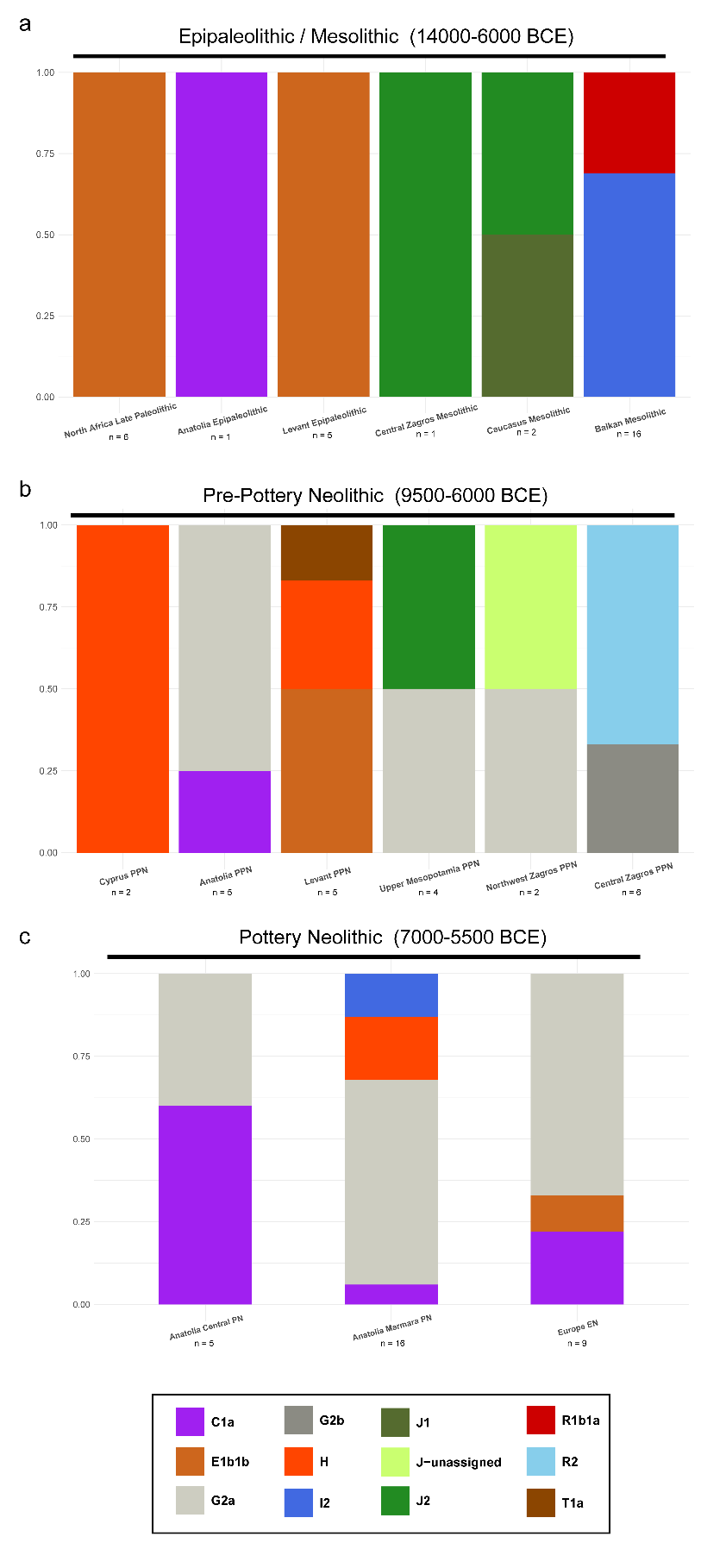


Frequency of major Y-chromosome haplogroups (Y-haplogroups) among Near Eastern and Southeast European Epipaleolithic/Mesolithic and Neolithic populations analysed in the present study, including the Cypro-LPPNB. Only ancient samples included in the present study were considered in the relevant calculations. Haplogroup frequencies for population groups comprising small numbers of individuals (e.g. <10) should be interpreted with caution, as they are not anticipated to accurately represent the true distribution among the corresponding metapopulation. For Cypro-LPPNB, only two informative Y-haplogroups were considered. All abbreviations in the plot as in Table 1. The displayed information can be found in tabular form in Supplementary Table S13.

**Supplementary Figure S13: Frequency of major mitochondrial DNA haplogroups among Near Eastern and Southeast European Epipaleolithic/Mesolithic and early Neolithic populations analysed in the present study.**


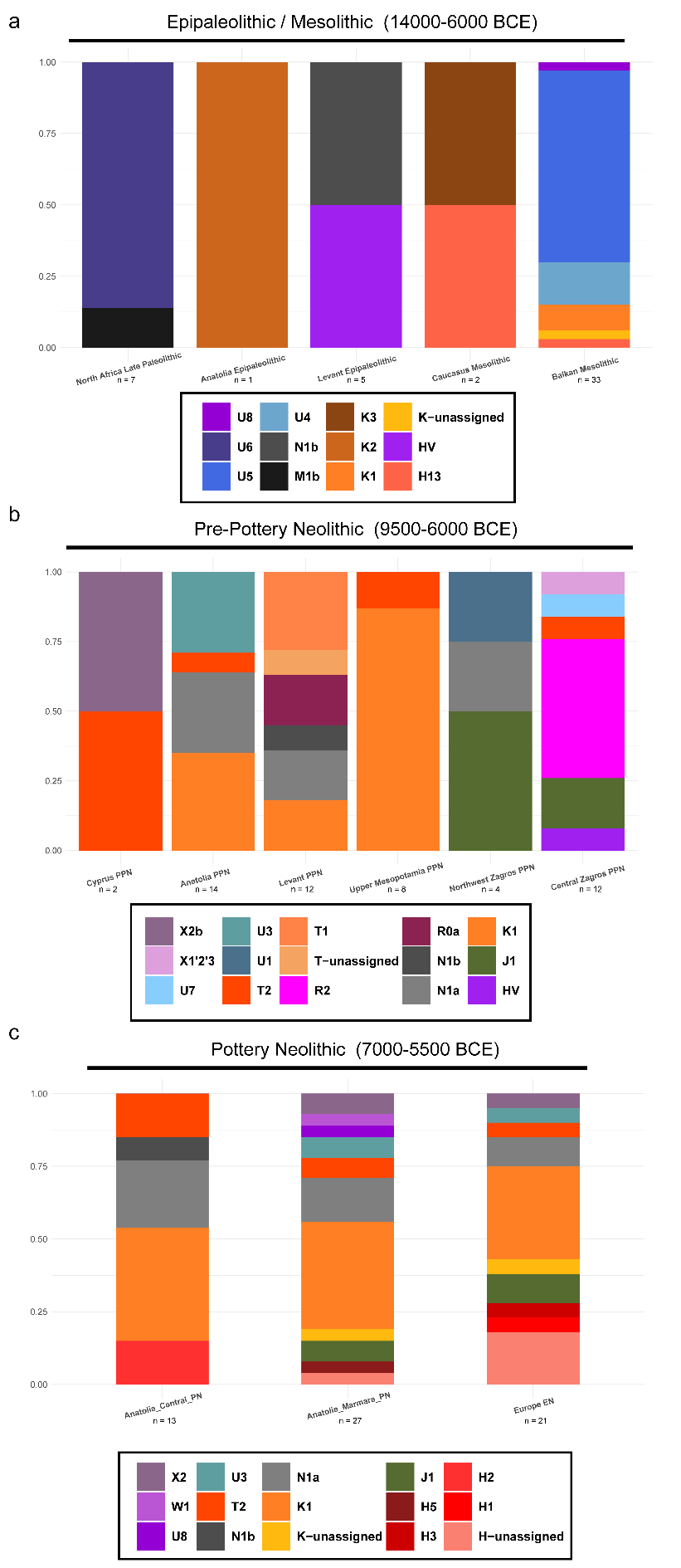


Frequency of major mitochondrial DNA haplogroups (mtDNA-haplogroups) among Near Eastern and Southeast European Epipaleolithic/Mesolithic and Neolithic populations analysed in the present study, including the analysed Cypro-LPPNB. Only ancient samples included in the present study were considered in the relevant calculations. Haplogroup frequencies for population groups comprising small numbers of individuals (e.g. <10) should be interpreted with caution, as they are not anticipated to accurately represent the true distribution among the corresponding metapopulation. For Cypro-LPPNB, only two informative mtDNA-haplogroups were considered. All abbreviations in the plot as in Table 1. The displayed information can be found in tabular form in Supplementary Table S14.
